# Early‐Onset Prostate Cancer: Epidemiology, Risk Factors, Biology, Clinical Features, Management, and Early Detection

**DOI:** 10.1002/mco2.70858

**Published:** 2026-07-22

**Authors:** Xingyu Xiong, Weizhen Zhu, Weichao Huang, Shiyu Zhang, Qi Deng, Jie Yang, Hang Xu, Qiang Wei, Lu Yang

**Affiliations:** ^1^ Department of Urology Institute of Urology West China Hospital of Sichuan University Chengdu Sichuan Province China

**Keywords:** early detection, early‐onset prostate cancer, management, risk factors, survival

## Abstract

The incidence of early‐onset prostate cancer (EOPC) is rising, and by 2045 a 24.5% increase in cases and a 50% rise in mortality are projected. Accumulating evidence indicates that EOPC represents a distinct disease entity, characterized by unique molecular features, risk factor profiles, and clinical behavior that differ from standard‐onset prostate cancer (SOPC). Nevertheless, research in this field remains nascent, and no consensus exists regarding the optimal management of EOPC. We synthesize current evidence on the epidemiology, molecular pathology, clinicopathological characteristics, survival, management, and early detection of EOPC. EOPC exhibits a distinctive molecular landscape, with TMPRSS2–ERG fusions occurring in 63–90% of cases as a hallmark alteration, whereas mutations in PTEN, SPOP, and CHD1 are significantly less frequent. Notably, the prevailing focus on hereditary EOPC has inadvertently led to the neglect of sporadic cases, which dominate clinical practice. Although localized EOPC confers no significant prognostic advantage over SOPC, high‐risk or metastatic early‐onset disease substantially elevates prostate‐cancer‐specific mortality. By critically appraising the existing evidence, we identify key knowledge gaps, such as the understudied sporadic EOPC subgroup and the lack of dedicated clinical trials, and propose future research directions to inform early detection and optimize therapeutic strategies for this unique patient population.

## Graphical abstract

EOPC is a distinct clinicopathological entity separable from SOPC, differing fundamentally in genetic susceptibility, TMPRSS2:ERG fusion frequency, and survival outcomes. Although research remains nascent, this review provides the first comprehensive synthesis of current evidence, offering a critical foundation to guide future investigations into this increasingly important but understudied prostate cancer subtype.

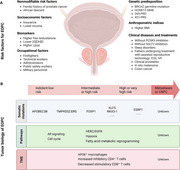

## 1 Introduction

Prostate cancer represents the most prevalent malignancy among males in two‐thirds of countries worldwide [1]. Globally, approximately one in 14 men receives a prostate cancer diagnosis, accounting for 15% of all male cancers (2020) [1, 2, 3]. It ranks as second leading cause of cancer‐related mortality in males, surpassed only by lung cancer [1]. While advanced age constitutes a well‐established risk factor, with over 85% of newly diagnosed cases occurring in individuals aged ≥60 years [4], the mean age at diagnosis has declined from 72 years in 1986 to 67 years in 2009 [5], suggesting a trend toward younger onset. Indeed, global incidence data from 1990 to 2021 demonstrate progressive increases across all age groups, with the sharpest rises observed in men aged 45–49 and 50–54 years [6]. This pattern underscores the growing disease burden of early‐onset prostate cancer (EOPC).

Most contemporary studies define EOPC as diagnosis before the age of 55 years. Accumulating evidence supports its recognition as a distinct clinicopathological subtype, with preliminary data suggesting unique tumor biological behavior. Critically, high‐risk or metastatic EOPC appears to confer substantially elevated mortality risk compared with standard‐onset disease. Given the extended life expectancy of younger patients, developing effective management strategies for this demographic represents an urgent and persistent clinical challenge.

Despite growing recognition of its clinical importance, the precise etiology of EOPC remains incompletely elucidated. While established risk factors such as family history and ethnicity exert stronger effects in early‐onset disease, the underlying mechanisms driving these associations are poorly understood. Moreover, implementing effective early detection strategies, a critical approach to mitigating disease burden, proves particularly challenging given the nascent state of research specifically focused on this population.

Importantly, EOPC should not be regarded merely as prostate cancer occurring at a younger age. A growing body of evidence indicates fundamental differences from standard‐onset disease in terms of genetic susceptibility, molecular pathology, clinical presentation, and disease trajectory. These observations underscore the need to consider age‐specific mechanisms throughout the continuum of prostate cancer research and clinical care.

In this review, we synthesize current evidence on the epidemiology of EOPC, examining its risk factor profiles, biological characteristics, clinical manifestations, and potential management and early detection strategies. We begin by discussing the definitional challenges and epidemiological trends, followed by an analysis of genetic susceptibility and environmental risk factors. We then review the molecular pathology of EOPC, including its hallmark genomic alterations and tumor microenvironment (TME) features, before summarizing clinical outcomes and treatment responses. Finally, we critically appraise the limitations of existing evidence and propose a framework for future research to address the substantial knowledge gaps that remain.

## 2 Definition

Currently, no unified consensus exists regarding the specific definition of EOPC. In this article, EOPC is defined as prostate cancer diagnosed at the age of ≤55 years, with cases diagnosed after the age of 55 years correspondingly classified as standard‐onset prostate cancer (SOPC). Although demarcation using a single age threshold may initially appear arbitrary, establishing a clear delineation is imperative for the comprehensive characterization and synthesis of the clinical, pathological, and molecular attributes associated with EOPC. The rationale for selecting 55 years as the delineating threshold is threefold, drawing from epidemiological trends, existing literature, and molecular evidence.

First, in oncological epidemiology, the prevailing definition of early‐onset cancers typically includes those diagnosed before the age of 50 years, such as colorectal, breast, esophageal, endometrial, and liver cancers [7, 8, 9, 10]. However, early‐onset cancers are not a monolithic entity; their characteristics exhibit substantial heterogeneity depending on the organ of origin. Meanwhile, multiomics studies have revealed significant differences in the aging trajectories of different organs in both humans and mice [11, 12, 13]. Therefore, defining all early‐onset cancers with a uniform age threshold may lack precision. While the mean age at diagnosis for prostate cancer has decreased from 72 years in 1986 to 67 years in 2009 [5], it remains later than that of the aforementioned malignancies, which have historically been categorized as “geriatric cancers.” Consequently, if a specific age limit must be defined for EOPC, the age criterion should be correspondingly adjusted to reflect this clinical nuance.

**FIGURE 1:**
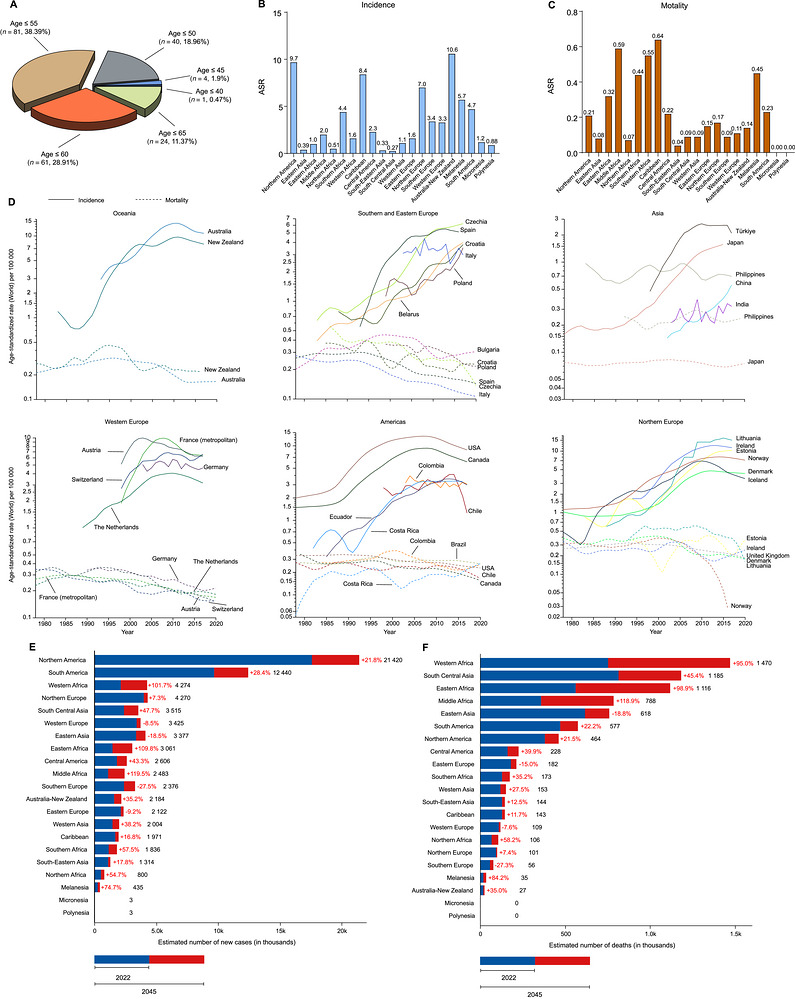
Global epidemiology, regional trends, and future projections of early‐onset prostate cancer incidence and mortality. (A) Definitions of early‐onset prostate cancer in contemporary literature; (B) global distribution of early‐onset prostate cancer incidence (2022); (C) global distribution of early‐onset prostate cancer mortality (2022); (D) temporal trends in incidence (solid line) and mortality (dashed line) across geographic regions; (E) projected incidence changes by region (2045); (F) projected mortality changes by region (2045).

Second, the age thresholds employed in published studies on EOPC remain considerably heterogeneous, with the literature citing a range from 40 to 65 years (Figure 1A). Analysis of the existing literature reveals that approximately 60% of studies categorize EOPC as occurring in individuals under 55 years of age. In light of the typically later onset of prostate cancer and informed by prior comprehensive reviews [5, 14], we propose defining EOPC as diagnosis before the age of 55 years. The current lack of consensus undermines the effective investigation and synthesis of the clinical, pathological, and molecular characteristics specific to EOPC, often resulting in inconclusive findings.

Furthermore, current whole‐genome sequencing, mRNA sequencing, single‐cell transcriptomic, and spatial transcriptomic (ST) studies that stratify prostate cancer using a 55‐year age cutoff have consistently demonstrated marked disparities between the two age‐defined cohorts in terms of tumor origin, molecular pathology, and therapeutic sensitivity (to be expounded in the *Molecular Pathology* section below) [15, 16].

As noted, prostate cancer is considered a geriatric malignancy, and advancing age constitutes a robust risk factor (with prostate cancer incidence rising significantly with increasing age); in other words, ageing represents the predominant risk factor for prostate cancer [4]. Ageing and carcinogenesis share numerous mechanistic pathways (e.g., accumulation of DNA damage, telomere attrition) [17]. Concurrently, profound shifts in population‐level exposures over recent decades have contributed to accelerated biological ageing across populations [18], and these exposures are also postulated as potential risk factors for early‐onset tumors [17]. Chronological age fails to capture interindividual variations in health status and functional capacity, hence the proposal of biological age to accurately characterize an individual's ageing state [19]. Some perspectives posit that early‐onset tumors may represent paradigmatic manifestations of accelerated ageing [20]. Should this hypothesis hold, future frameworks could establish tumor classification based on concrete biological age thresholds, thereby enabling more precise exploration of early‐onset tumor pathogenesis, biological and clinical characteristics, and preventive interventions.

We contend that elucidating the relationship between biological age and prostate cancer incidence, alongside defining EOPC according to biological age, warrants exploration. First, an individual's biological ageing results from the interplay of genetic background and environmental factors, a paradigm mirroring current understanding of tumorigenesis. Research by our group and others indicates that inherent high‐risk genetic mutations or elevated early‐life/chronic exposome burden accelerated biological ageing [21, 22, 23], aligning with the predominant hypothesis for early‐onset tumorigenesis [24]. Second, evidence from our team based on US, UK, and Chinese populations also demonstrates a significant association between accelerated biological ageing and serum testosterone levels [25]. Concurrently, preliminary evidence indicates that accelerated biological ageing is significantly associated with prostate cancer risk, particularly among individuals with a chronological age under 65 years [26]. Nevertheless, both fields remain in nascent stages of investigation.

## 3 Epidemiology

We analyzed global variations in EOPC incidence and mortality across 185 countries and territories, utilizing 2022 estimates from the International Agency for Research on Cancer's Global Cancer Observatory (GLOBOCAN). Globally, 61,471 new EOPC cases occurred in 2022. EOPC incidence showed substantial geographical heterogeneity, mirroring regional disparities in overall prostate cancer incidence [2]. The highest rates occurred in Australia–New Zealand (age‐standardized rate [ASR] 10.6 per 100,000), Northern America (ASR 9.7), the Caribbean (ASR 8.4), Northern Europe (ASR 7.0), and Melanesia (ASR 5.7) (Figure 1B), regions also reporting high SOPC incidence. Rates were lowest in Africa and Asia. The incidence in Australia–New Zealand exceeded that in South Central Asia by 39‐fold. For SOPC, Northern Europe's incidence was approximately 12 times higher than South Central Asia's.

EOPC caused an estimated 5381 deaths worldwide in 2022. Similar to SOPC, EOPC mortality showed minimal correlation with incidence across most regions, with the Caribbean being a notable exception (Figure 1C). Highest EOPC mortality rates occurred in the Caribbean, Central Africa, West Africa, Melanesia, and Southern Africa (Figure 1C). Mortality in the Americas and Europe exceeded rates in Asia. Regional mortality disparities for EOPC broadly aligned with those for SOPC.

Temporal trends in EOPC incidence and mortality were analyzed (Figure 1D). The Americas, particularly the United States and Canada, experienced a marked incidence rise during the 1990s, peaking in the 2000s before gradually declining, a pattern resembling SOPC trends but delayed by approximately 5 years. Oceania and Northern Europe also saw sharp incidence increases from the 1990s, continuing into the 2010s before stabilizing or declining slowly. Nordic SOPC trends paralleled EOPC. Conversely, Australia and New Zealand showed divergent SOPC patterns: New Zealand's SOPC incidence rose rapidly from the 1990s, peaking in the 2000s (a decade before EOPC peaked), then declined, similar to North America; Australia maintained high but fluctuating SOPC rates. Western Europe demonstrated largely uniform EOPC and SOPC trends, peaking around 2005 before declining (Figure 1D).

Asian countries exhibited distinct temporal patterns (Figure 1D). China experienced rapid continuous increases in both EOPC and SOPC incidence since the 2000s. Türkiye saw a sharp SOPC rise from ∼1995, peaking around 2005 then declining; EOPC peaked 5 years later and remained elevated. Japan's SOPC incidence rose rapidly from the 1980s, with EOPC increasing a decade later; both entered slow growth phases around the 2010s. The Philippines showed persistent long‐term fluctuations in both EOPC and SOPC incidence. India experienced continuous rapid SOPC increases alongside fluctuating EOPC rates. South Korea's EOPC and SOPC trends were broadly consistent, stabilizing after rapid growth between 1995 and 2010 (Figure 1D), mirroring Oceania, Northern Europe, and Western Europe.

EOPC mortality gradually declined in Oceania, Western, Southern, and Eastern Europe since the 2000s (Figure 1D). SOPC mortality decreased steadily in Oceania, Western, and Southern Europe from ∼1995, while remaining stable or slowly increasing in Eastern Europe. EOPC mortality remained relatively stable in the Americas, Asia, and Northern Europe (excluding Denmark), with Denmark showing a rapid decline post‐2005 (Figure 1D). SOPC mortality trends differed markedly: rates declined steadily from ∼1995 in North America, followed by Northern Europe (∼5 years later) and gradual declines in Latin America (∼10 years later). In Asia, SOPC mortality rose until stabilizing at higher levels after 1995.

Projections indicate EOPC incidence will rise 24.5% by 2045 (from 61,471 to 76,509 cases), while mortality will increase 50.0% (from 5381 to 8070 deaths). New case growth will be most pronounced in North America, South America, West Africa, and Northern Europe; mortality increases will concentrate primarily in Africa and Asia (Figure 1E,F).

Regional disparities in EOPC burden are strongly influenced by economic conditions, racial demographics, and industrial development. In high‐income countries (e.g., United States, Canada, Western/Northern Europe, Japan), prostate‐specific antigen (PSA) screening implementation facilitated early detection, initially driving rapid EOPC incidence increases. Subsequent comprehensive screening contributed to recent incidence stabilization or decline. Access to standardized effective treatment in these settings has reduced mortality.

Conversely, Black men exhibit elevated EOPC risk and potential aggressiveness. Many reside in low‐to‐middle‐income countries or face financial constraints, where PSA screening is limited and healthcare access is inadequate. Consequently, while African EOPC incidence remains relatively low, mortality is disproportionately high. Most Asian countries face economic barriers to comprehensive prostate cancer management, albeit marginally less severe than Africa, resulting in significant ongoing EOPC control challenges.

Race, diet, lifestyle, economic status, and health policy are all critical factors influencing prostate cancer incidence and mortality [2]. Within Asia, countries exhibit marked heterogeneity in economic development levels, ethnicity, living habits, and religious beliefs, leading to substantial disparities in EOPC incidence and mortality across the region [27, 28]. When broadly categorizing Asian countries into developing nations (e.g., China, the Philippines, India) and developed nations (e.g., Japan) based on economic status, a distinct pattern emerges: developed countries in Asia demonstrate overall trends in EOPC incidence and mortality that broadly mirror those observed in Western Europe and the United States. In contrast, developing countries display markedly different trajectories, likely attributable to disparities in economic development that shape national policies on PSA screening and its resultant uptake. In China, economic progress has facilitated wider access to PSA screening, which, coupled with the country's large population base, is contributing to rising EOPC incidence and portends a substantial future disease burden. Concurrently, socioeconomic status significantly influences access to healthcare services. A study by the Asian Urological Association investigating heterogeneous incidence rates across Asian countries revealed a significant positive correlation between per capita GDP and 5‐year prostate cancer survival rates in developing Asian nations, whereas no such association was observed in developed countries [27]. This likely reflects that per capita GDP in developed nations has reached a threshold sufficient to ensure timely access to medical care for prostate cancer patients. Furthermore, the genetic landscape of prostate cancer in Asian populations differs markedly from that in Caucasian populations [29], characterized by a low prevalence of the ERG oncoprotein, a low rate of PTEN loss, and high frequencies of CHD1 enrichment and FOXA1 alterations in East Asian populations. Collectively, these factors, economic status, screening access, and genetic background, contribute to the substantial disparities in EOPC incidence and mortality between these geographical regions.

Furthermore, industrialization in low‐and‐middle‐income countries increases exposure to industrial carcinogens, air pollutants, and Western dietary and lifestyle patterns, potentially elevating EOPC risk.

## 4 Factors Contributing to EOPC

Tumorigenesis is widely understood to result from the intricate interplay between endogenous and exogenous determinants [30]. Endogenous factors encompass hereditary mutations, familial susceptibility, the accrual of mutations during cellular turnover, and complex interactions among diverse cell types within the tissue microenvironment. Simultaneously, exogenous carcinogenic agents exert pressure on cells, instigating a cascade of microenvironmental and genetic modifications that collectively drive the neoplastic transformation of normal cells. This process culminates in tumor development.

Analyzing the causes of early‐onset tumors involves determining why the incidence of a specific tumor occurs significantly earlier than the typical age of onset (excluding effects attributable to population‐wide screening). This essentially involves an enhancement of the internal and/or external factors driving malignant transformation in normal cells, resulting in either an earlier initiation of malignant transformation and/or an accelerated transformation rate [24]. These states correspond to potential biological processes underlying early‐onset tumor development, for which corresponding preventive and therapeutic measures are fundamentally different. Concurrently, potential causative factors for early‐onset tumors can be summarized as: (1) presence of tumor‐driving germline mutations (genetic susceptibility), (2) chronic early‐life exposure to risk factors promoting malignant transformation in normal tissue cells, and (3) sustained exposure or temporally intensified exposure to tumor‐promoting risk factors.

**FIGURE 2:**
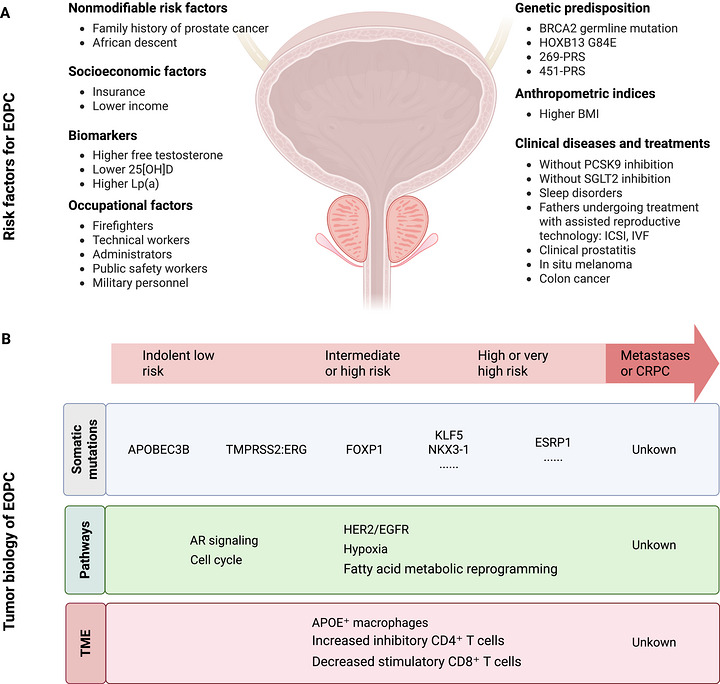
Risk factors and tumor biology of early‐onset prostate cancer. (A) Summary of established risk factors. Factors influencing EOPC risk include nonmodifiable factors such as genetic susceptibility, family history, and ethnicity, as well as modifiable factors such as socioeconomic status, biomarkers, clinical diseases, and occupation. (B) Molecular mechanisms underlying early‐onset disease pathogenesis. The development of EOPC is currently characterized by an AR‐centric model. Somatic mutations arising on a background of APOBEC3B mutagenesis and TMPRSS2:ERG fusion, together with corresponding alterations in tumor‐associated signaling pathways and the TME, collectively drive the initiation and progression of EOPC. *Abbreviations*: EOPC, early‐onset prostate cancer; TME, tumor microenvironment; CRPC, castration‐resistant prostate cancer.

Therefore, the etiology of EOPC may be attributed to the pronounced impact of intrinsic oncogenic factors, an increased burden of extrinsic carcinogens, or a synergistic effect of both. The causative factors of EOPC can likewise be discussed and explored within these frameworks. We will subsequently discuss potential differences between EOPC and SOPC pertaining to intrinsic factors, extrinsic factors, and tumor biology to achieve precise prevention and management (Figure 2).

### 4.1 Germline Genetic Pathogenic Variants

Prostate cancer is regarded as a malignancy with a strong association with a family history of cancer, displaying a familial predisposition that exceeds that of all other cancers [4]. Individuals with a family history or hereditary susceptibility are generally considered to have an augmented risk of developing prostate cancer compared with the general populace, often presenting at a relatively young age, specifically under 55 years at diagnosis. Epidemiological studies suggest that approximately 9% of patients diagnosed with prostate cancer report a familial history of the disease [31, 32], with first‐degree relatives (FDRs) diagnosed with prostate cancer conferring a twofold increase in the risk of developing the malignancy [33, 34]. While it is widely acknowledged that EOPC typically exhibits a higher degree of hereditary correlation than standard‐onset cases, there is currently a paucity of robust population‐based cohort studies aimed at elucidating the germline mutation characteristics specific to EOPC.

#### 4.1.1 Homologous Recombination Repair

Germline mutations in DNA damage repair (DDR) genes (e.g., BRCA1, BRCA2, ATM, ATR, NBS1, CHEK2, RAD51D, PALB2) or mismatch repair (MMR) genes (e.g., MSH2, MSH6, PMS2) are established risk factors for prostate cancer, frequently manifesting at younger ages. However, the relationship between these mutations and EOPC is complex. Distinct mutational landscapes in DDR/MMR genes differentiate EOPC from SOPC, precluding direct extrapolation of risk profiles between these cohorts. Current research focuses predominantly on the impact of BRCA1/2 germline mutations on EOPC development and is almost exclusively retrospective.

Overall, the population prevalence of BRCA1/2 is 0.7% (exhibiting ethnic variation, ranging from 0.2% in the Dominican Republic to 2.0% in Ashkenazi Jewish populations) [35]. In general localized prostate cancer, the prevalence of BRCA1/2 germline mutations is approximately 2–6%, increasing with tumor grade (aggressive or metastatic disease: 4–13%) [3, 36]. Although carriers of BRCA2 germline mutations develop prostate cancer at a younger age than noncarriers and show a significant association with aggressive prostate cancer, the BRCA2 germline mutation rate in EOPC specifically is not significantly higher than in SOPC. Current evidence suggests that BRCA2 germline mutations may interact more with the inherent susceptibility of individuals with a family history of prostate cancer to advance the age of onset, rather than acting in isolation. Similarly, BRCA2 germline mutations are also significantly associated with an increased risk of aggressive EOPC.

This complexity is illustrated by Leon et al. [37], who analyzed 325 patients stratified by: (1) EOPC, (2) familial prostate/uterine/ovarian malignancies, and (3) metastatic prostate cancer. Mutation prevalence was 5.9, 10.9, and 6.9% across cohorts. A Bayesian model quantified BRCA2 mutation contributions across these states at 22, 51, and 27% respectively. These data demonstrate a stronger association with familial inheritance than with early onset. Moreover, BRCA2 mutations showed greater contribution to high‐risk disease (71.4% of carriers had aggressive cancer: PSA > 20 ng/mL, Gleason score [GS] ≥ 7, T3 staging, or metastases) than to age of onset.

Strengthening this evidence, Maia et al. [38, 39] studied 460 Northern Portuguese patients with EOPC or familial prostate cancer. Crucially, BRCA2 mutations were absent in EOPC cases, occurring exclusively in familial cases. Homologous recombination repair (HRR) gene profiles diverged significantly between groups, with elevated ATM, BRCA2, CHEK2, and PALB2 mutations in patients >56 years. Similarly, Coelho et al. [40] reported higher HRR mutation rates in Brazilian SOPC versus EOPC (64 vs. 25%), with BRCA2 absent in EOPC but reaching 31% (11 out of 35) in SOPC. ATR mutations occurred in 33% (three out of nine) of EOPC cases, while BRCA2 and PALB2 were exclusive to SOPC. Consistent with these patterns, Tischkowitz et al. [41] found no significant PALB2–EOPC association in 95 Canadian EOPC patients. Given PALB2's role as a BRCA2 cofactor, this absence challenges direct links between BRCA2 mutations and early onset.

Expanding on age‐risk dynamics, Nyberg et al. [42] followed 376 BRCA1 and 447 BRCA2 carriers (United Kingdom/Ireland). Over mean follow‐ups of 5.9 and 5.3 years, prostate cancer incidence yielded standardized incidence ratios (SIR) of 2.35 (95% CI 1.43–3.88; 16 cases) and 4.45 (95% CI 2.99–6.61; 26 cases), respectively. BRCA1 carrier incidence approximated population levels across age strata, while BRCA2 risk escalated with advancing age. Familial history further increased risk among BRCA2 carriers (HR 1.68; 95% CI 0.99–2.85).

Building on evidence that MMR and DDR gene mutations may increase prostate cancer risk, the IMPACT study explored PSA‐based screening targeting germline MSH2, MSH6, and BRCA2 mutations [43, 44, 45]. This approach demonstrated value in detecting clinically significant prostate cancer in carriers, though without age‐stratified analysis. Despite significantly younger diagnosis age in BRCA2 carriers versus noncarriers (61 vs. 64 years; *p* = 0.044), both groups were diagnosed >55 years, undermining evidence for BRCA2 as a driver of early‐onset disease.

Supporting the risk stratification, a single‐arm imaging‐based screening study of BRCA mutation carriers revealed significantly higher rates of high‐grade tumors (Grade Group, GG ≥ 2) in BRCA2 carriers (71 vs. 23% in BRCA1 carriers; *p* = 0.049) [46]. Though limited sample size precluded age‐stratified comparison between BRCA1 and BRCA2 carriers, detection rates showed clear age‐dependence: (1) 3.7% in 40–50 year‐olds (exclusively GG1); (2) 12% in ≥50 year‐olds (encompassing all GG ≥2 tumors).

Collectively, these findings substantiate two key conclusions: first, BRCA2 mutations correlate more strongly with familial inheritance than with age of onset. Second, they demonstrate a consistent association with high‐risk disease and adverse outcomes. Given this evidence, a primary association between HRR gene mutations and EOPC remains unsupported; instead, their linkage to hereditary prostate cancer represents a more substantiated etiological model.

#### 4.1.2 HOXB13

The HOXB13 gene (17q21–22) encodes transcription factor homeobox B13. Ewing et al. [47] identified a codon 84 missense mutation (GGA→GAA; Gly84Glu, rs138213197) through exome sequencing of 94 familial prostate cancer cases. Validation in 5083 prostate cancer patients and 1401 controls of European ancestry revealed significantly higher HOXB13 G84E germline mutation prevalence in cases versus controls (1.4 vs. 0.1%; OR 20.1, 95% CI 3.5–803.3). Within cases, carrier rates were elevated in EOPC and familial prostate cancer versus SOPC and sporadic cases. The highest rate occurred in familial EOPC (3.1%) versus the lowest in sporadic SOPC (0.6%). Both early onset and family history comparably influenced carrier rates. Karlsson et al. [48] corroborated this age association in Swiss populations (4903 cases/4589 controls), showing progressive carrier rate decline with advancing age.

Contrastingly, Laitinen et al. [49] reported stronger familial than age effects in Finnish cohorts (4000 cases/5000 controls). Carrier rates were higher in cases (3.5%) than controls (0.5%), with EOPC showing elevated rates versus SOPC (6.25 vs. 3.40%; OR 2.0, 95% CI 1.3–3.0). Familial history substantially increased rates (8.4% familial vs. 2.2% sporadic; OR 4.2, 95% CI 2.6–6.6). Notably, all the three European studies demonstrated no association between G84E status and GSs.

In American cohorts, divergent patterns emerged. Witte et al. [50] (2664 cases/1189 controls; 80% Caucasian) reported higher carrier rates in cases (OR 4.79, *p* = 0.01). Family history showed negligible effect (1.46% familial vs. 1.10% sporadic), whereas EOPC rates exceeded SOPC (2.64% vs. 0.96%). Meta‐analysis of European and US data confirmed stronger age than familial effects (EOPC vs. control OR 8.41, 95% CI 5.27–13.76; familial vs. control OR 7.19, 95% CI 4.55–11.67) [47, 51, 52], also with no GS association. Schroeck et al. [53] (387 cases/597 controls) observed minimal carrier rates (overall 0.42%, EOPC 0.44%), identifying no carriers among 301 familial cases, suggesting population‐specific rarity.

Collectively, the G84E mutation occurs predominantly in populations of European ancestry and demonstrates a strong association with EOPC incidence within this demographic. While familial history elevates carrier rates, the mutation exhibits no correlation with disease aggressiveness. Current evidence suggests HOXB13 G84E may serve as a potential biomarker for EOPC screening in men of European descent. However, as this mutation shows no significant association with prostate cancer aggressiveness and remains relatively uncommon in the population, screening strategies based solely on G84E detection risk substantial overdiagnosis of indolent disease and subsequent overtreatment. Future research is warranted to determine how this genetic variant might be optimally utilized to improve early detection strategies for prostate cancer.

Notably, African populations show distinct molecular epidemiology. No G84E carriers were identified in African studies. Instead, Na et al. [54] reported elevated HOXB13 c.853delT (X285K) prevalence in 1048 African‐American (AA) surgical patients with clinically significant disease (Gleason ≥ 7: 1.2% vs. Gleason < 7: 0%; OR inf, 95% CI 1.05‐inf, *p* = 0.028). Rates were higher in patients <50 years versus ≥50 years (2.4 vs. 0.5%; OR 5.25, 95% CI 1.00–28.52, *p* = 0.03).

#### 4.1.3 Other Germline Mutations

Recent genomic sequencing studies have elucidated novel germline mutations potentially implicated in the pathogenesis of EOPC. However, these findings remain constrained by limited sample sizes and an absence of independent validation. Cardoso et al. [55] performed whole‐exome sequencing on 96 individuals with EOPC or hereditary prostate cancer from 45 distinct families, identifying a significantly elevated incidence of pathogenic mutations in the PRUNE2 gene. Subsequent validation in a larger cohort of 462 patients from prostate cancer pedigrees confirmed a mutation prevalence of 2.8%, suggesting PRUNE2 constitutes an additional gene associated with early‐onset or familial disease. Building on this, Lange et al. [56, 57] conducted a comprehensive analysis of 931 unrelated EOPC patients and 1126 controls of European descent from the University of Michigan Prostate Cancer Genetics Project (UM‐PCGP). Their findings demonstrate a robust correlation between a high burden of risk alleles and EOPC. Collectively, these studies underscore the intricate genetic architecture of EOPC, revealing numerous pivotal genes yet to be identified. Notably, 62% of participants in Cardoso's investigation reported a familial history of prostate cancer.

To further elucidate causal relationships between germline variants and disease outcomes, Mendelian randomization (MR) approaches have been employed. Desai et al. [58] conducted an MR analysis within the PRACTICAL consortium, encompassing an overall prostate cancer cohort (85,554 cases; 91,972 controls), an aggressive prostate cancer cohort (15,167 cases; 58,308 controls; defined as metastatic disease, GS ≥ 8, PSA > 100 ng/mL, or prostate cancer death), and an EOPC cohort (6988 cases; 44,256 controls). This analysis identified 20 independent proteins significantly associated with prostate cancer risk: 14 with overall risk (eight specific), seven with aggressive disease (three specific), and eight with early‐onset disease (two specific). Critically, these results highlight substantial differences in the germline mutation landscape between EOPC and SOPC. The two proteins exclusively associated with EOPC risk were PYY (OR 1.87, 95% CI 1.43–2.44) and SERPINA3 (OR 2.08, 95% CI 1.51–2.88). Additionally, PPA2 demonstrated significant associations with heightened risk for both aggressive (OR 2.13, 95% CI 1.54–2.93, PP4 = 0.99) and EOPC (OR 2.70, 95% CI 1.71–4.27, PP4 = 0.93), while also showing association with overall risk (OR 1.84, 95% CI 1.52–2.22, PP4 = 0.01; PP4 > 0.70 indicates significant association). Importantly, no proteins were significantly associated with high risk for both overall and aggressive prostate cancer concurrently.

#### 4.1.4 Polygenic Risk

In addition to the association between individual gene germline mutations and the risk of EOPC, polygenic risk scores (PRS) may provide a more comprehensive assessment of the influence of these mutations on disease susceptibility.

A PRS based on 147 SNPs (147‐PRS), derived from an analysis of 46,939 prostate cancer cases and 27,910 controls of European ancestry, demonstrated significant associations with both overall and aggressive prostate cancer risk [59, 60]. This analysis did not evaluate age‐specific risk, and the SNP selection process excluded the sole variant uniquely associated with EOPC susceptibility. Building directly upon these findings, the BARCODE1 study [61, 62] enrolled men aged 55–69 years and established that screening with MRI and subsequent biopsy for men in the top 90th percentile of a derived 130‐SNP score (refined by excluding 17 SNPs failing quality control from the original 147‐PRS) significantly increased the detection of clinically significant prostate cancer. These findings indicate promising implications for PRS‐based targeted screening strategies. However, the development and validation of this PRS approach (referring to the 147‐PRS and its derivative in BARCODE1) entirely excluded individuals with EOPC and were restricted to populations of European ancestry. Subsequent research has developed multiethnic PRSs (269‐PRS and 451‐PRS), which potentially hold greater promise and specificity for EOPC cohorts, particularly the 451‐PRS.

Conti et al. [63, 64] performed a large‐scale GWAS meta‐analysis of 107,247 prostate cancer cases and 127,006 controls across four ancestries: European (85,554 cases; 91,972 controls), African (10,368; 10,986), Hispanic (2714; 5239), and East Asian (8611; 18,809). Their analysis identified 86 novel variants beyond 183 established loci, forming a 269‐variant PRS (269‐PRS). Elevated 269‐PRS consistently predicted higher prostate cancer risk across all ancestries, with validation in independent cohorts (UK Biobank [European] and California‐Uganda study [African]).

Critically, both family history and age at diagnosis modified PRS‐associated risk. Patients in the highest 269‐PRS decile demonstrated twice the prostate cancer risk in FDRs compared with the lowest decile, indicating significant, though less substantial than age, familial influence on germline risk. Among the 269 variants, 188 exhibited stronger effects in EOPC versus SOPC, with 31 showing statistically significant differential effects. This indicates a disproportionately greater germline contribution to EOPC pathogenesis than to standard‐onset disease, revealing biological complexity beyond simple familial‐early‐onset correspondence.

Further analysis of 31,925 cases and 490,507 controls [65] (European: 22,049/414,249; African: 8794/55,657; Hispanic: 1082/20,601) examined age interactions. Compared with men with average 269‐PRS (40–60%), those in the highest decile (90–100%) had prostate cancer OR of 3.8 (95% CI 3.62–3.96) for European, 2.8 (2.59–3.03) for African, and 3.2 (2.64–3.92) for Hispanic ancestry. While 269‐PRS did not distinguish aggressive disease, ORs declined progressively with age: European men in the highest decile had ORs of 7.11 (≤55 years), 4.26 (55–60 years), and 2.79 (>70 years). These men reached 5% absolute risk ∼10 years earlier than average‐PRS counterparts.

Concurrently, a European‐ancestry study (UK Biobank/PLCO/TCGA) confirmed greater 269‐PRS impact for men ≤55 years versus older patients, irrespective of paternal/fraternal history [66]. This study further developed an EOPC‐specific 45‐variant PRS (EOPC‐PRS). Increasing EOPC‐PRS significantly elevated EOPC risk (HR 4.70, 95% CI 3.98–5.54) but not SOPC risk (HR 0.98, 0.96–1.01). Notably, high EOPC‐PRS associated strongly with low‐risk pathology (GS < 7, T ≤ T2, N0) but inversely with adverse features (higher GS, advanced T‐stage, nodal involvement). Consequently, neither 269‐PRS nor EOPC‐PRS demonstrates sufficient clinical utility for early‐onset screening: they lack specificity for clinically significant EOPC, potentially increase overdiagnosis, and fail to improve upon PSA‐based screening paradigms.

Wang et al. developed a 451‐SNP PRS (451‐PRS) using multiancestry GWAS data (European: 122,188 cases/604,640 controls; African: 19,391/61,608; East Asian: 10,809/95,790; Hispanic: 3931/26,405) [67]. This study performed analogous analyses of prostate cancer risk across ancestry, age, and family history strata as conducted for the aforementioned 269‐PRS. For individuals within the top 90–100% of the 451‐PRS distribution, this score demonstrated stronger associations with overall prostate cancer, EOPC, and SOPC than a 267‐PRS across all ancestries. The 451‐PRS also exhibited consistently stronger associations with aggressive versus nonaggressive disease, though without age stratification. Subsequently, Plym et al., utilizing data from men aged 53–64.7 years in Swedish and US cohorts (Malmö Diet and Cancer Study and Health Professionals Follow‐Up Study; 1991–2019 follow‐up), stratified prostate cancer patients into high (above‐average 451‐PRS or family history) versus low genetic risk groups [68]. High‐risk patients had significantly increased hazards for both early death (<75 years; HR 3.26, 95% CI 1.82–5.84) and late death (≥75 years; HR 2.26, 95% CI 1.70–3.01), further strengthening evidence linking elevated 451‐PRS with prostate cancer aggressiveness. These results suggest that a 451‐PRS‐based targeted screening strategy may yield promising outcomes.

These findings validate the multiancestry PRS as a robust tool for prostate cancer risk stratification across diverse populations. Further clinical studies are warranted to investigate the potential application of PRS for risk‐adapted early detection of prostate cancer.

Presently, research specifically focused on germline mutations in EOPC remains limited. Most evidence derives from subgroup analyses of earlier studies, predominantly involving cohorts combining sporadic EOPC cases with those exhibiting familial or hereditary patterns. Although hereditary prostate cancer is acknowledged to present earlier, the clinical relevance of sporadic EOPC warrants consideration, a point addressed subsequently. Consequently, current evidence on germline mutations in EOPC may be most applicable to patients with a documented family history. We suggest future investigations should explicitly delineate sporadic, familial, and hereditary prostate cancer cases, systematically assessing and validating the prevalence of pertinent germline mutations and their correlation with disease risk. Such stratification is essential for establishing more precise and targeted screening strategies.

Notably, these germline alterations appear to play a more prominent role in younger patients than in those with standard‐onset disease, suggesting that inherited susceptibility may be a key driver of early disease development. Furthermore, these germline alterations, including mutations in HRR genes, HOXB13, and polygenic risk variants, underscore the critical role of inherited susceptibility in the development of EOPC. However, genetic predisposition alone does not fully account for disease occurrence. The penetrance and clinical manifestation of these variants are likely to be modulated by additional factors.

In this context, a range of nongenetic risk factors may interact with underlying genetic susceptibility to shape disease risk and presentation, as discussed below.

### 4.2 Risk Factors

In addition to inherited genetic susceptibility, a range of nonmodifiable and modifiable factors have been implicated in the risk of EOPC. Importantly, these factors do not act in isolation but may interact with underlying germline variants to influence both disease initiation and progression. Understanding this interplay is essential for a more comprehensive characterization of disease risk. Importantly, the relative contribution of these risk factors may differ in younger patients, in whom traditional age‐related risk accumulation is less pronounced and genetic predisposition may play a more dominant role.

#### 4.2.1 Nonmodifiable Risk Factors

##### 4.2.1.1 Family History

Family history is unequivocally recognized as an independent risk factor for prostate carcinogenesis, with particular predilection for aggressive phenotypes [69, 70]. It is widely acknowledged that the increased prostate cancer risk associated with family history is largely attributable to genetic susceptibility; however, shared environmental risk factors within families could also contribute to familial clustering. Key family history determinants include: (1) the number of affected relatives, (2) the degree of kinship to the proband, (3) the age at diagnosis of affected relatives, and (4) the pathological grade of affected relatives.

A landmark meta‐analysis [71] synthesizing data from 33 observational studies established that men with affected FDRs exhibit a 2.48‐fold elevated disease risk, escalating to 4.39‐fold with ≥2 affected FDRs. Population‐based cohort studies from the United Kingdom [72], China [73], and South Korea [74] corroborate this dose–response relationship for EOPC.

Emerging evidence delineates three distinctive patterns of familial risk stratification specific to EOPC.

First, disproportionate risk amplification. Analysis of the Utah Population Database (*n* = 619,630) [75] identifies differential risk gradients: familial history confers 1.81‐fold, 1.70‐fold, and 1.79‐fold increased risks for overall prostate cancer, lethal prostate cancer, and clinically significant prostate cancer, respectively, whereas EOPC risk escalates to 3.38‐fold. Men with three or more affected FDRs demonstrate an 8.72‐fold EOPC risk elevation, exceeding corresponding increases for overall (2.86‐fold) and lethal (3.02‐fold) disease.

Second, temporal concordance in diagnostic age. Multigenerational analysis of the Swedish Family‐Cancer Database [76] identifies temporal concordance: offspring of fathers diagnosed at younger ages experience 7.8‐fold increased EOPC risk versus 3.8‐fold for SOPC. The EOPC risk gradient attenuates more sharply with advancing paternal diagnosis age (4.59‐fold difference between earliest and latest paternal diagnosis quartiles) compared with SOPC (2.71‐fold difference).

Third, paradoxical histological protection. Pathological analysis of 498 radical prostatectomy (RP) specimens revealed reduced odds of high‐grade EOPC among men with affected FDRs (adjusted odds ratio [aOR] 0.50, 95% CI 0.29–0.87) or paternal history (aOR 0.42, 95% CI 0.23–0.76). These observations warrant validation in prospective cohorts given the methodological constraints of retrospective design and limited sample size [77].

This association may be particularly pronounced in early‐onset disease, further supporting a stronger genetic contribution in younger individuals.

##### 4.2.1.2 Race

Race, while fundamentally a social construct historically rooted in hierarchical assertions of group superiority, intersects with biological and socioeconomic dimensions [78]. Ancestral genetic variation, cultural practices, lifestyle factors, and systemic healthcare inequities collectively drive differential disease risks and outcomes across racial groups [78, 79]. Prostate cancer exhibits the most pronounced racial disparities in incidence and mortality among malignancies [80].

Current epidemiology indicates that US Black/AA men experience a 1.7‐fold higher prostate cancer incidence and 2.1‐fold greater mortality versus White men [69]. In both the United States and United Kingdom, Black men face a 2–3‐fold increased risk of lethal prostate cancer diagnosis [81]. However, when matched for Gleason grade, clinical stage, and healthcare access, survival outcomes achieve parity between Black and White patients [80], underscoring race as a composite variable entangled with genetic predisposition, environmental exposures, and structural inequities [79]. Emerging evidence suggests that racial disparities in EOPC may arise from divergent tumor progression dynamics rather than initiation timing (shortened tumor age with unchanged initiation of tumorigenesis).

First, uniform initiation with accelerated progression in Black men. A systematic autopsy review (*n* = 58 studies) demonstrated comparable prevalence of indolent prostate cancer in Black and White males aged 20–29 years (Asian data sparse). Divergence emerged in the fourth decade: prevalence rates were 4% (Asian), 8% (White), and 35% (Black) [82]. By the age of 60 years, Black men reached 56% prevalence, a threshold attained by White and Asian men only in their 80s (49%) and 90s (50%). A US autopsy study (*n* = 1056; 1992–2001) corroborated this, revealing high‐grade prostatic intraepithelial neoplasia (HGPIN) commencing synchronously in Black and White males during their 20s. Black men exhibited abrupt HGPIN escalation by the age of 40 years—a surge delayed until the 70s in White males [83].

Second, inverse relationship between age and risk magnitude. SEER analyses (1995–2004) showed Black‐to‐White incidence rate ratios decreasing progressively from 4.91 (ages 40–49 years) to 3.08 (70–79 years) [83]. A UK cohort study of first‐generation immigrants (*n* = 2140; 1995–2001) confirmed this gradient: Black Caribbean (*p* = 0.002) and Black African (*p* = 0.02) men exhibited steeper disparities at younger ages (*p* < 0.001 overall) [84].

Finally, paradoxical survival advantage in EOPC. Analysis of 977,722 National Cancer Database cases (2004–2020) revealed comparable lethal disease rates between Black and White men with EOPC (aOR 0.98, 95% CI 0.91–1.06). Hispanic men, however, had elevated lethal risk (aOR 1.34, 95% CI 1.20–1.50). This parity contrasts with SOPC, where both Black and Hispanic men experience disproportionate mortality [85].

Such disparities may manifest differently in younger patients, potentially reflecting interactions between genetic susceptibility and environmental exposures at earlier stages of life.

#### 4.2.2 Modifiable Risk Factors

The etiology of EOPC remains incompletely defined. Current evidence regarding the association between exposure to specific risk factors and the development of early‐onset disease is both insufficient and methodologically limited, resulting in findings of low robustness. Critically, the majority of contemporary patients with EOPC were born after the mid‐20th century, a period characterized by profound societal industrialization, dramatic shifts in population lifestyles, and significant alterations in environmental exposures. Evidence suggests that exposures occurring in early life, compared with those encountered in adulthood, are associated with a considerably longer latency period [86]. This observation lends biological plausibility to the hypothesis that early‐life exposures may be a significant driver of the declining age at prostate cancer onset.

However, the current evidence base for the etiology of early‐onset, and indeed prostate cancer overall, relies predominantly on case–control study designs. These studies frequently suffer from critical limitations: the temporal sequence between putative risk factors and cancer development often remains unclear, and the precise timing of exposure is rarely well‐defined. Consequently, the identified associations often represent correlational observations rather than demonstrably causal risk factors. A particularly salient illustration of these limitations is the discordance observed between conclusions drawn from case–control studies and those derived from MR studies. MR analyses, which utilize germline genetic variants as instrumental variables to infer causal effects of modifiable exposures, often yield results inconsistent with traditional observational findings (these specific points of discordance will be elaborated upon in subsequent discussion). Furthermore, many studies focus on risk factors manifesting in adulthood, such as obesity, which may themselves be downstream consequences of earlier‐life exposures. It is plausible that these underlying early‐life factors constitute the true, fundamental drivers of risk.

In the absence of large‐scale prospective birth cohort studies specifically designed to investigate risk factors for EOPC, synthesizing insights from MR analyses alongside carefully interpreted retrospective data may offer a pragmatic approach for the preliminary identification of plausible risk factors. Therefore, notwithstanding the methodological caveats and uncertainties surrounding causality outlined above, we summarize below the currently purported risk factors (Figure 2A). This synthesis is presented to inform the design and prioritization of future research.

##### 4.2.2.1 Socioeconomic Factors

In a population‐based cohort study using the National Cancer Database (2004–2020), Smani et al. analyzed 977,722 prostate cancer patients [85]. Multivariable logistic regression revealed significant associations between insurance status and lethal presentation in EOPC. Compared with private insurance, all alternative statuses conferred elevated lethal disease risk: uninsured patients showed the highest risk (aOR 4.14, 95% CI 3.69–4.65), followed by Medicaid beneficiaries (aOR 3.39, 95% CI 3.10–3.72), unknown insurance status (aOR 1.80, 95% CI 1.42–2.29), and Medicare recipients (aOR 1.50, 95% CI 1.32–1.70). Notably, other government insurance demonstrated protection against lethal EOPC (aOR 0.64, 95% CI 0.50–0.82). These associations progressively attenuated with advancing diagnostic age in standard‐onset disease.

Socioeconomic analysis showed differential effects by income quartile: versus the highest income bracket (≥$74,063), lower income groups exhibited graded lethal risk increases for standard‐onset cases, whereas only the lowest income category (<$46,277) was associated with elevated risk in EOPC (aOR 1.15, 95% CI 1.02–1.30). High school graduation rates had comparable effects across both cohorts, with each 5% increase in nongraduation rates correlating with modest lethal risk elevation [85].

In younger populations, these socioeconomic factors may exert a more pronounced influence on access to early detection and timely diagnosis, thereby shaping disease presentation.

##### 4.2.2.2 Anthropometric Indices

(1) Body mass index

A meta‐analysis demonstrated an inverse association between elevated body mass index (BMI) (per 5 kg/m^2^ increment) and serum PSA levels in males (6% decrease), though no significant correlation was observed with overall prostate cancer incidence [87]. These findings align with recent MR studies [88, 89]. Age‐stratified analyses revealed divergent patterns: MR showed no significant association between BMI and EOPC risk [88], whereas a Korean population‐based cohort study (*n* = 5,370,614) demonstrated progressively stronger positive associations in men aged 40–65 years (*p* for trend <0.0001) and >65 years (*p* for trend <0.0001), with no association observed in those aged 20–39 years [90].

Notably, both MR and observational studies consistently reported null associations between BMI and aggressive prostate cancer risk [88, 91]. Conversely, a retrospective cohort analysis identified a dose–response relationship between elevated BMI (≥27.4 kg/m^2^) and high‐grade EOPC in men aged <50 years (aOR 4.17, 95% CI 1.32–13.17; *p* for trend = 0.02), though this association attenuated in the 50–55 year subgroup (aOR 1.24, 95% CI 0.87–1.77) [77]. This finding, while biologically plausible, remains provisional due to the study's significant limitations, particularly its retrospective design and modest sample size (*n* = 498), necessitating validation through prospective, adequately powered investigations.

(2) Lengths of fingers

A 16‐year prospective analysis of 6258 predominantly Australian‐born males within the Melbourne Collaborative Cohort Study investigated associations between the second‐to‐fourth digit ratio (2D:4D), a putative prenatal biomarker of androgen exposure, and prostate cancer risk [92]. No significant association was observed between 2D:4D and overall prostate cancer incidence (left hand: HR 1.00, 95% CI 0.93–1.08; right hand: HR 1.00, 95% CI 0.92–1.07). However, age‐stratified analyses suggested a potential protective effect of higher digit ratios (indicative of lower prenatal androgen activity) against early‐onset disease. Specifically, each standard deviation increase in 2D:4D at the age of 55 years was correlated with an HR of 0.80 (95% CI 0.65–1.10) for both hands, implying that elevated fetal androgen exposure may predispose to earlier carcinogenesis. Notably, inverse trends were observed for aggressive tumors (defined as GS > 7 or Stage IV malignancy), with left‐hand 2D:4D yielding an HR of 0.88 (95% CI 0.75–1.04) and right‐hand 2D:4D an HR of 0.94 (95% CI 0.79–1.13), though confidence intervals spanned unity. These findings support the developmental origins hypothesis in EOPC, suggesting that androgen‐mediated in utero programming may modulate disease phenotype and temporal onset.

##### 4.2.2.3 Biomarkers

(1) Serum testosterone

Integrated analyses of MR (UK Biobank: 194,500 men; PRACTICAL consortium: 79,000 prostate cancer cases) and observational serum biomarker data (Endogenous Hormones, Nutritional Biomarkers and Prostate Cancer Collaborative Group [EHNBPCCG]: 14,944 cases/36,752 controls) examined associations between total testosterone, free testosterone, and prostate cancer phenotypes [93]. Discordant findings emerged for total testosterone: genetically predicted higher levels showed a marginal inverse association with overall prostate cancer risk (per 1 SD increase: MR OR 0.96, 95% CI 0.94–0.99), whereas observational analyses demonstrated null effects (OR 0.97, 95% CI 0.89–1.07). Both methodologies indicated no significant association between total testosterone and EOPC.

Conversely, elevated free testosterone exhibited concordant positive associations with overall prostate cancer risk (MR OR 1.20, 95% CI 1.08–1.34; observational OR 1.03, 95% CI 1.01–1.05). Stratification by age and aggressiveness revealed heterogeneous patterns: neither approach identified significant associations between free testosterone and early‐onset or aggressive prostate cancer (GS > 7 or metastatic disease). However, age‐specific analysis of aggressive cancers demonstrated that genetically predicted free testosterone elevation selectively increased aggressive EOPC risk (OR 1.77, 95% CI 1.05–2.99 per 1 SD), with no effect on standard‐onset disease (OR 0.95, 95% CI 0.88–1.02). These findings indicate temporal‐phenotypic heterogeneity in androgen‐mediated prostate carcinogenesis.

A limitation is the predominantly European ancestry of analyzed cohorts, potentially restricting generalizability to other populations.

(2) Serum vitamin D

A 13‐year Finnish nested case–control study (19,000 men; 158 incident prostate cancer cases) investigated serum 25‐hydroxyvitamin D (25[OH]D) levels and prostate cancer risk [94]. Nonlocalized prostate cancer patients aged <52 years exhibited significantly lower serum 25(OH)D levels than controls, whereas no significant differences were observed in patients aged ≥52 years. Stratified analyses demonstrated that serum 25(OH)D levels <40 nmol/L were associated with a threefold increased risk of EOPC (age <52 years: aOR 3.0, 95% CI 1.5–5.9). No significant association was observed in men aged ≥52 years. These findings suggest that vitamin D deficiency (25[OH]D <40 nmol/L) specifically elevates EOPC risk, particularly for nonlocalized tumors. The study might indicate an age‐dependent biological role for vitamin D in prostate carcinogenesis, with more pronounced effects in younger populations.

(3) Circulating insulin‐like growth factors

Integrated analyses of individual participant data from 20 prospective cohorts (observational component: 17,009 prostate cancer cases, 37,243 controls) and MR (158,444 UK Biobank participants of European ancestry) examined associations between insulin‐like growth factors (IGFs), IGF‐binding proteins (IGFBPs), and prostate cancer risks [95]. Both methodologies demonstrated significant positive dose‐dependent associations between elevated IGF‐I levels and increased risks of overall and aggressive prostate cancer. Neither approach identified significant associations with early‐onset disease.

Age‐stratified observational analyses revealed that elevated IGF‐I correlated specifically with SOPC and aggressive standard‐onset subtypes, with no association observed for early‐onset counterparts. Analyses of IGF‐II and IGFBP1–3 (limited to observational data) indicated no significant relationships with aggressive or EOPC. Elevated IGF‐II and IGFBP3 were associated with increased overall prostate cancer risk, whereas IGFBP1 exhibited inverse associations with overall disease. These patterns suggest IGF‐II and IGFBPs predominantly influence standard‐onset carcinogenesis.

Collectively, serum IGFs and related proteins do not appear biologically relevant to EOPC pathogenesis but may modify standard‐onset disease progression.

(4) Glycometabolic parameters

A South Korean cohort (*n* = 5,370,614) found no significant association between blood glucose levels and prostate cancer in men aged 20–39 years or ≥65 years, though a marginal protective effect was observed at 40–64 years (adjusted hazard ratio [aHR] 0.966, 95% CI 0.937–0.996) [90]. Conversely, 10‐year follow‐up of the China Cardiometabolic and Cancer Cohort (4C; *n* = 57,779) detected no significant associations of serum HbA1c with overall, advanced (metastatic disease, GS ≥ 8, PSA > 100 ng/mL, or prostate cancer‐specific mortality [PCSM]), or EOPC risk [96]. These null findings were substantiated by subsequent UK Biobank analyses and MR studies (PRACTICAL and GAME‐ON/ELLIPSE Consortia) [96].

Collectively, current evidence suggests glycemic parameters and HbA1c levels do not substantially influence prostate cancer development in males.

(5) Lipid metabolic parameters

The relationship between serum lipid parameters and prostate cancer risk remains inconsistent across study methodologies. An umbrella review identified suggestive evidence linking elevated total cholesterol to high‐grade prostate cancer, while high‐density lipoprotein (HDL) and low‐density lipoprotein (LDL) showed no significant associations [89]. Subsequent age‐stratified analyses in a Korean cohort (*n* = 5,370,614) revealed progressively stronger associations with increasing age: no association was observed for low HDL (aHR 0.696, 95% CI 0.406–1.192) or elevated triglycerides (aHR 1.196, 95% CI 0.848–1.688) in men aged 20–39 years. However, marginal risk elevations emerged in those aged 40–64 years (low HDL: aHR 1.088, 95% CI 1.051–1.127; elevated triglycerides: aHR 1.015, 95% CI 0.984–1.016) and ≥65 years (low HDL: aHR 1.06, 95% CI 1.027–1.093; elevated triglycerides: aHR 1.034, 95% CI 1.005–1.064), though effect sizes were modest [90].

In contrast, MR analyses (UK Biobank/PRACTICAL) demonstrated no significant associations of LDL cholesterol, HDL cholesterol, triglycerides, apolipoprotein A (apoA), or apolipoprotein B (apoB) with overall, advanced, or EOPC. Notably, elevated lipoprotein(a) [Lp(a)] was associated with increased risks of overall (OR per SD = 1.068; 95% CI 1.005–1.134), advanced (OR per SD = 1.078; 95% CI 0.999–1.163), and EOPC (OR per SD = 1.150; 95% CI 1.015–1.303) [97].

These collectively discordant findings indicate no established role for conventional lipid biomarkers, with the exception of Lp(a), in prostate carcinogenesis, particularly for early‐onset disease, necessitating validation across diverse populations.

##### 4.2.2.4 Clinical Diseases and Treatments

(1) Metabolic syndrome and high blood pressure

The aforementioned South Korean population‐based cohort study [90] revealed that the influence of metabolic syndrome and hypertension on prostate cancer risk escalates progressively with advancing age at diagnosis. Notably, neither condition demonstrated a significant association with prostate cancer incidence in men aged 20–39 years. In contrast, both metabolic syndrome and hypertension conferred significantly increased risk for prostate cancer diagnosis in men aged 40–64 years and those ≥65 years, showing progressively stronger effects across these age groups—a pattern consistently observed throughout the cohort.

(2) LDL‐lowering drugs

Current evidence indicates no significant association between serum LDL levels and overall or EOPC risk, whereas elevated Lp(a) significantly increases susceptibility to both disease subtypes [97]. This conclusion is substantiated by a MR study of LDL‐lowering therapeutic targets (PRACTICAL/UK Biobank) [98]. Genetically proxied inhibition of HMGCR and NPC1L1 demonstrated no significant effect on overall or EOPC risk.

In contrast, PCSK9 inhibition conferred robust protection against both overall (OR per 1‐SD LDL reduction 0.85, 95% CI 0.76–0.96) and EOPC (OR per 1‐SD LDL reduction 0.70, 95% CI 0.52–0.95), with no association observed for advanced disease. Complementary analyses confirmed that lower circulating PCSK9 levels similarly reduced risks for overall (OR per 1‐SD decrease 0.93, 95% CI 0.87–0.997) and early‐onset disease (OR per 1‐SD decrease 0.86, 95% CI 0.74–0.98), again showing no advanced‐stage association.

Mechanistically, genetically proxied PCSK9 inhibition exhibited a strong inverse association with Lp(a) levels (*β* = −0.08, 95% CI −0.12 to −0.05; *p* = 1.00 × 10^−^^5^), independent of BMI or testosterone effects—a relationship absent for HMGCR or NPC1L1 inhibition. These concordant findings implicate Lp(a)‐mediated pathways in prostate carcinogenesis, with PCSK9 modulation demonstrating proportionally greater protective effects for early‐onset disease, warranting dedicated investigation.

(3) SGLT2 inhibition

Notwithstanding current evidence indicating no significant associations between glycemic markers and prostate cancer risk, a recent study investigating SGLT2 inhibition presents conflicting conclusions [96]. Leveraging germline genetic proxies from the PRACTICAL and GAME‐ON/ELLIPSE consortia, genetically predicted SGLT2 inhibition significantly reduced risks of overall (OR per 1‐SD HbA1c reduction 0.56, 95% CI 0.38–0.82), advanced (OR per 1‐SD HbA1c reduction 0.52, 95% CI 0.27–0.99), and EOPC (OR per 1‐SD HbA1c reduction 0.27, 95% CI 0.11–0.71), with the most pronounced effect observed for early‐onset disease. Multivariable analyses confirmed independence from blood cell indices, body weight, systolic blood pressure, LDL cholesterol, and diabetes status. Crucially, SGLT2 inhibition showed no significant association with serum PSA levels (*β* = 0.14, 95% CI −0.30 to 0.03; *p* = 0.11).

Validation in the Shanghai Link Healthcare Database—comprising 81,122 diabetic men with 24,115 SGLT2 inhibitor users propensity score‐matched to 24,115 DPP4 inhibitor controls—demonstrated consistent risk reduction (10‐year HR 0.77, 95% CI 0.61–0.99). Sensitivity analyses indicated comparable protective effects persisted even with short‐term SGLT2 inhibitor exposure (1–6 months).

(4) Sleep disorders

Accumulated evidence indicates no significant association between obstructive sleep apnea or extreme sleep durations and prostate cancer risk [89, 99]. Contrastingly, a population‐based analysis of the Taiwan Longitudinal Health Insurance Database (LHID 2000; *n* = 41,444 males followed 2000–2010) demonstrated that sleep disorders collectively conferred a 1.42‐fold increased prostate cancer risk [100]. Age‐stratified analyses revealed progressive risk attenuation with advancing diagnostic age (*p* for trend = 0.23): significantly elevated hazards were observed in men aged <49 years (aHR = 3.12, 95% CI 1.02–9.58), followed by those aged 50–64 years (aHR 1.50, 95% CI 1.08–2.08), and those ≥65 years (aHR 1.35, 95% CI 1.10–1.65), demonstrating an inverse age‐dependent risk gradient.

(5) Infertility

Meta‐analytical evidence demonstrates significantly elevated prostate cancer risk among males with infertility (infertile vs. fertile: OR 1.49, 95% CI 1.06–2.09)—recently classified as suggestive evidence in an umbrella review [89, 101]. While no studies have directly examined infertility and EOPC, a Swiss national cohort (*N* = 1,181,490 males born 1994–2014) evaluated paternal assisted reproduction exposure and offspring prostate cancer risk [102]. This register‐based study comprised 20,618 in vitro fertilization (IVF), 14,882 intracytoplasmic sperm injection (ICSI), and 1,145,990 natural conceptions. Compared with natural conception, paternal assisted reproduction significantly increased overall prostate cancer risk (aHR: ICSI 1.64 [95% CI 1.25–2.51]; IVF 1.33 [1.06–1.66]). Crucially, EOPC risk elevation exceeded overall risk (aHR: ICSI 1.86 [1.25–2.77]; IVF 1.51 [1.09–2.08]), indicating greater susceptibility in younger populations. Dedicated mechanistic investigations are warranted to elucidate the paternal infertility–EOPC relationship.

(6) HIV

Epidemiological evidence suggests a significantly reduced risk of prostate cancer among individuals with HIV/AIDS compared with uninfected populations (OR 0.74, 95% CI 0.60–0.91), although a recent umbrella review classified this association as weak [89]. A nested prospective cohort study within the United States of 2800 men, with and without HIV infection, aged 40–70 years (22% AA; enrolment 1996–2010) [103] found no significant association between HIV infection and EOPC (adjusted incidence rate ratio [aIRR] 0.89, 95% CI 0.33–2.41) or SOPC (aIRR 1.07, 95% CI 0.51–2.26). Notably, however, among HIV‐positive men within this cohort, the incidence of SOPC was substantially higher than that of EOPC (aIRR 6.98, 95% CI 2.74–17.78). Collectively, these data present conflicting conclusions regarding the association between HIV status and prostate cancer risk. The observed disparity within the cohort study suggests HIV infection might exert a proportionally greater influence on SOPC risk relative to EOPC risk. However, this comparative interpretation does not account for the substantially increasing background incidence of prostate cancer with advancing age.

(7) Clinical prostatitis

A meta‐analysis suggested a significant association between prostatitis and increased prostate cancer risk (OR 1.45, 95% CI 1.13–1.87) [89]. Yet the magnitude of this association varied substantially by diagnostic age, with prostatitis exerting a more pronounced effect on early‐onset than standard‐onset disease. A US case–control study (1992–1994; 36,033 participants including 2263 prostate cancer cases) demonstrated an inverse relationship between prostate cancer diagnosis age and prostatitis‐associated risk (*p*‐interaction = 0.006) [104], revealing distinct age‐stratified risks: relative risk (RR) 1.49 (95% CI 1.08–2.06) for diagnoses <60 years; RR 0.94 (0.76–1.14) for ages 60–68 years; and RR 0.79 (0.64–0.98) for ≥69 years. Methodological constraints require consideration, as prostatitis history was assessed by self‐reported questionnaires without timing of inflammatory episodes. These hypothesis‐generating findings require validation through prospective cohort studies with standardized diagnostic protocols to clarify temporality and reduce recall bias.

(8) In situ melanoma

A retrospective cohort study of Queensland, Australia residents (1982–2012; *n* = 39,872 in situ melanoma survivors) assessed age‐specific risks of secondary malignancies [105]. Among male participants, 1264 prostate cancer cases were identified, indicating a 1.35‐fold elevated risk (SIR 1.35, 95% CI 1.28–1.43). Age‐stratified analyses demonstrated an inverse but nonsignificant association between prostate cancer diagnosis age and SIR magnitude: <50 years (SIR 1.55, 95% CI 1.28–1.87); 50–69 years (SIR 1.39, 1.30–1.49); and ≥70 years (SIR 1.24, 1.12–1.37). Although the risk gradient attenuated with advancing age, no significant heterogeneity was observed across age groups, highlighting the need for mechanistic studies elucidating age‐dependent biological interactions between melanocytic and prostatic neoplasia.

(9) Colorectal cancer

A US SEER database study (1993–2005; 177,834 colon cancer and 85,048 rectal cancer cases) evaluated secondary prostate cancer risk following primary colorectal cancer diagnoses and radiotherapy [106]. Colon cancer was associated with modestly elevated prostate cancer risk overall (SIR 1.03, 95% CI 1.01–1.06), demonstrating three critical patterns: first, a pronounced inverse relationship between prostate cancer diagnosis age and risk magnitude (*p* < 0.0001), with SIR declining from 1.38 (95% CI 1.18–1.60) for diagnoses <50 years, to 1.14 (1.07–1.22) at 50–59 years, 1.06 (1.02–1.10) at 60–69 years, 0.99 (0.95–1.02) at 70–79 years, and 0.96 (0.90–1.02) at ≥80 years; second, radiotherapy‐independent risk elevation in nontreated patients (SIR 1.03, 1.01–1.06); and third, a transient risk peak within 6 months post‐∖diagnosis (SIR 1.48, 1.37–1.59) followed by nonsignificant attenuation.

Conversely, rectal cancer exhibited no overall prostate cancer risk association (all subgroups nonsignificant), though a transient 6‐month postdiagnosis surge occurred (SIR 2.06, 1.87–2.26). Radiotherapy demonstrated a paradoxical protective effect against prostate cancer in rectal cancer patients despite higher malignancy grades and poorer prognosis in irradiated cases (age‐stratified analyses absent).

Collectively, these patterns indicate that early‐onset colon cancer [10] disproportionately elevates EOPC risk independent of radiotherapy, with attenuation at older ages. The short‐term postdiagnosis risk spikes suggest synchronous detection bias, while the age‐risk gradient may reflect shared driver mutations in early‐onset malignancies. Rectal cancer's distinct profile, null baseline risk, transient detection signal, and adverse outcomes with radiotherapy, warrants investigation into anatomical site‐specific carcinogenic pathways.

##### 4.2.2.5 Occupational Factors

(1) Firefighters

A population‐based study from five Nordic countries (Nordic Occupational Cancer [NOCCA] project; cohort aged 30–64 years; Denmark, Finland, Iceland, Norway, and Sweden; 1961–2005; total population 15 million; *n* = 16,422 male firefighters) identified a progressive decline in prostate cancer risk with advancing diagnostic age among firefighters: SIRs were 2.59 (95% CI 1.34–4.52) at 30–49 years, 1.16 (1.04–1.30) at 50–69 years, and 1.09 (0.98–1.21) at ≥70 years [107]. Simultaneously, a US case–control study (29,993 occupationally active male firefighters; 1950–2009) demonstrated significantly elevated prostate cancer risk in men aged <65 years (SIR 1.21, 95% CI 1.10–1.33), with no significant effect in those ≥65 years (SIR 0.96, 0.90–1.03) [108]. Both studies found no significant association between occupational service duration and prostate cancer risk. Notably, the higher frequency of PSA screening in firefighters versus the general population might contribute to these findings.

(2) Other occupational factors

The NOCCA study (1961–2005; *N* ≈ 7.4 million males aged 30–64 years) evaluated occupation‐specific prostate cancer risks with age stratification [109]. Three principal patterns emerged.

First, among men diagnosed before the age of 50 years, four occupations showed significantly elevated risks: technical workers (SIR 1.18, 95% CI 1.01–1.37), administrators (1.41, 1.13–1.73), public safety workers (1.71, 1.23–2.31), and military personnel (1.97, 1.31–2.85). Conversely, teachers (0.68, 0.48–0.94) and gardeners (0.61, 0.35–0.99) demonstrated protective effects.

Second, significant age‐risk heterogeneity was observed: public safety workers (*p* = 0.005) and military personnel (*p* = 0.002) had higher risks in younger versus older patients. Teachers exhibited an inverse pattern (SIR < 500.68 vs. SIR ≥ 501.12), while three other occupations showed no age‐risk variation.

Third, pre‐/post‐1985 analyses revealed temporal divergences exclusively in early‐onset disease: public safety workers’ risk increased post‐1985 (SIR 1.81, 1.23–2.57) versus pre‐1985 (1.47, 0.73–2.63), while military personnel's risk attenuated post‐1985 (1.53, 0.79–2.67) versus pre‐1985 (2.52, 1.44–4.09). These patterns suggest PSA screening may partially explain rising early‐onset risks in public safety roles, while military occupational exposures (e.g., chemical agents, physical stressors) likely drove pre‐1985 risk elevations.

Further stratification of public safety workers identified firefighters as the highest‐risk subgroup for early‐onset disease (SIR 2.59, 1.34–4.52). However, exclusion of firefighters retained significant risk for other public safety roles (SIR 1.50, 1.01–2.15), indicating occupational hazards beyond firefighting exposures. Collectively, these age‐ and era‐dependent risk gradients underscore EOPC's multifactorial etiology.

Additionally, occupational exposure to arsenic has been shown to significantly increase the risk of overall prostate cancer (RR 1.17, 1.07–1.28) [110]. Whether such exposure also elevates the incidence or mortality risk specifically for EOPC requires further investigation.

Taken together, these risk factors highlight the multifactorial nature of EOPC. While some, such as family history and race, likely reflect underlying genetic predisposition, others, including socioeconomic status, metabolic factors, and comorbid conditions, may influence disease risk through environmental and biological pathways.

Notably, the interaction between these factors and germline susceptibility may contribute to the heterogeneity observed in disease onset and clinical behavior, emphasizing the need for integrated risk models.

## 5 Molecular Pathology/Tumor Biology

Prostate cancer pathogenesis is conceptualized as a multistep process involving the accumulation of somatic genomic alterations within normal prostatic epithelium. Distinct from many malignancies, prostate cancer demonstrates a relative scarcity of recurrent point mutations, being predominantly characterized by copy number alterations and structural rearrangements (SRs) [4]. Advanced age constitutes an established epidemiological determinant. As previously delineated, the germline mutation landscape and probable risk factors for EOPC fundamentally differ from those of SOPC. Consequently, malignancies evolving under divergent intrinsic and extrinsic influences are likely to exhibit distinct pathogenic trajectories. Contemporary research corroborates this hypothesis, revealing significant disparities in somatic mutational spectra and TME profiles between EOPC and SOPC tissues (Figure 2B). However, it is critically important to note that current insights into EOPC tumor biology are derived exclusively from studies of localized disease. Key mechanistic questions remain entirely unexplored: specifically, whether the mechanisms underlying metastasis development and progression to castration‐resistant prostate cancer (CRPC) following systemic therapies such as androgen deprivation therapy (ADT) differ between EOPC and SOPC, and if so, the precise nature of these differences.

### 5.1 Somatic Alteration Profile

Collectively, EOPC exhibits a significantly lower overall mutational burden—encompassing both single‐nucleotide variants (SNVs) and SRs—compared with SOPC [15, 111, 112]. However, SRs constitute a significantly higher proportion of the total mutational landscape within EOPC tumor cells than in SOPC [111], suggesting a more dominant role for SRs in EOPC tumorigenesis. Furthermore, SRs in EOPC predominantly occur within open chromatin regions, active enhancer elements, transcription factor binding sites, and sites of active transcription factor binding [15]. In contrast, SRs in SOPC more frequently localize to closed, inactive chromatin regions. Prostate cancer is generally regarded as a highly heterogeneous malignancy of polyclonal origin. Nevertheless, current evidence indicates that EOPC demonstrates a significantly higher probability of monoclonal origin relative to SOPC [15]. We propose that this observation may be explained by a shorter duration of tumor evolution in EOPC, coupled with its proposed primary dependence on androgen signaling for both initiation and progression (discussed subsequently).

In localized prostate cancer, the predominant genomic alteration involves fusions between AR‐regulated promoter regions and loci encoding erythroblast transformation‐specific (ETS) transcription factor family members, with TMPRSS2:ERG predominating [112]. Within general prostate cancer cohorts, TMPRSS2:ERG fusion prevalence is approximately 50% in European‐ancestry localized tumors, contrasting with lower frequencies in Asian and African populations (27–31%) [113, 114, 115, 116, 117]. Notably, EOPC in Europeans demonstrates markedly elevated fusion rates (63–90%) versus SOPC (40–50%) [15, 111, 112, 118, 119, 120, 121], with African‐ancestry EOPC also showing heightened prevalence (41%) [120]. ERG fusion frequency exhibits a significant inverse correlation with patient age (*p* < 0.001) [122], particularly evident in GS ≤ 3+4 tumors [122]. While TMPRSS2:ERG is considered a hallmark of EOPC [111], ERG fusion status shows no significant correlation with either tumor aggressiveness or biochemical recurrence (BCR) in EOPC [111, 119], suggesting that additional somatic alterations beyond ERG fusion are necessary to drive EOPC progression. Other frequently altered chromosomal regions include chromosome 8p (centered at NKX3‐1, 37%) and 3p14 (centered at FOXP1, 30%) [15].

Within EOPC, 13q22 and 8q22 DNA rearrangements occur at 27 and 17% frequencies, their minimal overlapping regions harboring KLF5 and ESRP1 respectively [15]. 13q22 deletion in EOPC significantly reduces KLF5 (a transcription factor promoting cell proliferation) mRNA expression, compounded by promoter hypermethylation. SPOP operates downstream of KLF5, with KLF5 binding the SPOP promoter to regulate its transcription; thus, KLF5 downregulation diminishes SPOP mRNA, attenuating tumor suppression. Critically, this SPOP dysregulation is nonmutational. Conversely, 8q22 duplications elevate ESRP1 mRNA (an RNA‐binding protein implicated in epithelial–mesenchymal transition [EMT] and RNA splicing). Tissue microarray analysis of 11,954 prostate cancers revealed significant associations between high ESRP1 expression and advanced GS, T‐stage, and N‐stage. Survival analysis established high ESRP1 as an independent EOPC prognostic factor, correlating with increased BCR risk irrespective of ERG status.

**FIGURE 3:**
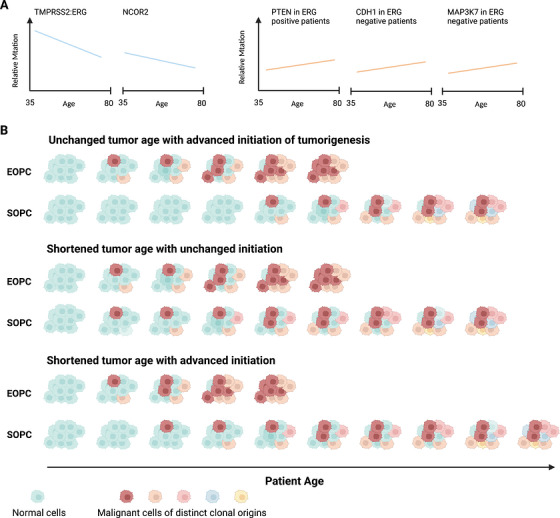
Pathogenic mechanisms in early‐onset prostate cancer. (A) Age‐dependent mutation patterns in prostate cancer driver genes; (B) proposed mechanistic model of early‐onset carcinogenesis, incorporating the three age‐dependent models of prostate carcinogenesis summarized in our work, with tumor cell types representing distinct clonal origins. *Abbreviations*: EOPC, early‐onset prostate cancer; SOPC, standard‐onset prostate cancer.

Age‐associated alterations occur in other drivers (NCOR2, CHD1, MAP3K7, PTEN) (Figure 3A). NCOR2 (an AR corepressor) disruption frequency decreases with advancing age [122]. PTEN disruption increases with age in ERG‐positive tumors but remains age‐independent in ERG‐negative cases [122]. Conversely, CHD1 and MAP3K7 disruption frequencies increase with age in ERG‐negative tumors, showing no age association in ERG‐positive cases [122]. PTEN, CHD1, and MAP3K7 alterations independently predict prognosis beyond ERG status, GS, or T‐stage [122].

Gerhauser et al. propose a somatic alteration sequence for EOPC: initial TMPRSS2:ERG fusion arising on germline mutations and somatic APOBEC3B mutation background, followed most probably by FOXP1 alteration [15]. ETS fusion as the primary event implicates AR signaling or cell cycle pathways as foundational for subsequent molecular progression, though this model requires validation.

That said, prostate cancer is inherently a highly heterogeneous malignancy, characterized by inter‐patient variability and intra‐tumor heterogeneity among distinct foci within the same patient. Even among tumors harboring the TMPRSS2:ERG gene fusion, the underlying mechanisms and molecular pathological features are not uniform. For instance, genomic profiling of 74 primary tumor foci from patients with GS 7 disease identified and validated a recurrent amplification of MYCL [123]. Notably, patients with MYCL‐amplified tumor foci were significantly younger than those without MYCL amplification (*p* = 4.86 × 10^−3^), and MYCL amplification was significantly enriched in ERG‐positive foci (*p* = 1.64 × 10^−2^). These observations implicate MYCL amplification in the pathogenesis of a subset of EOPC cases.

Functionally, in vitro experiments demonstrated that MYCL overexpression or amplification markedly suppresses KLK3 expression, suggesting that the development of certain EOPC cases may bypass canonical AR signaling. Genomically, MYCL amplification was mutually exclusive with MYC alterations and frequently accompanied by TP53 loss. Consistently, mouse models of p53 and Rb1 deficiency, genetic contexts permissive for neuroendocrine transdifferentiation, exhibited a significantly increased frequency of MYCL amplification [124]. Further supporting a potential link between MYCL and neuroendocrine prostate cancer (NEPC), MYCL is highly expressed in NEPC tissues with downregulated MYC and low MYCN expression, and its expression strongly correlates with the neuroendocrine lineage regulators ASCL1 and INSM1, while inversely correlating with adenocarcinoma‐associated genes [125]. Additionally, TMPRSS2:ERG fusions have been detected in primary NEPC specimens (47%, 14 out of 30) in small‐cohort studies [126].

Taken together, these lines of evidence suggest that EOPC may have a higher propensity for primary NEPC compared with SOPC, potentially driven by MYCL amplification in a subset of cases. This hypothesis, however, requires further validation.

### 5.2 TME Characteristics

The designation of TMPRSS2:ERG as a molecular hallmark of EOPC suggests a fundamental reliance on androgen signaling. This dependency is mechanistically supported by in vitro studies where dihydrotestosterone (DHT) treatment of LNCaP cells induced SRs demonstrating significantly greater overlap with EOPC‐specific SRs than those observed in SOPC [111]. Consistently, gene set enrichment analysis of genes within these EOPC‐overlapping SR regions revealed significant enrichment for AR and WNT signaling pathways, enrichment notably absent in SOPC‐overlapping regions. Further corroborating AR's central role, ChIP‐seq data demonstrated markedly increased concordance between AR‐bound SR sites and EOPC‐derived SRs compared with SOPC. Clinically, this mechanistic basis aligns with the observed inverse correlation between tumor AR immunohistochemical intensity and patient age (*p* < 0.001), its positive association with ERG‐positivity magnitude, and the known age‐dependent decline in circulating androgens. Collectively, these findings establish that SRs in EOPC are specifically driven by AR signaling, indicating heightened pathway dependence relative to SOPC.

Extending this molecular framework to the TME, integrated analysis of discovery (scRNA‐seq: 4 high/very‐high‐risk EOPC, 6 high/very‐high‐risk SOPC; ST: 1 per group) and validation cohorts (scRNA‐seq: 5 intermediate/high‐risk EOPC, 5 intermediate/high‐risk SOPC; ST: 1 per group) delineated distinct pathogenic profiles [16]. EOPC tumor epithelia exhibited significantly amplified androgen signaling, manifested through: (1) HER2/EGFR‐mediated AR stabilization enhancing pathway activation; (2) elevated AR activity scores coupled with suppressed NEPC and mesenchymal/stem‐like signatures; and (3) pronounced hypoxia and fatty acid metabolic reprogramming signals. Crucially, ST localized intensified androgen response specifically to myeloid‐infiltrated EOPC tumor regions, implicating microenvironmental crosstalk in sustaining this signaling axis. Within the EOPC TME, lipid‐metabolically dysregulated APOE^+^ macrophages—coexpressing M2 and myeloid‐derived suppressor cell markers—fostered an immunosuppressive niche characterized by increased inhibitory CD4^+^ T cells and decreased stimulatory CD8^+^ T cells. These APOE^+^ macrophages preferentially infiltrated androgen‐responsive tumor epithelia, engaging them via HER2/EGFR interactions, a mechanism conspicuously absent in SOPC. Clinically, high APOE^+^ macrophage signature correlated with inferior overall (OS), disease‐free, and disease‐specific survival, underscoring its prognostic relevance.

Conversely, SOPC epithelia displayed dominant EMT signals driven by interactions with inflammatory cancer‐associated fibroblast (iCAF) subtypes (APOD^+^/CCL2^+^). These interactions activate tumor cell SMAD pathways via BMP/BMPR signaling, thereby reducing AR dependence and promoting an EMT phenotype—a trajectory divergent from the AR‐centric EOPC pathogenesis.

Consequently, within this biological context, current evidence posits localized EOPC as potentially more sensitive to AR‐axis‐targeted therapies. However, critical knowledge gaps persist: the somatic mutational landscape and underlying mechanisms of metastatic EOPC remain poorly characterized, the theoretical sensitivity lacks robust clinical validation, and resistance mechanisms culminating in CRPC are undefined.

### 5.3 Potential Mechanisms of EOPC Pathogenesis

Based on current theoretical frameworks for early‐onset tumor research, the developmental model of EOPC can be summarized into the following three possible theories: (1) unchanged tumor age with advanced initiation of tumorigenesis; (2) shortened tumor age with unchanged initiation of tumorigenesis; and (3) shortened tumor age with advanced initiation of tumorigenesis (Figure 3B). (Adapting Mauri et al.’s concept of tumor age [127], we refine this term in the context of EOPC to denote the interval from malignant transformation of normal prostatic epithelium to progression into clinically significant prostate cancer.)

Analysis using Ki‐67 as a proliferation surrogate revealed equivalent positivity between ERG‐positive and ERG‐negative tumors within the same Gleason grade, with significant differences only observed across different grades. Consistent with this, BCR rates showed no significant divergence by ERG status [111]. These findings collectively indicate comparable tumor cell proliferation kinetics irrespective of ERG fusion status, thereby supporting the first model—where earlier initiation of malignant transformation drives EOPC development.

Contradicting this interpretation, however, multiethnic autopsy data demonstrate synchronous onset of HGPIN across populations (age 20–29 years), while progression rates significantly accelerate in Black males versus White and Asian males [82]. This evidence indirectly suggests that malignant transformation timing may be conserved between EOPC and SOPC, challenging the premise of earlier transformation initiation in EOPC.

Consequently, existing evidence fails to reconcile these discordant observations into a unified pathogenic model for EOPC. Critically, current insights derive predominantly from tissue sequencing analyses without mechanistic experimental validation.

## 6 Sporadic EOPC: An Overlooked Entity

Our understanding of EOPC outlined above is inevitably shaped by a fundamental limitation of the existing literature: most studies have not systematically distinguished between sporadic and hereditary cases. Cohorts are typically assembled without stratification by family history or germline status, and direct comparisons between these two clinically distinct entities are therefore lacking. This is not a deliberate emphasis on hereditary disease, but rather a reflection of how the evidence base has been generated. As a result, much of what we know about EOPC, the germline mutations, somatic alterations, and risk factors discussed in preceding sections, represents a composite drawn from mixed populations in which sporadic and hereditary cases are conflated.

What, then, can be said about sporadic EOPC? In the absence of studies designed specifically to isolate this group, only cautious inferences can be drawn. The environmental and lifestyle risk factors detailed in Section 4.2, elevated BMI, low vitamin D, sleep disorders, occupational exposures, are likely to be more relevant to sporadic disease, given that hereditary EOPC is primarily driven by germline status. The TMPRSS2:ERG fusion appears to characterize both groups, given its consistently high prevalence across unstratified young‐onset cohorts. Whether recurrent alterations such as MYCL amplification, KLF5 downregulation, or ESRP1 duplication are differentially enriched between sporadic and hereditary disease remains unknown; no study has yet performed the requisite stratification.

This distinction is not merely academic. Hereditary EOPC, particularly in BRCA2 carriers, is unequivocally associated with aggressive disease and warrants intensified surveillance. Whether sporadic EOPC carries a similar prognosis, or exhibits greater heterogeneity reflecting its diverse environmental and polygenic underpinnings, is simply not known. If sporadic cases tend to follow a more indolent course, aggressive management paradigms validated in hereditary or mixed cohorts may not be directly transferable, raising the risk of overtreatment. Conversely, if certain sporadic subsets harbor aggressive biology, they may be missed by genetics‐focused screening strategies.

The absence of dedicated studies on sporadic EOPC is a critical gap. Prospective cohorts with systematic ascertainment of family history and germline status are needed to delineate the distinct risk profiles, molecular landscapes, and clinical trajectories of sporadic versus hereditary disease. Only with such stratification can we move beyond the limitations of the current evidence base toward a more complete understanding, one that informs prevention and management for the full spectrum of patients encountered in clinical practice.

Collectively, these molecular alterations delineate a distinct biological landscape in EOPC, characterized by unique genomic profiles and pathway dysregulation. However, the clinical relevance of these findings lies in how such molecular features translate into observable phenotypic differences.

In this context, linking molecular characteristics to clinical presentation is essential for understanding disease behavior in real‐world settings. The following section therefore examines the clinical features of EOPC, with particular emphasis on how underlying molecular mechanisms may contribute to differences in disease onset, progression, and presentation.

## 7 Clinical and Pathological Features

Building on the molecular insights described above, EOPC demonstrates several distinctive clinical features that further differentiate it from later‐onset disease. Current evidence on the clinicopathological features of EOPC derives primarily from retrospective studies, largely single‐center analyses or population‐based cohorts. Notably, sample sizes for EOPC are substantially smaller than for SOPC. Despite these limitations, systematic comparisons are feasible, as summarized in Table 1. Overall, EOPC does not demonstrate a consistent predisposition toward favorable prognostic features compared with SOPC, with significant regional variations evident.

**TABLE 1:** Comparison of clinical characteristics between early‐onset and standard‐onset prostate cancer across geographic regions and overall.

	Early‐onset prostate cancer	Standard‐onset prostate cancer
**Prostate volume (mL)**		
North America	39.1–45.1 [128, 129, 130]	—
Asia	29.0–34.2 [131, 132, 133, 134]	54.1 [131]
Oceania	37.2–44.2 [135, 136]	52.2 [135]
Total	29.0–45.1 [128, 129, 130, 131, 132, 133, 134, 135, 136]	52.2–54.1 [131, 135]
**PSA (ng/mL), rate**		
**<10**		
North America	0.79–0.88 [137, 138, 139, 140, 141]	0.66–0.87 [137, 141]
Europe	0.72–0.82 [142, 143, 144]	0.62 [144]
Asia	0.28–0.58 [134, 145]	0.13 [145]
Oceania	0.89 [135]	0.83 [135]
Total	0.28–0.89 [134, 135, 137, 138, 139, 140, 141, 142, 143, 144, 145]	0.13–0.87 [135, 137, 141, 144, 145]
**≧10**		
North America	0.12–0.21 [137, 138, 139, 140, 141]	0.13–0.27 [137, 141]
Europe	0.18–0.28 [142, 143, 144]	0.29 [144]
Asia	0.42–0.72 [134, 145, 146]	0.63–0.87 [145, 146]
Oceania	0.10 [135]	0.17 [135]
Total	0.10–0.72 [134, 135, 137, 138, 139, 140, 141, 142, 143, 144, 145, 146]	0.13–0.87 [135, 137, 141, 144, 145, 146]
**PSA (ng/mL), mean**		
North America	5.9–30.0 [128, 129, 130, 147, 148, 149, 150]	6.4–28.7 [129, 148, 149, 150]
Europe	5.4–8.7 [151, 152, 153]	8.6–10.2 [152, 153]
Asia	5.6–14.5 [131, 132, 133, 154]	12.2–12.6 [131, 154]
South America	8.2–9.2 [155, 156]	10.7–11.8 [155, 156]
Total	5.4–30.0 [128, 129, 130, 131, 132, 133, 147, 148, 149, 150, 151, 152, 153, 154, 155, 156]	6.4–28.7 [129, 131, 148, 149, 150, 152, 153, 154, 155, 156]
**PSA (ng/mL), median**		
North America	4.4–51.0 [148, 149, 150, 157, 158, 159, 160, 161, 162]	5.4–8.7 [148, 149, 150, 158]
Europe	5.2–5.9 [151, 152, 163, 164]	6.4–7.0 [152, 163]
Asia	4.9–82.4 [131, 154, 165, 166]	8.5–18.0 [131, 154, 165]
South America	7.8 [156]	9.1 [156]
Oceania	3.1–6.3 [167, 168]	6.4–8.2 [167, 168]
Total	3.1–51.0 [131, 137, 148, 149, 150, 151, 152, 154, 156, 157, 158, 160, 161, 162, 163, 164, 165, 166, 167, 168]	5.4–18.0 [131, 148, 149, 150, 152, 154, 156, 158, 163, 165, 167, 168]
**bGS, rate**		
**bGS = 6**		
North America	0.47–0.78 [128, 129, 130, 138, 140, 141, 147, 148, 150, 157, 158, 159, 160]	0.38–0.69 [129, 141, 150, 158]
Europe	0.60–0.69 [142, 144, 152, 153, 163]	0.34–0.59 [144, 152, 153, 163]
Asia	0.08–0.57 [131, 132, 133, 146, 165, 166, 169]	0.14–0.45 [131, 146, 165, 169]
South America	0.78 [155]	0.76 [155]
Oceania	0.27–0.69 [135, 168]	0.28–0.40 [135, 168]
Total	0.08–0.78 [128, 129, 130, 131, 132, 133, 135, 138, 140, 141, 142, 144, 146, 147, 148, 150, 152, 153, 155, 157, 158, 159, 160, 163, 165, 166, 168, 169]	0.14–0.76 [129, 131, 135, 141, 144, 146, 150, 152, 153, 155, 158, 163, 165, 168, 169]
**bGS = 7**		
North America	0.11–0.51 [128, 129, 130, 138, 140, 141, 147, 148, 150, 157, 158, 159, 160]	0.12–0.34 [129, 141, 150, 158]
Europe	0.25–0.33 [142, 144, 152, 153, 163]	0.26–0.56 [149, 152, 153, 160]
Asia	0.21–0.35 [131, 132, 133, 146, 165, 166, 169]	0.28–0.41 [131, 146, 165, 169]
South America	0.13 [155]	0.16 [155]
Oceania	0.26–0.59 [135, 168]	0.32–0.59 [135, 168]
Total	0.11–0.59 [128, 129, 130, 131, 132, 133, 135, 138, 140, 141, 142, 144, 146, 147, 148, 150, 152, 153, 155, 157, 158, 159, 160, 163, 165, 166, 168, 169]	0.12–0.59 [129, 131, 135, 141, 144, 146, 150, 152, 153, 155, 158, 163, 165, 168, 169]
**bGS = 8–10**		
North America	0.00–0.11 [128, 129, 130, 138, 140, 141, 147, 148, 150, 157, 158, 159, 160]	0.03–0.09 [129, 141, 150, 158]
Europe	0.03–0.09 [142, 144, 152, 153, 163]	0.05–0.18 [144, 152, 153, 163]
Asia	0.09–0.69 [131, 132, 133, 146, 165, 166, 169]	0.14–0.57 [131, 146, 165, 169]
South America	0.09 [155]	0.08 [155]
Oceania	0.03–0.14 [135, 168]	0.13–0.24 [135, 168]
Total	0.00–0.69 [128, 129, 130, 131, 132, 133, 135, 138, 140, 141, 142, 144, 146, 147, 148, 150, 152, 153, 155, 157, 158, 159, 160, 163, 165, 166, 168, 169]	0.03–0.57 [129, 131, 135, 141, 144, 146, 150, 152, 153, 155, 158, 163, 165, 168, 169]
**cT, rate**		
**cT1‐2**		
North America	0.85–1.00 [129, 138, 139, 140, 141, 147, 148, 157, 158, 159]	0.89–1.00 [129, 141, 148, 158]
Europe	0.86–0.95 [142, 144, 153, 163]	0.75–0.86 [144, 153, 163]
Asia	0.14–1.00 [132, 133, 146, 165, 169]	0.16–0.85 [146, 165, 169]
Oceania	0.58–1.00 [135, 168]	0.49–0.99 [135, 168]
Total	0.14–1.00 [129, 132, 133, 135, 138, 139, 140, 141, 142, 144, 146, 147, 148, 153, 157, 158, 159, 163, 165, 168, 169]	0.16–1.00 [129, 135, 141, 144, 146, 148, 153, 158, 163, 165, 168, 169]
**cT3‐4**		
North America	0.01–0.15 [138, 140, 141, 148, 158, 159]	0.03–0.11 [141, 148, 158]
Europe	0.01–0.12 [142, 144, 153, 163]	0.14–0.25 [144, 153, 163]
Asia	0.13–0.86 [132, 146, 165, 169]	0.33–0.84 [146, 165, 169]
Oceania	0.00–0.04 [135, 168]	0.00–0.10 [135, 168]
Total	0.00–0.86 [132, 135, 138, 140, 141, 142, 144, 146, 148, 153, 158, 159, 163, 165, 168, 169]	0.00–0.84 [135, 141, 144, 146, 148, 153, 158, 163, 165, 168, 169]
**cN, rate**		
**cN0**		
North America	0.96–0.97 [138, 148]	0.99 [148]
Europe	0.18 [144]	0.17 [144]
Asia	0.36–0.82 [146, 165, 169]	0.49–0.84 [146, 165, 169]
Total	0.18–0.97 [138, 144, 146, 148, 165, 169]	0.17–0.99 [144, 146, 148, 165, 169]
**cN1**		
North America	0.02–0.04 [138, 148]	0.00 [148]
Europe	0.02 [144]	0.02 [144]
Asia	0.18–0.64 [146, 165, 169]	0.16–0.51 [146, 165, 169]
Total	0.02–0.64 [138, 144, 146, 148, 165, 169]	0.00–0.51 [144, 146, 148, 165, 169]
**pGS, rate**		
**pGS = 6**		
North America	0.29–0.75 [128, 129, 130, 137, 147, 148, 149, 150, 157, 158, 159, 160]	0.10–0.72 [129, 137, 148, 149, 150, 158]
Europe	0.24–0.56 [142, 143, 151, 152, 153, 163]	0.12–0.28 [152, 153, 163]
Asia	0.21–0.65 [131, 132, 133, 145, 154]	0.15–0.33 [131, 145, 154]
South America	0.44–0.65 [155, 156]	0.34–0.60 [155, 156]
Oceania	0.14–0.36 [135, 136, 167]	0.16–0.24 [135, 167]
Total	0.21–0.75 [128, 129, 130, 131, 132, 133, 135, 136, 137, 142, 143, 145, 147, 148, 149, 150, 151, 152, 153, 154, 155, 156, 157, 158, 159, 160, 161, 163, 167]	0.10–0.72 [129, 131, 135, 137, 145, 148, 149, 150, 152, 153, 154, 155, 156, 158, 163, 167]
**pGS = 7**		
North America	0.20–0.66 [128, 129, 130, 137, 147, 148, 149, 150, 157, 158, 159, 160]	0.26–0.42 [129, 137, 148, 149, 150, 158]
Europe	0.35–0.71 [142, 143, 151, 152, 153, 163]	0.62–0.79 [152, 153, 163]
Asia	0.39–0.69 [131, 132, 133, 145, 154]	0.41–0.61 [131, 145, 154]
South America	0.17 [155]	0.21 [155]
Oceania	0.51–0.80 [135, 136, 167]	0.54–0.74 [135, 167]
Total	0.17–0.80 [128, 129, 130, 131, 132, 133, 135, 136, 137, 142, 143, 145, 147, 148, 149, 150, 151, 152, 153, 154, 155, 157, 158, 159, 160, 163, 167]	0.21–0.79 [129, 131, 135, 137, 145, 148, 149, 150, 152, 153, 154, 155, 158, 163, 167]
**pGS = 8–10**		
North America	0.02–0.17 [128, 129, 130, 137, 147, 148, 149, 150, 157, 158, 159, 160]	0.02–0.30 [129, 137, 148, 149, 150, 158]
Europe	0.02–0.09 [142, 143, 151, 152, 153, 163]	0.02–0.10 [152, 153, 163]
Asia	0.07–0.21 [131, 132, 133, 145, 154]	0.10–0.26 [131, 145, 154]
South America	0.17 [155]	0.20 [155]
Oceania	0.06–0.07 [135, 136, 167]	0.10–0.12 [135, 167]
Total	0.02–0.21 [128, 129, 130, 131, 132, 133, 135, 136, 137, 142, 143, 145, 147, 148, 149, 150, 151, 152, 153, 154, 155, 157, 158, 159, 160, 163, 167]	0.02–0.26 [129, 131, 135, 137, 145, 148, 149, 150, 152, 153, 154, 155, 158, 163, 167]
**pT, rate**		
**pT1‐2**		
North America	0.65–0.87 [128, 129, 130, 137, 139, 147, 148, 157, 158, 159]	0.36–0.80 [129, 137, 148, 158]
Europe	0.70–0.97 [142, 143, 151, 163]	0.65 [163]
Asia	0.58–0.69 [131, 132, 154]	0.63–0.71 [131, 154]
South America	0.83 [155]	0.72 [155]
Oceania	0.65 [135]	0.60 [135]
Total	0.58–0.97 [128, 129, 130, 131, 132, 135, 137, 139, 142, 143, 147, 148, 151, 154, 155, 157, 158, 159, 163]	0.36–0.86 [129, 131, 135, 137, 148, 154, 155, 158, 163]
**pT3‐4**		
North America	0.13–0.35 [128, 129, 130, 137, 139, 147, 148, 157, 158, 159]	0.11–0.32 [129, 137, 148, 158]
Europe	0.03–0.30 [142, 143, 151, 163]	0.34 [163]
Asia	0.31–0.42 [131, 132, 154]	0.28–0.36 [131, 154]
South America	0.17–0.44 [155, 156]	0.28–0.38 [155, 156]
Oceania	0.35 [135]	0.40 [135]
Total	0.03–0.44 [128, 129, 130, 131, 132, 135, 137, 139, 142, 143, 147, 148, 151, 154, 155, 156, 157, 158, 159, 163]	0.11–0.40 [129, 131, 135, 137, 148, 154, 155, 156, 158, 163]
**pN, rate**		
**pN0**		
North America	0.28–0.89 [128, 148, 159, 160]	0.38 [148]
Europe	0.23–0.66 [142, 151, 163]	0.32 [163]
Asia	0.65 [132]	—
Oceania	0.63–0.95 [135, 136]	0.63 [135]
Total	0.23–0.95 [128, 132, 135, 136, 142, 148, 151, 159, 160, 163]	0.32–0.63 [135, 148, 163]
**pN1**		
North America	0.02–0.10 [128, 148, 149, 158, 159, 160]	0.00–0.08 [148, 149, 158]
Europe	0.03–0.07 [142, 151, 152, 163]	0.03–0.10 [152, 163]
Asia	0.07 [132]	—
Oceania	0.04–0.05 [135, 136]	0.03 [135]
Total	0.02–0.10 [128, 132, 135, 136, 142, 148, 149, 151, 152, 159, 160, 163]	0.00–0.10 [135, 148, 149, 152, 158, 163]
**M‐stage, rate**		
**M0**		
North America	0.97 [138]	—
Europe	0.37 [144]	0.40 [144]
Asia	0.19–0.79 [146, 169]	0.16–0.76 [146, 169]
Total	0.19–0.97 [138, 144, 146, 169]	0.16–0.76 [144, 146, 169]
**M1**		
North America	0.03 [138]	—
Europe	0.07 [144]	0.06 [144]
Asia	0.21–0.81 [146, 169]	0.24–0.84 [146, 169]
Total	0.03–0.81 [138, 144, 146, 169]	0.06–0.84 [144, 146, 169]

Abbreviations: PSA, prostate‐specific antigen; bGS, biopsy Gleason score; pGS, pathological Gleason score; cT, clinical T‐stage; pT, pathological T‐stage; cN, clinical N‐stage; pN, pathological N‐stage; M, M‐stage.

(1) Prostate volume

Prostate volumes in EOPC are typically smaller (29.0–45.1 mL) versus larger volumes in SOPC (52.2–54.1 mL). Direct comparisons from Iran [131] (34.1 vs. 54.1 mL, *p* < 0.001) and Australia [135] (44.2 vs. 52.2 mL, *p* < 0.001) confirm significantly larger prostates in SOPC. This discrepancy likely reflects age‐related physiological changes, supported by the strong correlation between prostate volume and age in healthy males (*r* = 0.44, *p* < 0.001) [170] and autopsy data showing rising benign prostatic hyperplasia prevalence: ∼50% (age 50–59 years), >70% (60–69 years), >90% (≥90 years) [171]. Smaller prostate volumes in Asian EOPC patients compared with North American and Oceanian counterparts, a difference not observed in SOPC, likely reflect baseline racial variations.

(2) PSA level

While prostate volume and PSA increase with age (0.04 ng/mL/year) [172], absolute PSA levels at diagnosis do not differ significantly between EOPC and SOPC. However, using age‐specific PSA thresholds (e.g., <2.5 ng/mL for <50 years vs. <6.5 ng/mL for 70–79 years) [173], EOPC shows proportionally greater elevation relative to age‐adjusted norms. Given PSA's role as an AR activity marker, this further suggests heightened AR signaling in EOPC, consistent with molecular evidence of AR pathway hyperactivation. The alternative hypothesis of larger tumor volumes in EOPC is refuted by an Australian study showing smaller mean tumor volumes in EOPC (1.37 vs. 1.82 cc, *p* = 0.104) and more frequent tumors >2.1 cc in SOPC (20.1 vs. 11.8%, *p* = 0.04) [135]. South Australian population data (*n* = 7018) further confirm increasing tumor volume with age (1.8 cc <50 years, 2.8 cc 50–69 years, 3.0 cc ≥70 years) [167]. Collectively, these findings suggest that enhanced AR activity, rather than tumor bulk, drives elevated PSA levels in EOPC. Reported higher rates of PSA >10 ng/mL in Asian cohorts require cautious interpretation due to confounding from a Japanese study exclusively enrolling Stage IV patients [146]. This apparent discordance may be particularly relevant in younger patients, in whom PSA levels might be influenced not only by tumor burden but also by distinct biological characteristics. As such, PSA interpretation in early‐onset disease may require a different clinical perspective compared with older populations.

(3) Gleason score

Pooled evidence from North America, Asia, South America, and Oceania indicates no significant differences in biopsy GS between EOPC and SOPC. In contrast, European cohorts show a tendency toward lower‐grade GSs in EOPC. Studies from Germany (*n* = 13,268, cutoff 50 years) [142], Lithuania (*n* = 2150, cutoff 55 years) [163], and Romania (*n* = 202, <45 vs. >65 years) [153] revealed significantly higher rates of GS 3+3 and lower proportions of ≥GS 3+4 in EOPC biopsies (*p* = 0.09, 0.46, 0.0004). Similarly, a large German study (*n* = 16,049) [152] and Swiss population analysis (*n* = 46,017) [144] demonstrated a progressive age‐dependent increase in higher GSs (≥3+4) (*p* < 0.001). Postprostatectomy GSs align with biopsy findings. Outside Europe, no significant pathological GS differences exist. However, European EOPC cohorts exhibit markedly higher rates of GS 3+3 (24–56 vs. 12–28% in SOPC) and a trend toward fewer Gleason 7 tumors (35–71% vs. 62–79%). These regional variations necessitate age‐ and geography‐specific risk stratification.

(4) T‐stage

Clinical and pathological T‐stage distributions within geographic regions show minimal differences between EOPC and SOPC. Marked intercontinental variations exist: North American and European patients are predominantly c/pT1‐2, while nearly half of Asian patients present as c/pT3‐4. This discrepancy may partly stem from a Japanese clinical T‐stage study exclusively including Stage IV patients [146] and a Korean pathological T‐stage study limited to clinically localized/advanced cases [132]. Excluding these, Asian EOPC patients exhibited cT1‐2 in 42–100% and cT3‐4 in 13–58%; SOPC patients showed cT1‐2 in 58–85% and cT3‐4 in 33–42%. Pathological T‐stage proportions remained unchanged. The persistent regional T‐stage disparity, even postadjustment, likely reflects delayed PSA screening adoption in Asia versus Western regions, resulting in later‐stage diagnoses, consistent with broader geographic differences [2].

(5) N‐stage

Comparisons of nodal (N) staging across regions are limited by sparse data; most studies analyzed small subsets. Intraregionally, clinical N‐stage (cN) distributions are largely concordant between EOPC and SOPC. Pathologically (pN), no significant differences in pN1 rates exist globally. While initial North American and European studies suggested higher pN0 rates in EOPC, this disparity primarily reflects fewer SOPC patients reporting pN0 data. A European study found comparable pN0 rates (23 vs. 32%, *p* = 0.85) [163], while a US study showed substantially higher rates of unassessed N‐stage in SOPC (62 vs. 34%) [148], highlighting challenges in interpreting nodal disparities without robust, standardized reporting.

(6) M‐stage

Metastatic (M) stage distributions are consistent within geographic regions. Seemingly low European M0 rates (e.g., EOPC 23–45% vs. SOPC 28–50%) reflect incomplete M‐stage data (>50% undocumented) [144]; reported European M1 rates do not differ significantly from North America. For Asian cohorts, excluding the Stage IV‐biased Japanese study [146], a population analysis (n = 28,039) revealed M0 rates of 79% (EOPC) and 76% (SOPC), with M1 rates of 21 and 24% [169], markedly higher than Western regions. This persistent burden of advanced‐stage diagnoses in both EOPC and SOPC underscores regional delays in PSA screening and diagnostic infrastructure [2].

## 8 Survival

### 8.1 Survival Outcomes

**FIGURE 4:**
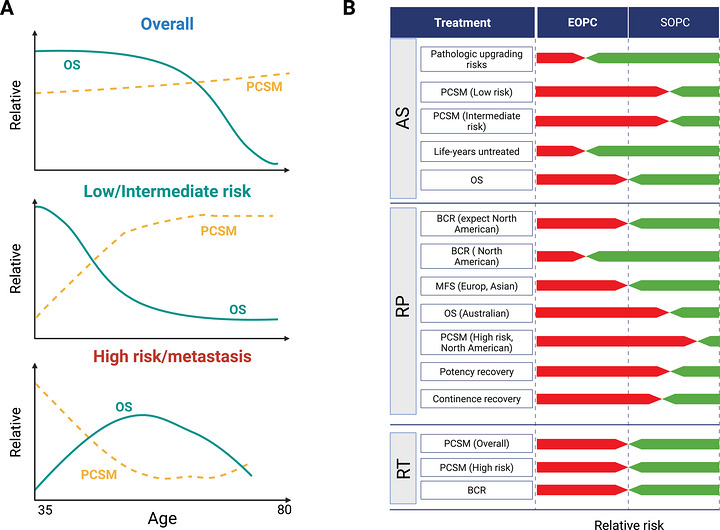
Prognostic landscape of early‐onset prostate cancer. (A) Age‐stratified overall survival (OS) and prostate cancer‐specific mortality (PCSM); (B) comparative treatment response to definitive local therapy: early‐onset versus standard‐onset disease. *Abbreviations*: OS, overall survival; PCSM, prostate cancer‐specific mortality; AS, active surveillance; RP, radical prostatectomy; RT, radiotherapy; MFS, metastasis‐free survival; BCR, biochemical recurrence.

Collectively, no significant differences exist in OS, PCSM, or BCR between EOPC and SOPC patients. However, patients aged ≥65–70 years, particularly those ≥75 years, demonstrate significantly worse OS (Figure 4A). This age‐dependent disparity is evidenced by multivariable Cox analysis of 28,039 patients from the Taiwan Cancer Registry (2008–2016), where patients <55 years showed comparable overall mortality (OM) to the 60–69 years reference group (HR 1.02, 95% CI 0.84–1.23), while those aged 70–74 years (HR 1.46, 95% CI 1.36–1.58) and ≥75 years (HR 2.75, 95% CI 2.57–2.91) exhibited progressively elevated OM risk [169]. Critically, this pattern of stable mortality risk up to the age of ∼65 years followed by a sharp increase aligns with SEER database analyses (1973–1997; *n* = 289,809), which confirmed no OS differences among patients aged 40–64 years but revealed escalating mortality from the age of 65 years onward (HR 1.48 for 65–69 years to HR 4.26 for ≥80 years versus 40–44 years reference) [174]. Importantly, PCSM risks remain comparable across the examined age groups, as demonstrated in the CaPSURE cohort (*n* = 13,805; data through 2008): ≤55 years reference versus 56–65 years (HR 0.99, 95% CI 0.96–1.02), 66–75 years (HR 1.06, 95% CI 0.58–1.95), and ≥75 years (HR 1.03, 95% CI 0.56–1.91) [147]. This lack of significant age association for PCSM is further supported by the Taiwan data, which, despite describing a symmetrical U‐shaped association [169], showed hazard ratios crossing 1.0 (indicating no statistically significant difference) for PCSM across age groups compared with the reference.

The OS decline in older patients is mechanistically driven by comorbidity‐associated other‐cause mortality (OCM). Baseline comorbidity indices in CaPSURE showed a threefold rise in patients with scores ≥3, from 11% (<55 years) to 33% (≥75 years) [147], a gradient reinforced by SEER data (1994–1995; *n* = 3183) where severe comorbidity prevalence increased from 6% (<55 years) to 18% (≥76 years; *p* < 0.001) [175]. Critically, this escalating comorbidity burden drives a dose‐dependent increase in OCM risk: Longitudinal follow‐up established a comorbidity dose–response for OCM, with 14‐year mortality rising from 24% (0 comorbidities) to 57% (≥3 comorbidities). Multivariable competing‐risk analyses confirmed progressively elevated hazards (vs. comorbidity‐free): 1 condition (HR 1.2, 95% CI 1.0–1.4), 2 conditions (HR 1.7, 1.4–2.0), ≥3 conditions (HR 2.4, 2.0–2.8) [175]. Notably, comorbidity's absolute effect on OCM attenuated in patients <60 years, while PCSM remained independent. Conversely, BCR exhibits no significant age‐based variation: SA‐PCCOC data (*n* = 7018; 1998–2012) confirmed equivalent BCR rates across age strata (≤50 years: *n* = 182; 50–70 years: *n* = 3376; ≥70: *n* = 3460) [168]. Thus, while OCM rises steeply with age due to increasing comorbidity burden and its dose‐dependent mortality effect, BCR risk remains consistent regardless of patient age.

### 8.2 Prognostic Disparities in Advanced Disease

Younger patients with advanced prostate cancer (Stage III: cT3N0M0; Stage IV: cT4/N1/M1), high‐risk disease (NCCN criteria), or GS ≥8 exhibit significantly worse overall and prostate cancer‐specific survival (OS/PCSM). Critically, this age‐dependent divergence is modulated by tumor grade and stage. SEER analysis (1988–2003; *n* = 318,774) revealed opposing patterns: In GS ≤ 7 disease, OS progressively deteriorated with aging (vs. 35–44 years: 45–54 HR 1.45, 95% CI 0.95–2.22; 55–64 HR 2.10, 95% CI 1.38–3.19; 65–74 HR 3.53, 95% CI 2.32–5.37) while PCSM remained age‐invariant; conversely, in GS 8–10 disease, older cohorts (45–74 years) showed superior OS/PCSM (e.g., 55–64 years: OS HR 0.71, 95% CI 0.54–0.82, PCSM HR 0.58, 95% CI 0.44–0.77 vs. 35–44 reference) [176]. This survival advantage for older high‐grade patients extends to Stage IV disease, with ≥55‐year GS ≤ 7 patients showing significantly lower PCSM risk (e.g., 65–74 HR 0.60, 95% CI 0.38–0.96) [176].

Metastatic burden further amplifies the survival disparity. SEER analyses (2004–2014; cT3‐4 patients: <50 years, *n* = 1507; ≥50, *n* = 34,833) quantified elevated PCSM risk in <50 years (HR 1.62, 95% CI 0.56–2.05), particularly for cT3b (HR 2.18, 95% CI 1.27–2.72) and cT4 (HR 2.44, 95% CI 1.08–2.44) [177]. These findings are refined by PCBaSe (1998–2012; metastatic patients <50 years: *n* = 919; 50–59 years: *n* = 45,098), where younger metastatic patients had 28% higher PCSM risk (HR 1.28, 95% CI 1.01–1.62) [144]. Conversely, younger age confers protection in nonmetastatic contexts: SEER analysis (1995–2003; *n* = 14,697) showed markedly superior OS (HR 0.05–0.18) and PCSM (HR 0.06–0.20) in <50‐year cT4N0M0/TxN1M0 patients versus metastatic counterparts [178]. Ultimately, Taiwan data unify these patterns through a risk‐stratified model [169]: Low/intermediate‐risk patients maintain stable OS until ages 60/65 years before rapid escalation, whereas high‐risk disease exhibits bimodal age‐specific mortality—with peaks in youth (attributed to aggressive tumor biology) and advanced age (driven by senescence‐related OCM), evidenced by OS nadir at 60 years.

### 8.3 Sociodemographic Modifiers

Early‐onset survival is codetermined by sociodemographic and clinicopathological factors. Unmarried/widowed status, low income, and lack of insurance/Medicaid coverage predict worse OS [138, 179, 180], while advanced AJCC stage, higher GSs, and elevated PSA drive progressive OS decline [138, 166, 178, 179, 180]. However, racial disparities show contradictory patterns: The National Cancer Database (2004–2018; ≤55 years, *n* = 122,563) reported nonsignificant OS trends favoring Black versus Caucasian patients (HR 0.94, 95% CI 0.88–1.00), with other races exhibiting better survival (HR 0.81, 0.68–0.97) [179]. In contrast, SEER data (2010–2015; ≤55 years, *n* = 23,730) showed superior OS in Caucasians versus Black patients (HR 0.75, 95% CI 0.63–0.90) [138], whereas another SEER cohort (2004–2016; <50 years, *n* = 8259) found worse OS for both Caucasians (HR 1.79, 95% CI 1.07–3.00) and Black patients (HR 2.36, 95% CI 1.39–4.01) versus other races [180]. Stage‐stratified analyses reveal: No racial OS differences in Stage IV patients <50 years, but Black patients ≥50 years had significantly worse OS than Caucasians [178]. These inconsistencies underscore multifactorial racial disparities intersecting biologic, socioeconomic, and healthcare‐access variables.

Taken together, these survival patterns highlight that EOPC is not only characterized by distinct biological behavior but also by clinically meaningful differences in disease trajectory and outcomes compared with standard‐onset disease. Importantly, the observed heterogeneity in survival outcomes, shaped by tumor biology, genetic predisposition, and treatment responsiveness, has direct implications for clinical decision‐making.

These considerations underscore the need for tailored management strategies in EOPC, prompting a closer examination of current diagnostic and therapeutic approaches, as discussed in the following section.

## 9 Management

Active interventions, including active surveillance (AS), significantly improve cancer‐specific survival for intermediate/low‐risk patients with ≥10‐year life expectancy and high‐risk patients with ≥5‐year life expectancy [173]. The clinicopathological profile of EOPC reveals that most cases present with localized disease at diagnosis, closely resembling SOPC. Critically, patient life expectancy and health status remain pivotal determinants of therapeutic strategy [181]. Consequently, EOPC exhibits distinct patterns in both primary treatment selection and posttreatment survival outcomes compared with standard‐onset disease (Figure 4B).

### 9.1 Treatment Selection

Younger patients demonstrate a stronger preference for surgical intervention over AS or radiotherapy, as evidenced by SEER database analyses (2004–2013) showing significantly higher RP rates in patients ≤50 years compared with older cohorts (70.5 vs. 36.9%, *p* < 0.001) and lower external beam radiotherapy (EBRT) utilization (7.8 vs. 23.7%, *p* < 0.001) [161]. This surgical predilection is further supported by pre‐2004 SEER data indicating increased RP rates in patients <55 years (*p* < 0.001), contrasting with higher radiotherapy and conservative management (AS/watchful waiting [WW]/ADT) frequencies in older populations [176]. The CaPSURE study (*n* = 13,805 through 2008) reinforces this pattern, documenting RP rates of 85% in patients <55 years that progressively decline to 3% in those >75 years [147]. Concordant evidence from SA‐PCCOC (*n* = 7018, 1998–2012) [168] and a single‐center analysis (*n* = 432) [182] confirms consistently low AS adoption (<5%) in population‐based studies.

Temporal shifts, however, reveal evolving therapeutic preferences: RP utilization declined from 76.5% (2004) to 62.4% (2013) (*p* = 0.008), while conservative management increased from 8.9 to 25.2% (*p* < 0.001) [161]. This shift predominantly affects intermediate/low‐risk patients. Supporting this, SEER data (2010–2015) demonstrate rising AS/WW adoption among low‐risk patients ≤55 years (8.61–34.56%; *p* trend < 0.001), paralleled by declining RP (69.71–48.38%) and radiotherapy rates (21.68–17.06%) [183]. Australian PCOR‐Vic registry data (*n* = 3201, 2009–2016) confirm accelerated conservative management uptake in younger low‐risk patients, with AS rates increasing from 37 to 66% [184]. For intermediate‐risk patients (NCDB, *n* = 289,584, 2010–2020), AS adoption shows gradual increase (5.5% overall), though less pronounced in early‐onset cohorts [174].

Beyond treatment selection, age‐dependent disparities in treatment initiation timelines emerge as critical determinants. Analysis of 316,070 patients aged ≤64 years (National Cancer Database, 2004–2017) demonstrates significantly fewer treatment delays (≥180 days) in younger men (18–55 years) versus those aged 56–64 years (aOR 0.92, 95% CI 0.88–0.97; *p* = 0.001) [185]. Racial stratification within this cohort reveals divergent patterns: White patients exhibit age‐dependent delay rates (3.96% [95% CI 3.81–4.11] in ≤55 years vs. 4.27% [4.18–4.36] in 56–64 years), whereas Black patients show no significant age‐related variation (7.47% [7.11–7.83] vs. 7.00% [6.74–7.26]). Notably, among patients ≤55 years, racial disparities markedly amplify delays, with Black men experiencing nearly double the delay frequency of White counterparts (7.39 vs. 3.96%; aOR 1.95, 95% CI 1.81–2.09; *p* < 0.001). This disparity persists across insurance strata, including privately insured cohorts (7.02 vs. 3.56%; aOR 2.05, 95% CI 1.89–2.23; *p* < 0.001), establishing age as the principal modifier of treatment urgency while highlighting racial inequities disproportionately exacerbating delays in younger populations.

### 9.2 Active Surveillance

#### 9.2.1 Age Disparities in AS Selection

Younger patients with low‐ and intermediate‐risk prostate cancer exhibit pronounced age‐dependent disparities in AS/WW selection, demonstrating progressively lower adoption rates with decreasing age. This gradient is quantified by analysis of 50,302 low‐risk patients (Nonpublic SEER, 2010–2015), where patients ≥55 years had 63% higher AS likelihood than those <55 years (OR 1.63, 95% CI 1.54–1.72) [183]. The disparity intensifies substantially with advancing age, evidenced by Prostate Cancer Outcomes Registry Victoria data (PCOR‐Vic; 2009–2016, *n* = 3201) showing AS odds ratios escalate from 1.61 (55–59 years) to 11.35 (>75 years) per 5‐year increment compared with <55 years (*p* < 0.001) [184]. For intermediate‐risk disease, AS adoption remains critically low overall (National Cancer Database, 2010–2020; *n* = 289,584: 5.5%) [186], nevertheless increasing significantly with age (OR 1.13 for 50–60 years to 11.12 for 80–90 years vs. <50 years; *p* < 0.01).

#### 9.2.2 Clinicopathologic Determinants

AS selection patterns are driven primarily by clinicopathologic factors. Among younger low‐risk patients, each additional year increases AS likelihood by 2% (OR 1.02/year, 95% CI 1.01–1.04), while those with 2 versus 3 positive biopsy cores exhibit 3.26‐fold higher AS rates (OR 3.26, 95% CI 2.87–3.70) [183]; insurance status shows no significant influence [183, 184]. For intermediate‐risk patients, AS preference inversely correlates with disease aggressiveness: Gleason 3+3 patients demonstrate 36.44‐fold higher AS adoption than GS 4+3 (OR 36.44, 95% CI 33.88–39.20), whereas cT2b/cT2c patients have 65% reduced adoption versus cT1a–T2a (OR 0.35) [186]. Strikingly, clinician biases amplify these trends, as demonstrated by a 2017 survey of 692 urologists/radiation oncologists (37.3% response rate): AS recommendations were minimal (0–0.6%) for patients <55 years with GS 4+3 and PSA 10–20 ng/mL but rose to 29.0–35.4% for patients >75 years with GS 3+4 and PSA <10 ng/mL [187].

#### 9.2.3 Safety Implications and Outcomes

Paradoxically, younger patients demonstrate reduced pathologic reclassification risk during AS monitoring (Urological Oncology Database, 1992–2014; *n* = 1433: HR 0.97 per year decrease, 95% CI 0.96–0.99 for low‐risk patients) [188], yet experience diminished survival benefits. SEER data (2010–2015; *n* = 18,760 favorable intermediate‐risk patients) reveal older cohorts face significantly higher upgrading risks: GS 3+4 patients aged 70–79 years show 3.86‐fold increased risk versus 40–49 years (HR 3.86, 95% CI 1.33–14.11), while cT2b‐c patients aged 70–79 years exhibit HR 1.76 (95% CI 1.41–2.19) [189]. Crucially, Swedish PCBaSe registry data (1992–2014; *n* = 23,655) demonstrate younger patients experience higher PCSM (low‐risk: 13% at the age of 55 years vs. 6% at 70 years; intermediate‐risk: 15 vs. 7%) and fewer life‐years untreated (intermediate‐risk: 29% at the age of 55 years vs. 60% at 70 years) [190], despite comparable OS between AS and definitive therapy in young low‐risk cohorts (nonpublic SEER: HR 1.14, 95% CI 0.57–2.30) [183].

These findings establish greater safety concerns for younger low/intermediate‐risk patients managed with AS due to attenuated survival benefits relative to older cohorts, necessitating age‐adapted protocols incorporating stringent biomarker monitoring and comorbidity‐adjusted stratification.

### 9.3 Radical Prostatectomy

#### 9.3.1 Age‐Specific Oncological Outcomes Reveal Pronounced Geographical Heterogeneity

Contemporary multinational evidence delineates substantial geographical heterogeneity in RP outcomes for EOPC, reflecting disparities in clinical practices, screening protocols, and biological characteristics.

European studies, predominantly single‐center retrospective analyses from Germany, demonstrate a paradoxical association between younger age and favorable BCR outcomes. A landmark German cohort (1992–2011, *n* = 13,268) reported superior 10‐year BCR‐free survival in patients aged <50 years compared with older counterparts (63.0 vs. 58.3%; *p* = 0.006) [142], a finding replicated in a larger German study (2006–2014, *n* = 15,049) showing age‐stratified 8‐year BCR‐free survival rates of 80.2% (≤45 years), 75.2% (45–65 years), and 70.5% (>65 years; *p* < 0.001) [152]. Nevertheless, multivariable Cox analyses attenuated these differences, with age <50 years failing to independently predict BCR (HR = 0.90; 95% CI 0.90–1.20) [142]. Notably, metastasis‐free survival (MFS) remained comparable across age groups (5‐year rates: ≤45 years 98.7%, 45–65 years 95.3%, >65 years 94.9%; *p* = 0.429), suggesting age‐independent metastatic potential in localized disease [152].

Conversely, North American studies, largely derived from pre‐PSA and early PSA‐era cohorts, reveal conflicting temporal trends. A single‐center US analysis (1982–2001, *n* = 2897) demonstrated progressive BCR deterioration with advancing age, with 15‐year rates declining from 69% (<50 years) to 58% (≥70 years; *p* = 0.003) [140]. Multicenter data (1988–2002, *n* = 1753) corroborated this pattern, showing patients aged 51–60, 61–70, and >70 years exhibited escalating BCR risks versus those aged <50 years (HR = 2.22, 2.05, 3.49; all *p* < 0.05) [150]. Modern CPDR database analysis (1989–2009, *n* = 12,081) confirmed this gradient, revealing a 32–52% increased BCR risk per decade of ageing (50–59 vs. <50 HR = 1.32; 60–69 HR = 1.47; ≥70 HR = 1.52; all *p* < 0.05) [191]. Paradoxically, contemporary SEER data (2004–2013, *n* = 407,599) indicated inverted mortality trends: older patients exhibited elevated PCSM (HR 1.24, 95% CI 0.99–1.55) alongside markedly increased OCM (HR 3.02, 95% CI 2.59–3.53), complicating age‐based prognostic interpretations [161].

Asian studies uniformly challenge Western patterns. Iranian (2005–2018, *n* = 644) [146], Korean (2001–2017, *n* = 622) [132], and Singaporean (1998–2016, *n* = 1120) [154] cohorts demonstrated no significant age‐related differences in BCR (HR 0.93, 95% CI 0.66–1.31) [133], local recurrence (*p* = 0.441), or metastasis (*p* = 0.654), even after NCCN risk stratification [133]. This consistency across diverse healthcare systems suggests intrinsic biological or screening‐related distinctions from Western populations.

Australian registry analyses introduce nonlinear age effects. Victorian data (2004–2014, *n* = 14,686) revealed optimal survival in middle‐aged cohorts (45–54 vs. 65–74 years: OS HR 0.33, 95% CI 0.20–0.56), while younger patients (35–44 years) trended toward elevated PCSM (HR 3.39, 95% CI 0.44–25.78) [167]. South Australian data (1998–2012, *n* = 7018) further suggested attenuated BCR risks in younger patients (≤50 vs. ≥70 HR 0.69; 95% CI 0.39–1.24), highlighting region‐specific outcome profiles [168]. Brazilian retrospective series (1990s) complete this geographical mosaic, demonstrating age‐independent BCR patterns (*p* = 0.47) [155, 156].

Critical exceptions emerge in high‐risk subgroups. SEER analysis of cT3‐4, GS8‐10 patients (2004–2014, *n* = 36,340) demonstrated younger patients (<50 years) experiencing a 77% increased PCSM risk versus older counterparts (HR 1.77, 95% CI 1.01–3.01), contrasting sharply with historical patterns where extreme youth (35–44 years) paradoxically predicted superior outcomes in GS8‐10 disease (OS HR 0.47–0.86; PCSM HR 0.35–0.29) [175].

This reversal underscores the dual influence of tumor biology and competing mortality risks across age strata. Collectively, these findings delineate three key determinants of age‐related outcomes: temporal cohort effects (divergent pre‐PSA era vs. modern screening period findings), surgical standardization disparities (European single‐center vs. Asian registry data), and biological heterogeneity (evident in high‐risk subgroups).

#### 9.3.2 Survival Outcomes and Biological Determinants in Young Patients Undergoing RP

Contemporary evidence reveals complex interactions between RP, disease biology, and demographic factors in EOPC. Conflicting survival benefits emerge from population‐based studies: A SEER analysis (2010–2015; *n* = 23,730; age ≤55 years) demonstrated significantly worse OS for nonsurgical versus RP‐treated patients (HR 2.61, 95% CI 2.03–3.34) [138], while another SEER cohort (2004–2016; *n* = 8259; age <50 years) showed no significant OS (HR 0.95, 95% CI 0.75–1.21) or PCSM benefit (HR 0.91, 95% CI 0.66–1.26) with RP compared with untreated patients [180]. This discrepancy is primarily attributed to confounding by indication, as untreated cohorts predominantly comprise low‐risk patients with favorable prognoses [180]. Stage‐specific effects further clarify this paradox: In metastatic disease (1995–2003 SEER: 615 cT4N0M0, 3189 TxN1M0, 10,893 TxNxM1), RP provided significant OS (HR 0.43, 95% CI 0.24–0.78) and PCSM (HR 0.36, 95% CI 0.19–0.69) benefits for patients aged <50 years [178].

Biological heterogeneity critically modulates outcomes. Molecular analysis of 118 RP patients aged <50 years (2000–2005) revealed BCR events occurred exclusively in ERG‐positive cases (19.2 vs. 0% in ERG‐negative; *p* = 0.078) despite no metastatic events during 65.7‐month follow‐up [192]. Racial disparities present nuanced patterns: A US surgical cohort (2000–2011; *n* = 650) showed comparable overall BCR rates between Black and White patients (*p* = 0.9), but suggested accelerated progression in Black patients with locally advanced disease (2‐year BCR‐free survival: 56 vs. 75%; p = 0.052) [160]. Genetic predisposition analyses from a French cohort (1994–2004; *n* = 110) revealed nonsignificant survival gradients (5‐year PSA‐free survival: 93% hereditary, 85% familial, 80% sporadic; *p* = 0.46–0.52) [143], while established pathological predictors, T‐stage, GS, and perineural invasion, retained prognostic significance [128, 157].

Collectively, these findings necessitate risk‐adapted algorithms integrating clinicopathological variables, molecular biomarkers, and demographic factors to optimize management of EOPC.

#### 9.3.3 Age‐Dependent Trajectories of Postprostatectomy Functional Recovery

The influence of patient age on functional recovery after prostatectomy demonstrates a conserved temporal pattern across studies, with early youth‐driven advantages progressively diminishing over time. Initial evidence from a German cohort (1992–2011, *n* = 13,268) established age <50 years as an independent predictor of superior 12‐month continence (aOR 2.5, 95% CI 1.5–4.2) and potency recovery (aOR 2.2, 1.6–3.2) after adjusting for nerve‐sparing rates, despite younger patients having higher bilateral neurovascular bundle preservation (77.4 vs. 61.5%, *p* < 0.001) [142]. This gradient intensified in a subsequent German analysis (2006–2014, *n* = 15,049), where patients aged ≤45 years achieved 97.4% 1‐year continence versus 84.7% in those aged >65 years (aOR 11.4, 95% CI 6.2–21.0), confirming age‐dependent neural recovery capacity [152].

US data corroborated these early advantages while delineating attenuation timelines. A surgical series (2001–2005, *n* = 369) identified age as the sole predictor of continence restoration at 3, 6, and 12 months (*p* = 0.01–0.03), with potency recovery showing parallel age dependence at 3/6 months (*p* = 0.01–0.02) but losing significance by 12 months (*p* = 0.10) [129]. The CaPSURE registry (1999–2003, *n* = 1143) further validated age ≤55 years as an independent factor influencing early functional recovery [193]. Retrospective analyses specifically demonstrated resolution of age‐related disparities by 24 months post‐RP, with patients aged ≤50 and >50 years achieving comparable continence (94 vs. 92%, *p* = 0.21) and potency rates (78 vs. 72%, *p* = 0.15) [147].

Contemporary stratification of early‐onset cohorts (2008–2019, *n* = 2243) refined this trajectory: Among patients aged ≤55 years, 1‐year continence varied minimally across ≤45 (98.6%), 46–50 (97.2%), and 51–55‐year (96.0%) subgroups (aHR 1.07, 95% CI 0.97–1.19), while potency declined incrementally (89.3, 82.3, 75.2%; aHR 1.21, 95% CI 1.08–1.36) [128]. These patterns align with the established temporal model, youth accelerates initial neural repair, but functional outcomes equilibrate within 24 months across age strata.

### 9.4 Radiotherapy

Contemporary analyses demonstrate no significant differences in BCR or PCSM between younger and older prostate cancer patients treated with radiotherapy. Critically, however, advanced age independently predicts increased OCM. Analysis of 407,599 SEER database patients (2004–2013), including 18,387 diagnosed aged <50 years, confirmed nonsignificant PCSM differences across age groups for brachytherapy (BT: HR 2.06, 95% CI 0.85–4.99) and EBRT (HR 1.13, 95% CI 0.79–1.62), while OCM risks rose substantially (BT: HR 4.37, 95% CI 2.77–6.88; EBRT: HR 2.69, 95% CI 2.06–3.51) [161]. Extending these observations, among cT3‐4 patients, radiotherapy provided no PCSM advantage for younger individuals (HR 1.45, 95% CI 0.57–3.70) [177]. This consistent absence of age‐dependent PCSM/BCR disparities was further evidenced in the CPDR database (1989–2009; *n* = 12,081), which showed no escalation in BCR risk by age post‐EBRT (50–59 vs. <50: HR 1.49, 95% CI 0.36–6.14; 60–69: HR 1.43, 95% CI 0.36–5.75; ≥70: HR 1.55, 95% CI 0.39–6.23) [191], a pattern replicated in the SA‐PCCOC cohort (1998–2012; *n* = 7018), where patients aged ≤50 years (*n* = 182) exhibited outcomes comparable to older groups [168].

Importantly, beyond merely lacking differential efficacy by age, radiotherapy may confer no survival benefit or even demonstrate detriment in early‐onset disease. Analysis of 23,730 patients aged ≤55 years (SEER 2010–2015) revealed no OS advantage for radiotherapy (HR 1.10, 95% CI 0.91–1.33) [138]. More concerningly, in Stage IV early‐onset cases (SEER 1995–2003: 615 cT4N0M0, 3189 TxN1M0, 10,893 TxNxM1), radiotherapy significantly worsened OS (HR 1.42, 95% CI 1.11–1.83) and PCSM (HR 1.40, 95% CI 1.07–1.82) versus no treatment [178].

Additionally, studies investigating the impact of ERG fusion status on radiotherapy response suggest that high‐risk prostate cancers harboring ERG fusions may exhibit reduced sensitivity to radiation. Dal Pra et al. analyzed 126 patients with T1‐2, GS 6–7 prostate cancer treated with radiotherapy and found no significant difference in BCR between ERG‐negative and ERG‐positive patients [194]. In contrast, Fontugne et al. examined 80 patients with T1‐3, GS 6–8 disease and reported that ERG fusion positivity was significantly associated with an increased risk of BCR in multivariable Cox analysis (HR 2.60, 95% CI 1.62–111.9) [195]. Given that ERG fusions are present in 63–90% of EOPC cases, these findings further support the possibility that high‐risk EOPC may be less responsive to radiotherapy.

Furthermore, when compared directly with RP, radiotherapy demonstrates inferior survival outcomes in younger cohorts, an effect compounded by secondary malignancy risks. Analysis of 579,608 SEER patients (1973–2013) undergoing RP (*n* = 282,683) or radiotherapy (*n* = 296,925) revealed pronounced age‐stratified hazards [196]: For patients aged ≤55 years, EBRT, BT, and combination therapy conferred elevated OM HR of 2.10 (95% CI 1.62–2.70), 1.59 (1.12–2.26), and 1.55 (1.06–2.28) versus RP, respectively. Notably, these survival disparities attenuated with age, becoming nonsignificant in patients aged ≥75 years. Adding to the risk profile, younger radiotherapy recipients faced increased secondary bladder cancer (HR 2.36, 95% CI 1.82–3.06) and rectal cancer risks (HR 1.42, 95% CI 1.04–1.93), with corresponding mortality increases (cancer‐specific death HR 2.34, 95% CI 1.30–4.20; all‐cause HR 1.88, 95% CI 1.25–2.81). Mirroring the survival trends, secondary malignancy hazards diminished progressively with advancing age.

### 9.5 Systemic Treatment

Dedicated clinical studies investigating the efficacy of systemic therapies specifically in patients with EOPC are currently lacking. Based on our preceding summary of the molecular pathology of EOPC, in which TMPRSS2:ERG gene fusions serve as a molecular hallmark, we here infer the potential therapeutic responses of EOPC patients by examining how TMPRSS2:ERG fusion status modulates treatment sensitivity across various systemic modalities. It must be emphasized that this represents an indirect inference and a provisional prediction; true treatment responses in EOPC patients require rigorous, well‐designed clinical studies for confirmation.

#### 9.5.1 Endocrine Therapy

Current molecular pathology studies have established TMPRSS2:ERG gene fusion as a molecular hallmark of EOPC, raising the possibility that EOPC patients may exhibit enhanced sensitivity to ADT‐based endocrine therapy. However, clinical evidence does not uniformly support this hypothesis. A study of 239 patients with localized prostate cancer, in which TMPRSS2:ERG fusion status was determined by immunohistochemistry, found no significant difference between fusion‐positive and fusion‐negative patients in either OM (ERG+ vs. ERG−, HR 0.76, 95% CI 0.40–1.45) or PCSM (HR 0.89, 95% CI 0.60–1.34) following ADT [197]. Similarly, a prospective, open‐label, multicenter, single‐arm phase III study reported that urinary TMPRSS2–ERG scores did not appear useful for assessing response to ADT in advanced prostate cancer [198].

In the CRPC setting, neither TMPRSS2:ERG fusion detected in circulating tumor cells (CTCs) nor fusion status assessed by FISH in tumor tissue predicted sensitivity to enzalutamide or abiraterone [199, 200, 201]. However, analysis of the COU‐AA‐302 trial revealed heterogeneity among ERG‐rearranged cancers: tumors harboring an ERG fusion secondary to deletion of 21q22 accompanied by increased copy number of fusion sequences (Class 2+ Edel) derived greater improvement in rPFS from abiraterone acetate plus prednisone compared with fusion‐negative cancers (22 vs. 5.4 months; HR 0.31, 95% CI 0.15–0.68), whereas cancers with no ERG fusion showed a more modest benefit (16.7 vs. 8.3 months; HR 0.53, 95% CI 0.38–0.74) [202].

The only study to date specifically examining treatment responses in EOPC patients after progression to mCRPC utilized the prospectively collected, multisite electronic Prostate Cancer Australian Database [203]. This analysis included 915 mCRPC patients enrolled between June 2016 and March 2024, comparing outcomes between those with an initial diagnosis age <55 years (*n* = 59) and ≥55 years (*n* = 856). Results indicated that younger patients received significantly more lines of therapy beyond three compared with older patients (37 vs. 23%, *p* = 0.016). Median OS following first‐line systemic therapy was marginally longer in the younger cohort (41.9 vs. 35.1 months; HR 0.73, 95% CI 0.47–1.15). Multivariable analysis revealed that initial diagnosis age <55 years was not an independent prognostic factor for mCRPC (HR 0.82, 95% CI 0.52–1.29). However, these findings should be interpreted with caution given the limited sample size of the younger cohort (*n* = 59). In light of the established tumor biological differences between EOPC and SOPC, these data suggest that the response of EOPC patients progressing to mCRPC to existing first‐line therapies may not fully align with that observed in SOPC. Further investigation is warranted to elucidate these differences and establish comprehensive treatment paradigms tailored to EOPC.

#### 9.5.2 Chemotherapy

Analysis of the GETUG 15 trial, which evaluated ERG fusion status in relation to chemotherapy response in mHSPC, demonstrated that ERG expression may predict differential benefit from docetaxel [204]. In the ERG‐positive subgroup, median PFS was 10.7 months with ADT alone versus 18.8 months with ADT plus docetaxel. In the ERG‐negative subgroup, median PFS was 10.6 months with ADT alone versus 13.2 months with ADT plus docetaxel. These findings suggest that ERG expression predicts enhanced response to docetaxel in both high‐risk localized and metastatic hormone‐sensitive prostate cancer.

Conversely, studies in the mCRPC setting have associated TMPRSS2:ERG fusion with inferior outcomes following taxane chemotherapy. Reig and colleagues examined TMPRSS2–ERG detection in CTCs after chemotherapy and found that fusion detection correlated with lower PSA response rates (12.5 vs. 68.3%, *p* = 0.005), shorter PSA–PFS (PSA–PFS; 3.1 months vs. 7.5 months, *p* < 0.001), and shorter rPFS (3.1 vs. 8.2 months, *p* < 0.001) [205]. TMPRSS2–ERG status was independently associated with PSA–PFS (HR 3.7, *p* = 0.009) and rPFS (HR 6.3, *p* < 0.001). Moreover, TMPRSS2–ERG also predicted poor PSA–PFS in response to cabazitaxel. Another study assessing prechemotherapy ERG status reported that both blood‐based and tumor‐based TMPRSS2–ERG independently predicted shorter PSA–PFS (HR 3.3, 95% CI 1.4–7.9 and HR 1.8, 95% CI 1.02–3.3, respectively) following taxane treatment [206]. Collectively, these data indicate that for mCRPC, ERG‐positive prostate cancer patients exhibit significantly poorer responses to both docetaxel and cabazitaxel, as assessed by pretreatment tissue or CTC status and posttreatment CTC analysis.

Granted, these studies examined the association between ERG fusion status and chemotherapy sensitivity specifically in prostate cancer patients at particular disease stages. Whether this association persists in EOPC patients after progression to castration‐resistant disease requires further investigation.

#### 9.5.3 PARP Inhibitors

Evidence regarding the relationship between TMPRSS2:ERG fusion status and response to PARP inhibitors (PARPi) derives primarily from case series and mechanistic studies. A case report described six patients with HRR alterations. Among them, four patients, including two harboring concurrent TMPRSS2:ERG fusion and homozygous ATM or BRCA2 alterations, and two with heterozygous BRCA1/BRCA2 alterations in the absence of TMPRSS2:ERG fusion—derived no clinical benefit from PARP inhibition (treatment duration <16 weeks). In contrast, two patients lacking TMPRSS2:ERG fusion but carrying homozygous deleterious ATM or BRCA2 alterations both experienced clinical benefit (treatment duration ≥16 weeks) [207]. Mechanistic investigations revealed that ERG‐positive tumors shift DNA double‐strand break repair toward the PARP1‐dependent end‐joining (PARP1‐EJ) pathway, with elevated levels of PARP1, XRCC1, and LIG3 observed in ERG‐positive cells [208]. Notably, radiotherapy was shown to sensitize ERG‐positive cells to PARP inhibition, suggesting that the combination of radiotherapy and PARPi may represent a potential therapeutic strategy for EOPC patients.

#### 9.5.4 AKT Inhibitors

Mechanistic studies have identified a differential response to AKT pathway activation based on ERG fusion status. In ERG‐positive prostate cancer cells, ADT treatment significantly activates AKT, a phenomenon not observed in ERG‐negative cells, suggesting that the combination of androgen receptor pathway inhibition (ARPI) and AKT inhibition may be an effective therapeutic strategy for ERG‐positive prostate cancer [209]. However, the presence of PTEN loss modifies this relationship. In tumors with PTEN loss, concurrent ERG positivity confers resistance to combined ARPI and AKT inhibition, as ERG‐positive tumors maintain activation of AR lineage‐associated genes even when AR signaling is suppressed [210]. Thus, in the absence of PTEN loss, ERG positivity may serve as a predictive marker of benefit from combined ARPI and AKT inhibition, whereas in PTEN‐deficient tumors, ERG‐negative status is more likely to predict response. Notably, the proportion of PTEN loss among ERG‐positive patients decreases with advancing age (Figure 3A), further suggesting that this combination treatment strategy may be particularly relevant for younger EOPC patients. Future studies will require refined molecular stratification to identify those patients most likely to derive benefit from this therapeutic approach.

## 10 Key Differences From SOPC

EOPC and SOPC differ substantially in their risk factor profiles, and these differences lead in turn to divergent molecular features, signaling pathway dependencies, clinical behaviors, and treatment responses.

Regarding genetic factors, EOPC shows a stronger background of inherited susceptibility. It harbors unique germline mutations (e.g., PRUNE2, PYY, SERPINA3) distinct from those in SOPC, with HOXB13 G48E and HRR pathway mutations being more prominent in EOPC. PRSs also indicate a greater role of germline alterations in younger patients, and family history is particularly strongly associated with EOPC. However, genetics alone does not fully explain disease occurrence. Racial differences further suggest an interaction between genetic predisposition and early‐life environmental exposures: Black men have a higher risk of both EOPC and SOPC (earlier onset and faster progression), yet their outcomes diverge—survival in EOPC is similar between Black and White men, whereas in SOPC, Black men have substantially higher mortality than White men, implying distinct disease drivers in younger patients.

Turning to other modifiable risk factors, differences across socioeconomic, clinical, and disease‐related domains collectively point to the key role of early‐life exposures in EOPC pathogenesis. From a socioeconomic perspective, these factors more strongly affect access to early detection and timely diagnosis in younger populations, thereby shaping disease presentation. Regarding clinical measures, in‐utero exposure to androgens and raised serum free androgen levels are significantly associated only with aggressive EOPC, not with aggressive SOPC. Vitamin D deficiency increases EOPC risk but has no substantial association with SOPC. Lp(a)‐mediated pathways are involved in prostate carcinogenesis, and PCSK9 modulation shows a greater protective effect for early‐onset disease. SGLT2 inhibition significantly reduces the risk of both overall prostate cancer and EOPC, with a more pronounced effect on EOPC. By contrast, glycemic parameters and HbA1c have no substantive influence on prostate cancer development in men, and serum IGFs and related proteins do not appear biologically relevant to EOPC pathogenesis but may modify SOPC progression. From the perspective of clinical diseases, sleep disorders, IVF, and ICSI all have a greater effect on EOPC risk than on SOPC risk, further reinforcing the importance of early‐life exposures. Prostatitis might increase EOPC risk but could be a protective factor for SOPC. The patterns observed with early‐onset colon cancer also disproportionately elevate EOPC risk (independent of radiotherapy), with attenuation at older ages; the short‐term postdiagnosis risk spike suggests synchronous detection bias, and the age‐risk gradient may reflect shared driver mutations in early‐onset malignancies. These systematic differences in risk factors inevitably lead EOPC and SOPC down distinct molecular paths.

At the molecular level, prostate cancer is characterized by copy number alterations and SRs rather than recurrent point mutations. EOPC has a significantly lower overall mutational burden (including SNVs and SRs) than SOPC, but the proportion of SRs among all mutations is significantly higher in EOPC. Moreover, SRs in EOPC predominantly occur in open chromatin regions, active enhancer elements, and transcription factor binding sites, whereas SRs in SOPC are more frequently localized to closed, inactive chromatin regions. EOPC is dominated by TMPRSS2:ERG fusions, underscoring the central role of AR signaling in its pathogenesis; other progression‑promoting genes, such as ESRP1, are independent of SOPC. By contrast, the common driver genes in SOPC (e.g., NCOR2, CHD1, MAP3K7, PTEN) show significantly different mutational burdens in EOPC. Owing to these differences in risk factors and somatic mutations, the signaling pathways on which the two diseases depend are also markedly different. EOPC exhibits abnormal AR and cell‑cycle signaling, accompanied by secondary abnormalities in HER2/EGFR, hypoxia, and fatty acid metabolism, together with a microenvironment characterized by APOE^+^ macrophages, increased inhibitory CD4^+^ T cells, and decreased stimulatory CD8^+^ T cells. In contrast, SOPC differs fundamentally from the AR‑centric model of EOPC: SOPC tumor cells show EMT signals driven by interactions with inflammatory cancer‑associated fibroblasts (iCAF subtype APOD^+^/CCL2^+^), activating SMAD pathways via BMP/BMPR signaling.

These molecular and pathway differences ultimately manifest in clinical features and treatment responses. Patients with EOPC have larger prostate volumes but smaller tumor volumes, and their age‑adjusted PSA levels show a greater elevation, further supporting an AR‑driven mechanism. Although the distributions of Gleason grade and TNM stage are generally similar between EOPC and SOPC, survival outcomes diverge by risk category: for low‑risk tumors, survival is similar between the two groups, but for high‑risk tumors, EOPC has significantly worse survival than SOPC. This finding suggests that current clinical staging systems (GS, TNM) might not be directly applicable to EOPC. Regarding treatment response, patients with localized EOPC have significantly better outcomes after RP in terms of efficacy and postoperative recovery of urinary continence and sexual function, highlighting the clinical value of early detection of clinically meaningful EOPC.

## 11 Early Detection and Prevention of EOPC

Research conducted over recent decades has established the following key points.

**FIGURE 5:**
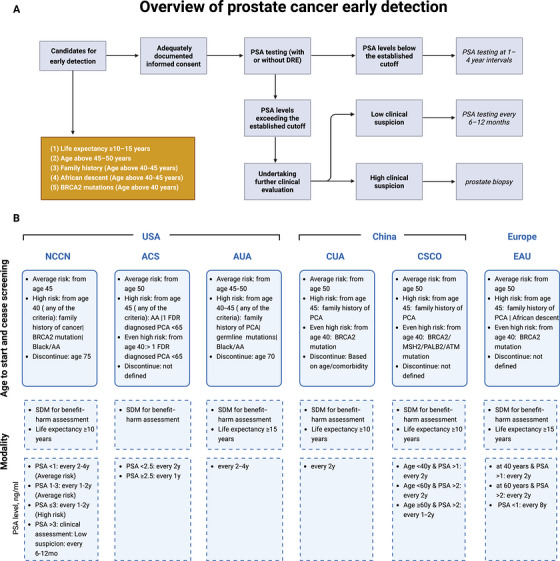
Current frameworks for prostate cancer early detection. (A) Guideline‐recommended early detection pathways; (B) comparative recommendations in US, Chinese, and European clinical practice guidelines. *Abbreviations*: NCCN, National Comprehensive Cancer Network; ACS, American Cancer Society; AUA, American Urological Association; CUA, Chinese Urological Association; CSCO, Chinese Society of Clinical Oncology; EAU, European Association of Urology.

1. Population‐wide PSA‐based prostate cancer screening is not recommended: For the general population, studies including the Prostate, Lung, Colorectal, and Ovarian (PLCO) Cancer Screening Trial and the European Randomized Study of Screening for Prostate Cancer (ERSPC) indicate that regular PSA testing (every 1–4 years) in men aged 55–74 years can significantly reduce PCSM by 20–32% compared with no PSA testing [211, 212, 213, 214, 215, 216]. The Swiss cohort of ERSPC (the Göteborg‐1 cohort, men aged 50–64 years, biennial PSA testing) demonstrated an even greater reduction of 41% in PCSM [217, 218]. Conversely, the Cluster randomized trial of PSA testing for prostate cancer (CAP) showed that a single PSA test did not confer a significant survival benefit (RR 0.96, 95% CI 0.85–1.08]) [219], further underscoring the importance of regular PSA testing for early prostate cancer detection. Given that the natural history of prostate cancer is generally indolent, population‐based PSA screening leads to substantial overdiagnosis and overtreatment. The number needed to invite for screening to diagnose one patient with prostate cancer was 26 (95% CI 24–29) in ERSPC, 84 (95% CI 59–144) in the PLCO trial, and 154 (95% CI 128–192) in CAP [212, 216, 219, 220, 221, 222, 223]. Real‐world studies corroborate this burden, with the number needed to diagnose and number needed to treat for the whole population estimated at 11–14 and 7–11, respectively (SEER data up to 2016) [224].
2. Contemporary clinical practice guidelines endorse risk‐adapted PSA testing (Figure 5), followed by appropriate investigations such as multiparametric magnetic resonance imaging (mpMRI), for men after thorough shared decision‐making regarding benefits and harms [225, 226, 227, 228, 229, 230]. This approach aims to reduce unnecessary biopsies while enhancing the detection of clinically significant prostate cancer (csPCa). Although definitive evidence demonstrating that this strategy significantly reduces overdiagnosis and overtreatment while yielding substantial PCSM benefits is still awaited, initiatives like the PRAISE‐U project are actively promoting the early detection and diagnosis of prostate cancer through customized, risk‐adapted early detection programs [231].
3. Discrepancies exist in screening initiation age recommendations. While current guidelines suggest initiating screening at the age of 45–50 years for average‐risk individuals and 40–45 years for high‐risk individuals, the 2018 US Preventive Services Task Force (USPSTF) guideline recommends shared decision‐making regarding prostate cancer screening only for men aged 55–69 years [232]. Notably, the USPSTF statement provides no specific guidance for individuals under 55 years.

**FIGURE 6:**
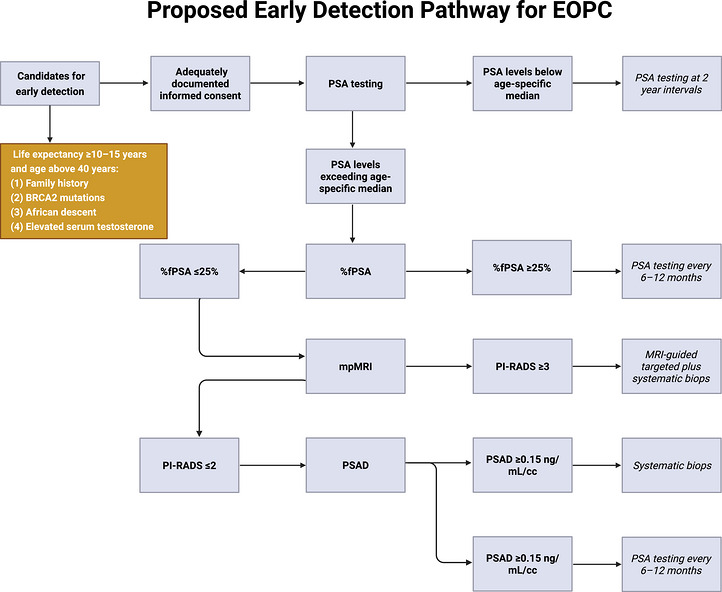
Risk‐adapted early detection pathway for early‐onset prostate cancer. Based on current evidence and established screening models for prostate cancer, we propose a preliminary risk‐stratified pathway for the early detection of EOPC, incorporating risk factor assessment, PSA testing, percentage free PSA (%fPSA), and multiparametric magnetic resonance imaging of the prostate.

Integrating current guidelines with the distinctive features of EOPC discussed earlier, we propose the following steps for the early detection of clinically significant EOPC to mitigate its disease burden (Figure 6). (It is crucial to emphasize that the efficacy of this approach in significantly enhancing csEOPC detection without increasing overdiagnosis and overtreatment, ultimately reducing PCSM in this population, requires validation through future RCTs.)

### 11.1 Communication and Individual Selection

Similar to early detection strategies for general prostate cancer, thorough communication regarding the potential risks and benefits of early detection measures and their outcomes is essential before initiating any evaluation for an individual. Subsequently, men aged ≥40 years with a life expectancy exceeding 10–15 years should undergo comprehensive risk factor assessment. Individuals meeting any of the following criteria are considered at high risk for EOPC. (These considerations may be especially important in younger patients, for whom the balance between early detection and overdiagnosis differs from that in older individuals.)

(1) Positive family history of prostate cancer: Focus specifically on the number of FDRs affected and the age at which the father was diagnosed.

(2) Family history suggestive of hereditary cancer syndromes: While the status of pathogenic germline mutations is often unknown, alongside prostate cancer family history, particular attention should be paid to personal or family history of breast, ovarian, or pancreatic cancer (especially with early onset or aggressive disease), as well as syndromes such as Li–Fraumeni, Cowden, PTEN hamartoma tumor syndrome, Lynch syndrome, or Ashkenazi Jewish ancestry [233]. For individuals with these risk factors, comprehensive counseling and germline mutation testing (particularly for BRCA2 pathogenic variants) are recommended.

(3) African ancestry or Black race.

(4) Elevated serum testosterone (consideration): Evidence linking lifestyle or environmental factors to EOPC risk remains low‐grade; hence, these factors cannot currently guide decisions for EOPC early detection. However, based on evidence suggesting EOPC's heightened dependence on the androgen receptor pathway and MR studies indicating a significant association between higher free testosterone levels and aggressive EOPC risk, baseline serum total and free testosterone measurement could be considered for men ≥40 years with >10–15 years life expectancy. For individuals with elevated levels, early EOPC detection might be considered after careful benefit–harm assessment.

### 11.2 PSA Testing

Based on current guidelines and practice, individuals identified with the aforementioned EOPC high‐risk factors should initiate regular PSA testing. While PSA testing for early detection is established, optimal PSA cut‐off values for triggering further investigation and the ideal testing interval remain unresolved. Current evidence clearly highlights the prognostic value of baseline PSA and age at first testing.

1. Men with a baseline PSA <1 ng/mL at age 40 or <2 ng/mL at the age of 60 years exhibit a substantially decreased risk of prostate cancer metastasis or death decades later [234, 235]. Although prospective data for men <55 years are limited, the PLCO study showed csPCa incidence rates over 13 years of 1.5 and 5.4% for men aged 55–60 years with PSA <1 and 1–2 ng/mL, respectively [236].
2. The Göteborg‐1 cohort identified prognostic factors associated with increased risk of PCa death: nonattendance to PSA screening, initiating screening after the age of 60 years, and stopping screening at the age of 70 years [237].

Regarding PSA cut‐offs, several guidelines (e.g., NCCN, EAU, CSCO) recommend age‐specific PSA thresholds for risk assessment (Figure 5). The normal reference range for PSA increases with age. For men aged 40–49 years, median PSA values range from 0.5 to 0.7 ng/mL, and the 75th percentile values range from 0.7 to 0.9 ng/mL [228]. Crucially, individuals with a PSA level above the median for their age group face a higher risk of prostate cancer and aggressive disease; the risk escalates with increasing PSA above the median [228]. Therefore, for early detection of EOPC in high‐risk individuals, we favor the NCCN‐recommended strategy: young individuals with PSA levels exceeding their age‐specific median, even if below the traditional >3 ng/mL threshold, should proceed to further evaluation. Conversely, individuals with PSA below their age‐specific median may undergo biennial PSA testing.

### 11.3 Further Evaluation

The PLCO and ERSPC trials revealed negative biopsy rates of 67.7 and 75.8% [238, 239], respectively, following biopsies that are triggered solely by exceeding PSA thresholds, thereby exposing individuals to risks such as infection. Furthermore, the majority of biopsy‐positive cases represented low‐risk cancer (unlikely to impact survival if undetected, while biopsy and treatment may adversely affect quality of life). Risk‐adapted early detection strategies therefore do not advocate immediate biopsy for all exceeding a predefined PSA threshold. Instead, they utilize risk calculators, mpMRI, and other tools to triage individuals requiring biopsy, aiming to reduce unnecessary procedures while enhancing csPCa detection [225, 228, 240]. Considering the lower absolute incidence but potentially higher grade and greater lifetime risk in young men with EOPC, coupled with their longer life expectancy, detailed further evaluation is paramount for this group. Based on current evidence and guidelines, we propose the following sequential steps for young men with elevated PSA (acknowledging limited direct evidence on sequence).

1. %fPSA: Numerous studies demonstrate significantly lower %fPSA in prostate cancer patients versus benign cases [228]. FDA‐approved for men >50 years with PSA 4–10 ng/mL and normal DRE, multicenter studies show a 25% cutoff detects 95% of cancers while avoiding 20% unnecessary biopsies [241]. Population‐based evidence confirms %fPSA lacks age correlation in healthy men [242, 243]. Thus, for young men with PSA > age‐specific median, we recommend %fPSA calculation. Those with %fPSA < 25% should proceed to next‐step evaluation, while others may follow NCCN‐recommended 6–12 month PSA monitoring.
2. mpMRI: Robust RCT and real‐world data indicate mpMRI (using PI‐RADS ≤ 2) avoids 21–49% unnecessary biopsies [244, 245, 246, 247, 248, 249, 250]. Caution remains warranted as 2–14% risk of significant cancer persists in this group; systematic biopsy alone may miss significant cancers while increasing indolent cancer detection [245, 251]. NCCN strongly recommends mpMRI for elevated PSA, with MRI‐targeted plus systematic biopsy for PI‐RADS ≥ 3 lesions. Therefore, young men with %fPSA < 25% should undergo mpMRI. Those with PI‐RADS ≤ 2 should proceed to PSA density (PSAD) assessment, while PI‐RADS ≥ 3 warrants MRI‐guided targeted plus systematic biopsy.
3. PSAD: PSAD requires prostate volume measurement via imaging (PSA [ng/mL]/volume [cc]) [252, 253]. Lower ratios suggest PSA elevation is likelier from prostate enlargement (BPH). Studies indicate a 0.15 ng/mL/cc cutoff avoids 50% unnecessary biopsies, though recent reports note its limited sensitivity [254]. Contemporary evidence links PSAD to cancer aggressiveness, adverse pathology, and posttreatment BCR [255, 256]. Crucially, PSAD correlates with clinically significant prostate cancer only in small/medium prostates (*p* = 0.03; *p* = 0.01), not large glands (*p* = 0.36), with sensitivity for significant cancer highest in small prostates (72.7%) versus medium (43%) or large glands (3.2%) [257]. Given EOPC's smaller prostate volume versus SOPC and greater consequences of missing high‐grade disease, we emphasize PSAD assessment for mpMRI‐negative young men. Those with PSAD < 0.15 ng/mL/cc should undergo 6–12 month PSA monitoring, while PSAD ≥0.15 ng/mL/cc warrants systematic biopsy.

### 11.4 Other Biomarkers

(1) Prostate Health Index (PHI): The PHI, a composite score derived from tPSA, fPSA, and the [−2]proPSA isoform, has emerged as a promising tool for prostate cancer detection [258, 259]. In a multicenter study, PHI demonstrated approximately twice the sensitivity of %fPSA for cancer detection in men with serum PSA concentrations between 2 and 10 ng/mL [260]. Furthermore, PHI correlates with tumor grade, achieving an area under the curve (AUC) of 0.72 for discriminating GG ≥ 2 cancer from low‐grade cancer or negative biopsy, with another study reporting an AUC as high as 0.815 for detecting clinically significant prostate cancer [261]. This prospective investigation also identified an optimal PHI cutoff of 24, which would have avoided 36% of biopsies while missing approximately 2.5% of high‐grade cancers [261]. Based on this evidence, the US Food and Drug Administration approved PHI in 2012 for use in men with serum PSA values between 4 and 10 ng/mL.

Extending these findings to younger populations, a nested case–control study within the PRO‐PSA Multicentric European Study (PROMEtheuS) project specifically analyzed 238 men under 60 years of age [262]. In this cohort, PHI predicted prostate cancer at biopsy with an AUC of 0.700, significantly outperforming tPSA (AUC 0.549), fPSA (AUC 0.511), and %fPSA (AUC 0.557) (*p* ≤ 0.001 for all comparisons). These data suggest that PHI may serve as a useful biomarker for early detection of prostate cancer in younger men, including those at risk for EOPC. However, its ability to specifically identify clinically significant disease in this age group remains uncertain; some studies have raised concerns that PHI might miss up to 30% of clinically significant cancers [263]. Therefore, while PHI represents a promising adjunctive tool for early detection in younger populations, it should be applied judiciously and interpreted within the context of other clinical findings.

(2) 4Kscore: The 4Kscore test represents another multimarker panel that measures four kallikrein markers, fPSA, tPSA, intact PSA, and human kallikrein 2 (hK2), and integrates them with clinical variables including age, DRE findings, and prior biopsy status [264, 265]. Numerous retrospective studies involving large cohorts have demonstrated that the 4Kscore achieves high discriminatory accuracy for distinguishing GG ≥ 2 tumors from benign findings or GG 1 disease, with AUC values typically exceeding 0.80 [266, 267, 268, 269]. These investigations have also shown that incorporation of the 4Kscore could reduce unnecessary biopsies by more than 40%. A multicenter study reported an even greater potential benefit, with up to 65% of unnecessary biopsies potentially avoidable, and observed a significant correlation between 4Kscore risk categories and Gleason grade (*p* < 0.01) [270]. Based on this body of evidence, the FDA approved the 4Kscore in 2021 to aid in the detection of aggressive prostate cancer in patients for whom a biopsy is being considered.

When evaluated specifically in younger populations, the 4Kscore has shown particular promise. A prospective study of men aged 50 years without prior PSA screening who presented with moderately elevated PSA levels (≥2.0 ng/mL) found that the 4Kscore markedly improved risk discrimination, increasing the C‐index from 0.767 to 0.828 [271]. Further supportive evidence comes from a nested case–control study within the European Prospective Investigation into Cancer and Nutrition, which analyzed baseline blood samples from 1658 men subsequently diagnosed with prostate cancer (median time to diagnosis: 8.6 years) and 1658 matched controls [272]. Among men with PSA > 2 ng/mL, the 4Kscore demonstrated better discriminatory performance than PSA alone for predicting overall prostate cancer, high‐grade disease, and prostate cancer death, but this advantage was observed only in men younger than 60 years at recruitment. Collectively, current evidence suggests that the 4Kscore does not substantially improve prediction of clinically significant prostate cancer compared with total PSA alone, except in younger men with elevated PSA levels. However, it should be noted that some studies have reported that use of the 4Kscore may lead to 10–20% of GG ≥ 2 prostate cancers being missed [266, 267, 268, 269]. The value of the 4Kscore specifically for early detection of EOPC remains to be established through large‐scale prospective studies.

(3) ExoDx Prostate (IntelliScore): ExoDx Prostate (IntelliScore), also referred to as EPI, is a urine‐based gene expression assay that analyses exosomal RNA derived from PCA3 and ERG, normalized to SPDEF [273]. This three‐gene panel is designed to discriminate GG ≥ 2 prostate cancer from GG 1 disease or benign findings at initial biopsy. The intended population for which the assay was initially developed includes patients over 50 years of age with no prior biopsy and PSA values between 2 and 10 ng/mL.

In a validation cohort, the assay demonstrated an AUC of 0.71 for detecting GG ≥ 2 prostate cancer, with significant improvement in predictive accuracy when added to standard‐of‐care variables compared with standard variables alone [274]. Application of the test would have avoided 27% of unnecessary biopsies while missing only 8% of GG ≥ 2 cancers. Preliminary findings from a two‐phase adaptive clinical utility study reported similar results, with the second phase of this trial expected to be reported in the future [275]. Another clinical utility study encompassing 1094 patients found that ExoDx Prostate increased the detection rate of GG ≥ 2 cancers by 30% [276].

However, a prospective, blinded, randomized, multicenter clinical utility study involving 1049 men aged 50 years or older with PSA levels between 2 and 10 ng/mL who were being considered for prostate biopsy yielded less favorable results [277]. In this trial, use of ExoDx Prostate led to a 13% increase in the number of prostate biopsies performed, yet only modestly improved the detection rate of GG ≥ 2 cancers from 20.6 to 24.8%, while concurrently increasing the diagnosis of GG 1 disease by 5.1%.

Given these conflicting findings, the value of ExoDx Prostate specifically for early detection of EOPC remains uncertain. Current evidence suggests that its application may carry a substantial risk of increasing unnecessary biopsies and overdiagnosis, without definitively enhancing the detection of clinically significant EOPC. Definitive conclusions regarding its clinical utility will require the final results of ongoing trials, as well as future studies specifically designed to evaluate its performance in younger populations at risk for EOPC.

(4) MyProstateScore: The MyProstateScore (MPS) assay combines total serum PSA with post‐DRE urinary measurements of PCA3 and the TMPRSS2:ERG fusion gene transcript [278]. To validate its utility for early prostate cancer detection, a study involving 1244 individuals demonstrated that MPS achieved an AUC of 0.75 for predicting any cancer, compared with 0.59 for PSA alone [278]. For predicting GG ≥ 2 cancer, the AUCs for MPS and PSA alone were 0.77 and 0.65, respectively. Another study comprising 1525 men reported that incorporation of MPS could reduce unnecessary biopsies by 33% while missing only 3% of GG ≥ 2 cancers [279].

Building upon this platform, the MPS 2.0 assay (MPS2) was subsequently developed to enhance diagnostic accuracy for clinically significant prostate cancer in individuals with elevated PSA levels [280]. MPS2 measures 18 markers that are selectively overexpressed in GG ≥2 tumors compared with GG 1 disease [280]. Analysis of post‐DRE urine samples from 761 individuals with elevated PSA levels prior to biopsy revealed that MPS2 achieved an AUC of 0.82 for detecting GG ≥2 cancer, significantly outperforming the original MPS assay (AUC 0.74) [281]. Moreover, MPS2 would have reduced unnecessary biopsies by 37%. Collectively, these findings underscore the substantial potential of both MPS and MPS2 in reducing unnecessary biopsies while maintaining high sensitivity for clinically significant prostate cancer in early detection settings.

Notably, although TMPRSS2:ERG fusion occurs at substantially higher frequencies in EOPC than in SOPC, the studies validating MPS and MPS2 have predominantly enrolled patients with standard‐onset disease. Whether these assays retain comparable diagnostic value specifically for early detection of EOPC remains to be established through dedicated investigations in younger populations.

## 12 Critical Appraisal of Current Evidence

### 12.1 Methodological Limitations of Current Evidence

It should be noted that, despite our comprehensive synthesis of risk factors, tumor biology/molecular pathology, clinicopathological characteristics, survival outcomes, and treatment modalities for EOPC, research in this field remains in its infancy. The overall quality of the available evidence is limited, and several critical gaps and methodological shortcomings warrant consideration.

Regarding studies on risk factors for EOPC, no investigation to date has been specifically designed to address this question. Nearly all putative risk factors have been derived from subgroup analyses by age within broader prostate cancer cohorts, which inherently compromises statistical power and diminishes their clinical interpretability. Second, virtually all studies, particularly those investigating germline mutations, family history, and race, have conflated hereditary/familial EOPC with sporadic cases, precluding the identification of risk factor profiles unique to each subtype and hindering the development of precision prevention strategies. Third, the overwhelming majority of studies on modifiable risk factors employ case–control designs, which can only establish associations rather than causality. It remains unclear whether the identified factors truly alter EOPC risk or merely represent incidental correlates. Although some associations have been corroborated by MR studies, long‑term prospective cohort data remain lacking. Finally, most included cohorts are predominantly of European ancestry, leaving it uncertain whether these putative risk factors generalize to other racial and ethnic populations.

Similar limitations apply to studies on tumor biology and molecular pathology. Although these investigations have exclusively enrolled EOPC patients, they have not systematically ascertained family history or germline mutation status. Consequently, the reported findings reflect the characteristics of EOPC as a whole, without delineating the distinct molecular landscapes of sporadic versus hereditary/familial EOPC. Such distinctions are critical for guiding subsequent systemic therapy and precision medicine. Furthermore, the limited available studies have focused almost exclusively on tumor cells themselves; only one multiomics investigation has examined differences in the TME, including immune and stromal cell populations, between EOPC and SOPC. Given that the tumor functions as an integrated ecosystem, interactions among immune cells, stromal components, and malignant cells profoundly influence disease progression and therapeutic response. Last, no study has characterized the evolution of tumor cells or the TME following systemic therapies such as ADT in EOPC patients. This knowledge gap poses substantial challenges for selecting appropriate treatments when EOPC patients progress to castration‐resistant disease.

### 12.2 Knowledge Gaps and Definitional Heterogeneity

Research on clinicopathological characteristics, survival outcomes, and treatment responses in EOPC is even more limited, with these areas remaining virtually uncharted. First, all available studies are retrospective in design, and the majority rely on analysis of existing databases. Second, there are essentially no studies specifically designed to evaluate treatment responses in EOPC patients. The current understanding of EOPC outcomes following RP, RT, and other modalities is derived almost exclusively from age‐based subgroup analyses within broader cohorts. The statistical reliability of these analyses requires careful assessment by clinicians, and the potential for contradictory findings cannot be overlooked. Finally, no studies have investigated whether EOPC patients who progress to mHSPC or mCRPC exhibit differential responses to standard‐of‐care therapies established for SOPC. Even age‐stratified subgroup analyses are lacking in this context.

Furthermore, although we adopted <55 years as the age threshold for defining EOPC in this review, not all included studies used this specific cutoff. For studies in which the original age cutoff was below 55 years, we uniformly categorized them as EOPC without further stratification. In addition, some studies with an age cutoff of 60 years were included because they provided age‐stratified trend analyses, which may offer indirect insights into how age modulates the factors under investigation. Accordingly, several caveats should be considered when interpreting the evidence synthesized in this review: (1) Prostate cancers diagnosed before age 55 years may be biologically distinct from those diagnosed at even younger ages (e.g., <50 or <45 years), and their respective risk factor profiles and treatment responses may also differ substantially. However, given the paucity of EOPC‐specific studies, precise stratification by finer age categories is not currently feasible. (2) Conclusions derived solely from age‐trend analyses should be regarded as preliminary, as it remains uncertain whether the observed patterns specifically apply to EOPC patients. Overall, research on EOPC remains in a very early exploratory phase, and well‐powered, dedicated studies are urgently needed to address these knowledge gaps.

## 13 Future Directions

### 13.1 EOPC as a Distinct Entity: Burden, Definitions, and Foundational Premises

The rising global burden of EOPC is underscored by its persistently increasing incidence and a projected mortality growth rate substantially exceeding incidence trends. This finding indicates a rapidly escalating disease burden that warrants urgent attention. However, research on EOPC remains nascent, necessitating significantly heightened scientific focus in the coming decades.

Current evidence suggests three foundational premises: (1) Distinct disease entity: EOPC exhibits unique etiology and biological behavior compared with SOPC. (2) Comparable risk profiles: Clinicopathological prognostic factors in EOPC patients are not demonstrably more favorable than those in SOPC. (3) Survival disadvantage: Patients with high‐risk or de novo metastatic EOPC face significantly worse survival outcomes than their SOPC counterparts.

Establishing a precise EOPC definition is paramount. As discussed in Part II, the current lack of consensus on age thresholds (e.g., ≤50, ≤55, ≤60 years) leads to divergent research conclusions. Furthermore, chronological age inadequately reflects individual biological status. Emerging technologies enabling the assessment of biological age offer a promising avenue for defining EOPC based on intrinsic ageing processes. Such a definition could more accurately elucidate its biology and inform targeted management and early detection strategies. Additionally, while low‐risk EOPC generally shares the favorable prognosis of low‐risk SOPC, the extended life expectancy of younger patients introduces uncertainty regarding long‐term progression risks. Consequently, whether EOPC should be strictly defined as clinically significant prostate cancer warrants rigorous investigation.

### 13.2 Critical Research Gaps and Urgent Clinical Priorities

Etiological research faces critical limitations. Most studies analyze EOPC merely as a subgroup within broader SOPC cohorts. Dedicated investigations into EOPC‐specific genetic susceptibility and risk factors are scarce and predominantly retrospective case–control designs. Although family history is a significant EOPC risk factor, conflating all EOPC with hereditary or familial prostate cancer is erroneous. This conflation overlooks sporadic EOPC cases, constraining research validity. Prospective birth cohort studies are essential to establish causal relationships between risk factors and EOPC development—moving beyond current correlative evidence—and are fundamental for devising effective prevention strategies. Importantly, dominant risk factors evolve with societal changes across generations, making this a temporally evolving research priority.

Comprehensive longitudinal biobanking is indispensable for unraveling EOPC tumor biology and progression mechanisms. Critical elements include: (1) Systematically capturing precise age at diagnosis, risk factors (family history, ethnicity, germline mutation status, environmental exposures), clinicopathological stage, histology, treatments, and therapeutic responses. (2) Serial collection of tumor tissue, blood, urine, and other biofluids across disease stages. (3) Multiomic profiling (genomic, transcriptomic, metabolomic, epigenomic) integrated with clinical data. Leveraging artificial intelligence on these rich datasets can reveal stage‐specific biological behaviors, enable precise molecular subtyping, and identify novel therapeutic vulnerabilities.

Addressing clinical management gaps is urgent. Implementing the above research vision is long‐term. Currently, no EOPC‐specific management guidelines exist, and the applicability of SOPC‐derived treatments across identical disease stages in EOPC remains unexplored. Existing evidence indicates poorer outcomes for high‐risk or metastatic EOPC, raising critical questions: Does aggressive local therapy benefit these patients? What is the role and efficacy of adjuvant systemic therapies like ADT post‐local treatment? Critically, evidence is entirely lacking for managing EOPC progressing to metastatic castration‐resistant disease.

Generating robust clinical evidence will require considerable time. Immediate steps include: (1) Prioritizing collection of precise diagnostic age in all real‐world studies and clinical trials. (2) Conducting preplanned subgroup analyses based on diagnostic age strata. This will yield preliminary insights to guide practice while awaiting definitive evidence from dedicated EOPC trials. Concerted global effort and resource allocation are imperative to mitigate the growing burden of this distinct disease entity.

## 14 Conclusion

In summary, the incidence and mortality of EOPC are expected to continue rising in the future. On the basis of current evidence, EOPC can already be considered a distinct subtype of prostate cancer. It differs significantly from SOPC in terms of risk factors, pathogenesis, survival outcomes, and treatment response. Nevertheless, research on EOPC remains at a very preliminary stage. To date, no targeted early‐detection strategies for EOPC are available, investigations into its risk factors and pathogenesis are still limited, and there are almost no independent clinical studies focusing specifically on the treatment of EOPC. Future research should pay greater attention to the underlying mechanisms and biomarkers of EOPC, and should aim to establish standardized, disease‐specific diagnostic and therapeutic models for patients with EOPC.

## Author Contributions

XYX, LY, QW, and HX were responsible for the conception and design of the study. XYX, WZZ, WCH, and SYZ did the analysis and interpreted the analysis. XYX, WCH, JY, and QD were responsible for the acquisition of data. XYX, WZZ, and SYZ wrote the first draft of the manuscript. XYX interpreted the data and wrote the final version. All authors critically revised the article for important intellectual content and approved the final version. LY, QW, and HX obtained public funding.

## Funding

This work was supported by the National Natural Science Foundation of China (Grant No. 82372831, 82403963), Natural Science Foundation of Sichuan, China (2025ZNSFSC1892), National Key Research and Development Program (2024YFB3311703‐1), and the Postdoctor Research Fund of West China Hospital, Sichuan University (2025HXBH134).

## Ethics Statement

The authors have nothing to report.

## Conflicts of Interest

The authors declare no conflicts of interest.

## Data Availability

The authors have nothing to report.

## References

[1] H. Sung , J. Ferlay , R. L. Siegel , et al., “Global Cancer Statistics 2020: GLOBOCAN Estimates of Incidence and Mortality Worldwide for 36 Cancers in 185 Countries,” CA: A Cancer Journal for Clinicians 3 (2021): 209–249. 10.3322/caac.21660 33538338

[2] N. D. James , I. Tannock , J. N'Dow , et al., “The Lancet Commission on Prostate Cancer: Planning for the Surge in Cases,” Lancet (London, England) 10437 (2024): 1683–1722. 10.1016/S0140-6736(24)00651-2 PMC7617369 38583453

[3] D. Chakrabarti , P. Albertsen , A. Adkins , et al., “The Contemporary Management of Prostate Cancer,” CA: A Cancer Journal for Clinicians 6 (2025): 552–586. 10.3322/caac.70020 PMC12593286 40572035

[4] R. J. Rebello , C. Oing , K. E. Knudsen , et al., “Prostate Cancer,” Nature Reviews Disease Primers 7 (2021): 9. 10.1038/s41572-020-00243-0 33542230

[5] C. A. Salinas , A. Tsodikov , M. Ishak‐Howard , et al., “Prostate Cancer in Young Men: An Important Clinical Entity,” Nature Reviews Urology 6 (2014): 317–323. 10.1038/nrurol.2014.91 PMC4191828 24818853

[6] W. Lin , Y. Ou , H. Huang , et al., “Epidemiology of Early Onset Prostate Cancer: Global Patterns, Risk Factors, and Projections Through 2040,” Annals of Surgical Oncology (2025), Epub ahead of print. 10.1245/s10434-025-17665-3 40553361

[7] T. Ugai , N. Sasamoto , H. Y. Lee , et al., “Is Early‐Onset Cancer an Emerging Global Epidemic? Current Evidence and Future Implications,” Nature Reviews Clinical Oncology 10 (2022): 656–673. 10.1038/s41571-022-00672-8 PMC9509459 36068272

[8] N. Ribelles , J. Pascual , L. Galvez‐Carvajal , et al., “Is Early‐Onset Cancer an Emerging Global Epidemic? Current Evidence and Future Implications,” Nature Reviews Clinical Oncology 10 (2022): 656–673. 10.1038/s41571-022-00672-8 PMC9509459 36068272

[9] J. Zhao , L. Xu , J. Sun , et al., “Global Trends in Incidence, Death, Burden and Risk Factors of Early‐Onset Cancer From 1990 to 2019,” BMJ Oncology 2 (2023): e000049. 10.1136/bmjonc-2023-000049 PMC11235000 39886513

[10] S. G. Patel , J. J. Karlitz , T. Yen , et al., “The Rising Tide of Early‐Onset Colorectal Cancer: A Comprehensive Review of Epidemiology, Clinical Features, Biology, Risk Factors, Prevention, and Early Detection,” The Lancet Gastroenterology & Hepatology 3 (2022): 262–274. 10.1016/S2468-1253(21)00426-X 35090605

[11] Y. Ding , Y. Zuo , B. Zhang , et al., “Comprehensive Human Proteome Profiles Across a 50‐Year Lifespan Reveal Aging Trajectories and Signatures,” Cell 20 (2025): 5763–5784.e26. 10.1016/j.cell.2025.06.047 40713952

[12] J. Xiong , X. Zhu , Y. Guo , et al., “Multi‐Omic Underpinnings of Heterogeneous Aging Across Multiple Organ Systems,” Cell Genomics 12 (2025): 101032. 10.1016/j.xgen.2025.101032 PMC12802651 41043431

[13] T. Lu , Y. Xie , Y. Wang , et al., “Protein Restriction Reprograms the Multi‐Organ Proteomic Landscape of Mouse Aging,” Cell 25 (2025): 7309–7326.e20. 10.1016/j.cell.2025.10.004 41138729

[14] S. Hussein , S. Satturwar , and T. Van der Kwast , “Young‐Age Prostate Cancer,” Journal of Clinical Pathology 7 (2015): 511–515. 10.1136/jclinpath-2015-202993 25837493

[15] C. Gerhauser , F. Favero , T. Risch , et al., “Molecular Evolution of Early‐Onset Prostate Cancer Identifies Molecular Risk Markers and Clinical Trajectories,” Cancer Cell 6 (2018): 996–1011.e8. 10.1016/j.ccell.2018.10.016 PMC7444093 30537516

[16] Y. Cheng , B. Liu , J. Xin , et al., “Single‐Cell and Spatial RNA Sequencing Identify Divergent Microenvironments and Progression Signatures in Early‐ Versus Late‐Onset Prostate Cancer,” Nature Aging 5 (2025): 909–928. 40211000 10.1038/s43587-025-00842-0

[17] L. Montégut , C. López‐Otín , and G. Kroemer , “Aging and Cancer,” Molecular Cancer 23 (2024): 106. 38760832 10.1186/s12943-024-02020-z PMC11102267

[18] M. C. White , D. M. Holman , J. E. Boehm , et al., “Age and Cancer Risk: A Potentially Modifiable Relationship,” American Journal of Preventive Medicine 3 Suppl 1 (2014): S7–15. 10.1016/j.amepre.2013.10.029 PMC4544764 24512933

[19] M. R. Hamczyk , R. M. Nevado , A. Barettino , et al., “Biological Versus Chronological Aging: JACC Focus Seminar,” Journal of the American College of Cardiology 8 (2020): 919–930. 10.1016/j.jacc.2019.11.062 32130928

[20] J. L. Guida , L. Gallicchio , and P. A. Green , “Are Early‐Onset Cancers an Example of Accelerated Biological Aging?,” JAMA Oncology 7 (2025): 690–691. 10.1001/jamaoncol.2025.1143 40440036

[21] Y. Wu , Y. Jin , L. Deng , et al., “Long‐Term High‐Altitude Exposure, Accelerated Aging, and Multidimensional Aging‐Related Changes,” JAMA Network Open 8, no. 5 (2025): e259960. 40358947 10.1001/jamanetworkopen.2025.9960 PMC12076175

[22] F. Cui , L. Tang , D. Li , et al., “Early‐Life Exposure to Tobacco, Genetic Susceptibility, and Accelerated Biological Aging in Adulthood,” Science Advances 18 (2024): eadl3747. 10.1126/sciadv.adl3747 PMC11068008 38701212

[23] W. Huang , Z. Zhang , M. Colucci , et al., “The Mixed Effect of Endocrine‐Disrupting Chemicals on Biological Age Acceleration: Unveiling the Mechanism and Potential Intervention Target,” Environment International 184 (2024): 108447. 38246039 10.1016/j.envint.2024.108447

[24] G. Mauri , G. Patelli , A. Sartore‐Bianchi , et al., “Early‐Onset Cancers: Biological Bases and Clinical Implications,” Cell Reports Medicine 9 (2024): 101737. 10.1016/j.xcrm.2024.101737 PMC11525030 39260369

[25] W. Huang , L. Deng , Q. Wen , et al., “Dynamics of Serum Testosterone and Biological Aging in Men: Insights From Chinese, American, and British Populations,” EClinicalMedicine 82 (2025): 103178. 40235950 10.1016/j.eclinm.2025.103178 PMC11999286

[26] W. Yin , B. Song , C. Yu , et al., “Association of Biological Aging With Prostate Cancer: Insights From the National Health and Nutrition Examination Survey,” Aging Clinical and Experimental Research 36, no. 1 (2024): 209. 39446214 10.1007/s40520-024-02861-0 PMC11502538

[27] K. Ito , K. Mori , R. Kumar , et al., “Global Viewpoints: Evolving Epidemiology and Treatment Patterns of Prostate Cancer in Asia,” BJU International 6 (2025): 970–978. 10.1111/bju.16900 40851349

[28] E. J. Schafer , M. Laversanne , H. Sung , et al., “Recent Patterns and Trends in Global Prostate Cancer Incidence and Mortality: An Update,” European Urology 3 (2025): 302–313. 10.1016/j.eururo.2024.11.013 PMC11862828 39668103

[29] Y. Zhu , M. Mo , Y. Wei , et al., “Epidemiology and Genomics of Prostate Cancer in Asian Men,” Nature Reviews Urology 5 (2021): 282–301. 10.1038/s41585-021-00442-8 33692499

[30] A. Jassim , E. P. Rahrmann , B. D. Simons , et al., “Cancers Make Their Own Luck: Theories of Cancer Origins,” Nature Reviews Cancer 10 (2023): 710–724. 10.1038/s41568-023-00602-5 37488363

[31] K. Hemminki , “Familial Risk and Familial Survival in Prostate Cancer,” World Journal of Urology 2 (2012): 143–148. 10.1007/s00345-011-0801-1 22116601

[32] L. A. Mucci , J. B. Hjelmborg , J. R. Harris , et al., “Familial Risk and Heritability of Cancer Among Twins in Nordic Countries,” Jama 1 (2016): 68–76. 10.1001/jama.2015.17703 PMC5498110 26746459

[33] L. E. Johns and R. S. Houlston , “A Systematic Review and Meta‐Analysis of Familial Prostate Cancer Risk,” BJU International 9 (2003): 789–794. 10.1046/j.1464-410x.2003.04232.x 12780833

[34] N. Mottet , P. Cornford , R. C. N. van den Bergh , et al., EAU‐EANM‐ESTRO‐ESUR‐ISUP‐SIOG guidelines on prostate cancer (2023), https://uroweb.org/guidelines/prostate‐cancer.

[35] N. S. Abul‐Husn , E. R. Soper , J. A. Odgis , et al., “Exome Sequencing Reveals a High Prevalence of BRCA1 and BRCA2 Founder Variants in a Diverse Population‐Based Biobank,” Genome Medicine 12, no. 1 (2019): 2. 31892343 10.1186/s13073-019-0691-1 PMC6938627

[36] P. Nicolosi , E. Ledet , S. Yang , et al., “Prevalence of Germline Variants in Prostate Cancer and Implications for Current Genetic Testing Guidelines,” JAMA Oncology 4 (2019): 523–528. 10.1001/jamaoncol.2018.6760 PMC6459112 30730552

[37] P. Leon , G. Cancel‐Tassin , V. Bourdon , et al., “Bayesian Predictive Model to Assess BRCA2 Mutational Status According to Clinical History: Early Onset, Metastatic Phenotype or Family History of Breast/Ovary Cancer,” The Prostate 6 (2021): 318–325. 10.1002/pros.24109 33599307

[38] S. Maia , M. Cardoso , P. Paulo , et al., “The Role of Germline Mutations in the BRCA1/2 and Mismatch Repair Genes in Men Ascertained for Early‐Onset and/or Familial Prostate Cancer,” Familial Cancer 1 (2016): 111–121. 10.1007/s10689-015-9832-x 26289772

[39] P. Paulo , M. Cardoso , A. Brandão , et al., “Genetic Landscape of Homologous Recombination Repair Genes in Early‐Onset/Familial Prostate Cancer Patients,” Genes, Chromosomes & Cancer 12 (2023): 710–720. 10.1002/gcc.23190 37436117

[40] K. Coelho , J. A. Squire , K. G. Duarte , et al., “Germline Variants in Early and Late‐Onset Brazilian Prostate Cancer Patients,” Urologic Oncology 3 (2024): 68.e11–68.e19. 10.1016/j.urolonc.2024.01.015 38311546

[41] M. Tischkowitz , N. Sabbaghian , A. M. Ray , et al., “Analysis of the Gene Coding for the BRCA2‐Interacting Protein PALB2 in Hereditary Prostate Cancer,” The Prostate 6 (2008): 675–678. 10.1002/pros.20729 PMC2683627 18288683

[42] T. Nyberg , D. Frost , D. Barrowdale , et al., “Prostate Cancer Risks for Male BRCA1 and BRCA2 Mutation Carriers: A Prospective Cohort Study,” European Urology 1 (2020): 24–35. 10.1016/j.eururo.2019.08.025 PMC6926480 31495749

[43] E. K. Bancroft , E. C. Page , M. N. Brook , et al., “A Prospective Prostate Cancer Screening Programme for Men With Pathogenic Variants in Mismatch Repair Genes (IMPACT): Initial Results From an International Prospective Study,” The Lancet Oncology 11 (2021): 1618–1631. 10.1016/S1470-2045(21)00522-2 PMC8576477 34678156

[44] E. K. Bancroft , E. C. Page , E. Castro , et al., “Targeted Prostate Cancer Screening in BRCA1 and BRCA2 Mutation Carriers: Results From the Initial Screening Round of the IMPACT Study,” European Urology 3 (2014): 489–499. 10.1016/j.eururo.2014.01.003 PMC4105321 24484606

[45] E. C. Page , E. K. Bancroft , M. N. Brook , et al., “Interim Results From the IMPACT Study: Evidence for Prostate‐Specific Antigen Screening in BRCA2 Mutation Carriers,” European Urology 6 (2019): 831–842. 10.1016/j.eururo.2019.08.019 PMC6880781 31537406

[46] N. Segal , Y. Ber , O. Benjaminov , et al., “Imaging‐Based Prostate Cancer Screening Among BRCA Mutation Carriers‐Results From the First Round of Screening,” Annals of Oncology: Official Journal of the European Society for Medical Oncology 11 (2020): 1545–1552. 10.1016/j.annonc.2020.06.025 32958357

[47] C. M. Ewing , A. M. Ray , E. M. Lange , et al., “Germline Mutations in HOXB13 and Prostate‐Cancer Risk,” The New England Journal of Medicine 2 (2012): 141–149. 10.1056/NEJMoa1110000 PMC3779870 22236224

[48] R. Karlsson , M. Aly , M. Clements , et al., “A Population‐Based Assessment of Germline HOXB13 G84E Mutation and Prostate Cancer Risk,” European Urology 1 (2014): 169–176. 10.1016/j.eururo.2012.07.027 22841674

[49] V. H. Laitinen , T. Wahlfors , L. Saaristo , et al., “HOXB13 G84E Mutation in Finland: Population‐Based Analysis of Prostate, Breast, and Colorectal Cancer Risk,” Cancer Epidemiology, Biomarkers & Prevention: A Publication of the American Association for Cancer Research, Cosponsored by the American Society of Preventive Oncology 3 (2013): 452–460. 10.1158/1055-9965.EPI-12-1000-T 23292082

[50] J. S. Witte , J. Mefford , S. J. Plummer , et al., “HOXB13 Mutation and Prostate Cancer: Studies of Siblings and Aggressive Disease,” Cancer Epidemiology, Biomarkers & Prevention: A Publication of the American Association for Cancer Research, Cosponsored by the American Society of Preventive Oncology 4 (2013): 675–680. 10.1158/1055-9965.EPI-12-1154 PMC3617049 23396964

[51] J. P. Breyer , T. G. Avritt , K. M. McReynolds , et al., “Confirmation of the HOXB13 G84E Germline Mutation in Familial Prostate Cancer,” Cancer Epidemiology, Biomarkers & Prevention: A Publication of the American Association for Cancer Research, Cosponsored by the American Society of Preventive Oncology 8 (2012): 1348–1353. 10.1158/1055-9965.EPI-12-0495 PMC3415588 22714738

[52] M. R. Akbari , J. Trachtenberg , J. Lee , et al., “Association Between Germline HOXB13 G84E Mutation and Risk of Prostate Cancer,” Journal of the National Cancer Institute 16 (2012): 1260–1262. 10.1093/jnci/djs288 22781434

[53] F. R. Schroeck , K. A. Zuhlke , J. Siddiqui , et al., “Testing for the Recurrent HOXB13 G84E Germline Mutation in Men With Clinical Indications for Prostate Biopsy,” The Journal of Urology 3 (2013): 849–853. 10.1016/j.juro.2012.09.117 PMC4193792 23036981

[54] R. Na , J. Wei , C. J. Sample , et al., “The HOXB13 Variant X285K Is Associated With Clinical Significance and Early Age at Diagnosis in African American Prostate Cancer Patients,” British Journal of Cancer 5 (2022): 791–796. 10.1038/s41416-021-01622-4 PMC8888559 34799695

[55] M. Cardoso , S. Maia , A. Brandão , et al., “Exome Sequencing of Affected Duos and Trios Uncovers PRUNE2 as a Novel Prostate Cancer Predisposition Gene,” British Journal of Cancer 6 (2023): 1077–1085. 10.1038/s41416-022-02125-6 PMC10006409 36564567

[56] E. M. Lange , J. V. Ribado , K. A. Zuhlke , et al., “Assessing the Cumulative Contribution of New and Established Common Genetic Risk Factors to Early‐Onset Prostate Cancer,” Cancer Epidemiology, Biomarkers & Prevention: A Publication of the American Association for Cancer Research, Cosponsored by the American Society of Preventive Oncology 5 (2016): 766–772. 10.1158/1055-9965.EPI-14-0995 PMC4873425 26671023

[57] E. M. Lange , A. M. Johnson , Y. Wang , et al., “Genome‐Wide Association Scan for Variants Associated With Early‐Onset Prostate Cancer,” PloS One 9, no. 4 (2014): e93436. 24740154 10.1371/journal.pone.0093436 PMC3989171

[58] T. A. Desai , Å. K. Hedman , M. Dimitriou , et al., “Identifying Proteomic Risk Factors for Overall, Aggressive, and Early Onset Prostate Cancer Using Mendelian Randomisation and Tumour Spatial Transcriptomics,” EBioMedicine 105 (2024): 105168. 38878676 10.1016/j.ebiom.2024.105168 PMC11233900

[59] F. R. Schumacher , A. A. Al Olama , S. I. Berndt , et al., “Association Analyses of More Than 140,000 Men Identify 63 New Prostate Cancer Susceptibility Loci,” Nature Genetics 7 (2018): 928–936. 10.1038/s41588-018-0142-8 PMC6568012 29892016

[60] R. J. Klein , E. Vertosick , D. Sjoberg , et al., “Prostate Cancer Polygenic Risk Score and Prediction of Lethal Prostate Cancer,” NPJ Precision Oncology 6, no. 1 (2022): 25. 35396534 10.1038/s41698-022-00266-8 PMC8993880

[61] S. Benafif , H. Ni Raghallaigh , E. McGrowder , et al., “The BARCODE1 Pilot: A Feasibility Study of Using Germline Single Nucleotide Polymorphisms to Target Prostate Cancer Screening,” BJU International 3 (2022): 325–336. 10.1111/bju.15535 PMC9292247 34214236

[62] J. K. McHugh , E. K. Bancroft , E. Saunders , et al., “Assessment of a Polygenic Risk Score in Screening for Prostate Cancer,” The New England Journal of Medicine 14 (2025): 1406–1417. 10.1056/NEJMoa2407934 PMC7617604 40214032

[63] D. V. Conti , B. F. Darst , L. C. Moss , et al., “Trans‐Ancestry Genome‐Wide Association Meta‐Analysis of Prostate Cancer Identifies New Susceptibility Loci and Informs Genetic Risk Prediction,” Nature Genetics 1 (2021): 65–75. 10.1038/s41588-020-00748-0 PMC8148035 33398198

[64] A. Plym , K. L. Penney , S. Kalia , et al., “Evaluation of a Multiethnic Polygenic Risk Score Model for Prostate Cancer,” Journal of the National Cancer Institute 5 (2022): 771–774. 10.1093/jnci/djab058 PMC9086757 33792693

[65] F. Chen , B. F. Darst , R. K. Madduri , et al., “Validation of a Multi‐Ancestry Polygenic Risk Score and Age‐Specific Risks of Prostate Cancer: A Meta‐Analysis Within Diverse Populations,” eLife 11 (2022): e78304. 35801699 10.7554/eLife.78304 PMC9322982

[66] Y. Cheng , L. Wu , J. Xin , et al., “An Early‐Onset Specific Polygenic Risk Score Optimizes Age‐Based Risk Estimate and Stratification of Prostate Cancer: Population‐Based Cohort Study,” Journal of Translational Medicine 22 (2024): 366. 38632662 10.1186/s12967-024-05190-y PMC11025178

[67] A. Wang , J. Shen , A. A. Rodriguez , et al., “Characterizing Prostate Cancer Risk Through Multi‐Ancestry Genome‐Wide Discovery of 187 Novel Risk Variants,” Nature Genetics 12 (2023): 2065–2074. 10.1038/s41588-023-01534-4 PMC10841479 37945903

[68] A. Plym , Y. Zhang , K. H. Stopsack , et al., “Early Prostate Cancer Deaths Among Men With Higher vs Lower Genetic Risk,” JAMA Network Open 7, no. 7 (2024): e2420034. 38958976 10.1001/jamanetworkopen.2024.20034 PMC11222990

[69] O. Bergengren , K. R. Pekala , K. Matsoukas , et al., “2022 Update on Prostate Cancer Epidemiology and Risk Factors‐A Systematic Review,” European Urology 2 (2023): 191–206. 10.1016/j.eururo.2023.04.021 PMC10851915 37202314

[70] M. B. Clements , E. A. Vertosick , L. Guerrios‐Rivera , et al., “Defining the Impact of Family History on Detection of High‐Grade Prostate Cancer in a Large Multi‐Institutional Cohort,” European Urology 2 (2022): 163–169. 10.1016/j.eururo.2021.12.011 PMC9243191 34980493

[71] M. Kiciński , J. Vangronsveld , and T. S. Nawrot , “An Epidemiological Reappraisal of the Familial Aggregation of Prostate Cancer: A Meta‐Analysis,” PLoS ONE 10 (2011): e27130. 10.1371/journal.pone.0027130 PMC3205054 22073129

[72] A. Varma , J. Maharjan , A. Garikipati , et al., “Early Prediction of Prostate Cancer Risk in Younger Men Using Polygenic Risk Scores and Electronic Health Records,” Cancer Medicine 1 (2023): 379–386. 10.1002/cam4.4934 PMC9844630 35751453

[73] Y. Xu , D. Huang , Y. Wu , et al., “Family History Is Significantly Associated With Prostate Cancer and Its Early Onset in Chinese Population,” The Prostate 15 (2019): 1762–1766. 10.1002/pros.23900 31497879

[74] K. S. Lee , K. C. Koo , and B. H. Chung , “The Impact of a Family History of Prostate Cancer on the Prognosis and Features of the Disease in Korea: Results From a Cross‐Sectional Longitudinal Pilot Study,” International Urology and Nephrology 12 (2017): 2119–2125. 10.1007/s11255-017-1696-6 28905176

[75] J. L. Beebe‐Dimmer , A. L. Kapron , A. M. Fraser , et al., “Risk of Prostate Cancer Associated With Familial and Hereditary Cancer Syndromes,” Journal of Clinical Oncology: Official Journal of the American Society of Clinical Oncology 16 (2020): 1807–1813. 10.1200/JCO.19.02808 PMC7255976 32208047

[76] E. Kharazmi , M. Fallah , K. Sundquist , et al., “Familial Risk of Early and Late Onset Cancer: Nationwide Prospective Cohort Study,” Bmj (Clinical Research edition) 345 (2012): e8076. 10.1136/bmj.e8076 PMC3527651 23257063

[77] S. Rohrmann , W. W. Roberts , P. C. Walsh , et al., “Family History of Prostate Cancer and Obesity in Relation to High‐Grade Disease and Extraprostatic Extension in Young Men With Prostate Cancer,” The Prostate 2 (2003): 140–146. 10.1002/pros.10211 12661039

[78] E. C. Dee , R. Todd , K. Ng , et al., “Racial Disparities in Prostate Cancer in the UK and the USA: Similarities, Differences and Steps Forwards,” Nature Reviews Urology 4 (2025): 223–234. 10.1038/s41585-024-00948-x 39424981

[79] T. R. Rebbeck , “Prostate Cancer Disparities by Race and Ethnicity: From Nucleotide to Neighborhood,” Cold Spring Harbor Perspectives in Medicine 8, no. 9 (2018): a030387. 29229666 10.1101/cshperspect.a030387 PMC6120694

[80] W. G. Nelson , O. W. Brawley , W. B. Isaacs , et al., “Health Inequity Drives Disease Biology to Create Disparities in Prostate Cancer Outcomes,” The Journal of Clinical Investigation 3 (2022): e155031. 10.1172/JCI155031 PMC8803327 35104804

[81] E. N. Butler , S. P. Kelly , V. H. Coupland , et al., “Fatal Prostate Cancer Incidence Trends in the United States and England by Race, Stage, and Treatment,” British Journal of Cancer 3 (2020): 487–494. 10.1038/s41416-020-0859-x PMC7403310 32433602

[82] T. R. Rebbeck and G. P. Haas , “Temporal Trends and Racial Disparities in Global Prostate Cancer Prevalence,” The Canadian Journal of Urology 5 (2014): 7496–7506. PMC4955669 25347377

[83] I. J. Powell , C. H. Bock , J. J. Ruterbusch , et al., “Evidence Supports a Faster Growth Rate and/or Earlier Transformation to Clinically Significant Prostate Cancer in Black Than in White American Men, and Influences Racial Progression and Mortality Disparity,” The Journal of Urology 5 (2010): 1792–1796. 10.1016/j.juro.2010.01.015 PMC3840791 20299055

[84] Y. Ben‐Shlomo , S. Evans , F. Ibrahim , et al., “The Risk of Prostate Cancer Amongst Black Men in the United Kingdom: The PROCESS Cohort Study,” European Urology 1 (2008): 99–105. 10.1016/j.eururo.2007.02.047 17368710

[85] S. Smani , M. Novosel , R. Sutherland , et al., “Association Between Sociodemographic Factors and Diagnosis of Lethal Prostate Cancer in Early Life,” Urologic Oncology 2 (2024): 28.e9–28.e20. 10.1016/j.urolonc.2023.10.005 38161105

[86] S. Ogino and T. Ugai , “The Global Epidemic of Early‐Onset Cancer: Nature, Nurture, or Both?,” Annals of Oncology: Official Journal of the European Society for Medical Oncology 12 (2024): 1071–1073. 10.1016/j.annonc.2024.08.2336 PMC11624085 39293513

[87] S. Harrison , K. Tilling , E. L. Turner , et al., “Systematic Review and Meta‐Analysis of the Associations Between Body Mass Index, Prostate Cancer, Advanced Prostate Cancer, and Prostate‐Specific Antigen,” Cancer Causes & Control: CCC 5 (2020): 431–449. 10.1007/s10552-020-01291-3 PMC7105428 32162172

[88] A. Perez‐Cornago , K. Smith‐Byrne , E. Hazelwood , et al., “Genetic Predisposition to Metabolically Unfavourable Adiposity and Prostate Cancer Risk: A Mendelian Randomization Analysis,” Cancer Medicine 15 (2023): 16482–16489. 10.1002/cam4.6220 PMC10469819 37305903

[89] H. Cui , W. Zhang , L. Zhang , et al., “Risk Factors for Prostate Cancer: An Umbrella Review of Prospective Observational Studies and Mendelian Randomization Analyses,” PLoS Medicine 21, no. 3 (2024): e1004362. 38489391 10.1371/journal.pmed.1004362 PMC10980219

[90] G. Lee , K. Han , and S. S. Lee , “Different Effect of Obesity and Metabolic Syndrome on Prostate Cancer by Age Group,” American Journal of Cancer Research 12, no. 7 (2022): 3198–3207. 35968325 PMC9360215

[91] S. P. Kelly , B. I. Graubard , G. Andreotti , et al., “Prediagnostic Body Mass Index Trajectories in Relation to Prostate Cancer Incidence and Mortality in the PLCO Cancer Screening Trial,” Journal of the National Cancer Institute 109, no. 3 (2016): djw225. 27754927 10.1093/jnci/djw225 PMC5074530

[92] D. C. Muller , G. G. Giles , J. T. Manning , et al., “Second to Fourth Digit Ratio (2D:4D) and Prostate Cancer Risk in the Melbourne Collaborative Cohort Study,” British Journal of Cancer 3 (2011): 438–440. 10.1038/bjc.2011.253 PMC3172910 21730975

[93] E. L. Watts , A. Perez Cornago , G. K. Fensom , et al., “Circulating Free Testosterone and Risk of Aggressive Prostate Cancer: Prospective and Mendelian Randomisation Analyses in International Consortia,” International Journal of Cancer 7 (2022): 1033–1046. 10.1002/ijc.34116 PMC7613289 35579976

[94] M. H. Ahonen , L. Tenkanen , L. Teppo , et al., “Prostate Cancer Risk and Prediagnostic Serum 25‐Hydroxyvitamin D Levels (Finland),” Cancer Causes & Control: CCC 9 (2000): 847–852. 10.1023/a:1008923802001 11075874

[95] E. L. Watts , A. Perez‐Cornago , G. K. Fensom , et al., “Circulating Insulin‐Like Growth Factors and Risks of Overall, Aggressive and Early‐Onset Prostate Cancer: A Collaborative Analysis of 20 Prospective Studies and Mendelian Randomization Analysis,” International Journal of Epidemiology 1 (2023): 71–86. 10.1093/ije/dyac124 PMC9908067 35726641

[96] J. Zheng , J. Lu , J. Qi , et al., “The Effect of SGLT2 Inhibition on Prostate Cancer: Mendelian Randomization and Observational Analysis Using Electronic Healthcare and Cohort Data,” Cell Reports Medicine 8 (2024): 101688. 10.1016/j.xcrm.2024.101688 PMC11384955 39168098

[97] A. Ioannidou , E. L. Watts , A. Perez‐Cornago , et al., “The Relationship Between Lipoprotein A and Other Lipids With Prostate Cancer Risk: A Multivariable Mendelian Randomisation Study,” PLoS Medicine 19, no. 1 (2022): e1003859. 35085228 10.1371/journal.pmed.1003859 PMC8794090

[98] S. Fang , J. Yarmolinsky , D. Gill , et al., “Association Between Genetically Proxied PCSK9 Inhibition and Prostate Cancer Risk: A Mendelian Randomisation Study,” PLoS Medicine 20, no. 1 (2023): e1003988. 36595504 10.1371/journal.pmed.1003988 PMC9810198

[99] R. Liu , S. Wu , B. Zhang , et al., “The Association Between Sleep Duration and Prostate Cancer: A Systematic Review and Meta‐Analysis,” Medicine 28 (2020): e21180. 10.1097/MD.0000000000021180 PMC7360243 32664160

[100] C. W and C. Lin , “Sleep Disorders Associated With Risk of Prostate Cancer: A Population‐Based Cohort Study,” BMC Cancer 19 (2019): 146. 30760242 10.1186/s12885-019-5361-6 PMC6375129

[101] S. Behboudi‐Gandevani , R. Bidhendi‐Yarandi , M. H. Panahi , et al., “A Systematic Review and Meta‐Analysis of Male Infertility and the Subsequent Risk of Cancer,” Frontiers in Oncology 11 (2021): 696702. 34722244 10.3389/fonc.2021.696702 PMC8551623

[102] Y. Al‐Jebari , A. Elenkov , E. Wirestrand , et al., “Risk of Prostate Cancer for Men Fathering Through Assisted Reproduction: Nationwide Population Based Register Study,” BMJ (Clinical Research edition) 366 (2019): l5214. 10.1136/bmj.l5214 PMC6759809 31554611

[103] A. Dutta , H. Uno , A. Holman , et al., “Racial Differences in Prostate Cancer Risk in Young HIV‐Positive and HIV‐Negative Men: A Prospective Cohort Study,” Cancer Causes & Control: CCC 7 (2017): 767–777. 10.1007/s10552-017-0896-9 PMC5557016 28451806

[104] S. Sutcliffe , E. Giovannucci , A. M. De Marzo , et al., “Gonorrhea, Syphilis, Clinical Prostatitis, and the Risk of Prostate Cancer,” Cancer Epidemiology, Biomarkers & Prevention: A Publication of the American Association for Cancer Research, Cosponsored by the American Society of Preventive Oncology 11 (2006): 2160–2166. 10.1158/1055-9965.EPI-05-0913 17119041

[105] M. G. Kimlin , D. R. Youlden , A. M. Brodie , et al., “Risk of Second Primary Cancer in Survivors of in Situ Melanoma,” The Journal of Investigative Dermatology 4 (2019): 842–847. 10.1016/j.jid.2018.11.001 30423330

[106] D. Huo , J. T. Hetzel , H. Roy , et al., “Association of Colorectal Cancer and Prostate Cancer and Impact of Radiation Therapy,” Cancer Epidemiology, Biomarkers & Prevention: A Publication of the American Association for Cancer Research, Cosponsored by the American Society of Preventive Oncology 7 (2009): 1979–1985. 10.1158/1055-9965.EPI-09-0241 19531678

[107] E. Pukkala , J. I. Martinsen , E. Weiderpass , et al., “Cancer Incidence Among Firefighters: 45 Years of Follow‐Up in Five Nordic Countries,” Occupational and Environmental Medicine 6 (2014): 398–404. 10.1136/oemed-2013-101803 24510539

[108] R. D. Daniels , T. L. Kubale , J. H. Yiin , et al., “Mortality and Cancer Incidence in a Pooled Cohort of US Firefighters From San Francisco, Chicago and Philadelphia (1950‐2009),” Occupational and Environmental Medicine 6 (2014): 388–397. 10.1136/oemed-2013-101662 PMC4499779 24142974

[109] K. H. Barry , J. I. Martinsen , M. C. R. Alavanja , et al., “Risk of Early‐Onset Prostate Cancer Associated With Occupation in the Nordic Countries,” European Journal of Cancer (Oxford, England: 1990) 87 (2017): 92–100. 29132062 10.1016/j.ejca.2017.09.023 PMC6312186

[110] X. Xiong , S. Zhang , X. Liao , et al., “An Umbrella Review of the Evidence Associating Occupational Carcinogens and Cancer Risk at 19 Anatomical Sites,” Environmental Pollution (Barking, Essex: 1987) 345 (2024): 123531. 38341059 10.1016/j.envpol.2024.123531

[111] J. Weischenfeldt , R. Simon , L. Feuerbach , et al., “Integrative Genomic Analyses Reveal an Androgen‐Driven Somatic Alteration Landscape in Early‐Onset Prostate Cancer,” Cancer Cell 2 (2013): 159–170. 10.1016/j.ccr.2013.01.002 23410972

[112] M. F. Berger , M. S. Lawrence , F. Demichelis , et al., “The Genomic Complexity of Primary Human Prostate Cancer,” Nature 7333 (2011): 214–220. 10.1038/nature09744 PMC3075885 21307934

[113] B. S. Carver , J. Tran , Z. Chen , et al., “ETS Rearrangements and Prostate Cancer Initiation,” Nature 7231 (2009): E1, discussion E2‐3. 10.1038/nature07738 PMC2967456 19212347

[114] S. A. Tomlins , D. R. Rhodes , S. Perner , et al., “Recurrent Fusion of TMPRSS2 and ETS Transcription Factor Genes in Prostate Cancer,” Science (New York, New York) 5748 (2005): 644–648. 10.1126/science.1117679 16254181

[115] S. A. Tomlins , B. Laxman , S. Varambally , et al., “Role of the TMPRSS2‐ERG Gene Fusion in Prostate Cancer,” Neoplasia (New York, New York) 2 (2008): 177–188. 10.1593/neo.07822 PMC2244693 18283340

[116] C. Magi‐Galluzzi , T. Tsusuki , P. Elson , et al., “TMPRSS2‐ERG Gene Fusion Prevalence and Class Are Significantly Different in Prostate Cancer of Caucasian, African‐American and Japanese Patients,” The Prostate 5 (2011): 489–497. 10.1002/pros.21265 20878952

[117] J. Blackburn , S. Vecchiarelli , E. E. Heyer , et al., “TMPRSS2‐ERG Fusions Linked to Prostate Cancer Racial Health Disparities: A Focus on Africa,” The Prostate 10 (2019): 1191–1196. 10.1002/pros.23823 PMC6617820 31090091

[118] C. E. Barbieri , S. C. Baca , M. S. Lawrence , et al., “Exome Sequencing Identifies Recurrent SPOP, FOXA1 and MED12 Mutations in Prostate Cancer,” Nature Genetics 44, no. 6 (2012): 685–689. 22610119 10.1038/ng.2279 PMC3673022

[119] G. Schaefer , J. Mosquera , R. Ramoner , et al., “Distinct ERG Rearrangement Prevalence in Prostate Cancer: Higher Frequency in Young Age and in Low PSA Prostate Cancer,” Prostate Cancer and Prostatic Diseases 2 (2013): 132–138. 10.1038/pcan.2013.4 PMC3655380 23381693

[120] Z. Lu , S. R. Williamson , S. Carskadon , et al., “, ETV1, and ETV4 on Whole‐Mount Radical Prostatectomy Tissue,” The Prostate 1 (2020): 38–50. 10.1002/pros.23914 31584209

[121] Z. R. Chalmers , M. C. Burns , E. M. Ebot , et al., “Early‐Onset Metastatic and Clinically Advanced Prostate Cancer Is a Distinct Clinical and Molecular Entity Characterized by Increased TMPRSS2‐ERG Fusions,” Prostate Cancer and Prostatic Diseases 2 (2021): 558–566. 10.1038/s41391-020-00314-z PMC8134051 33420417

[122] S. Steurer , P. S. Mayer , M. Adam , et al., “TMPRSS2‐ERG Fusions Are Strongly Linked to Young Patient Age in Low‐Grade Prostate Cancer,” European Urology 6 (2014): 978–981. 10.1016/j.eururo.2014.06.027 25015038

[123] P. C. Boutros , M. Fraser , N. J. Harding , et al., “Spatial Genomic Heterogeneity Within Localized, Multifocal Prostate Cancer,” Nature Genetics 7 (2015): 736–745. 10.1038/ng.3315 26005866

[124] Z. Zhou , A. Flesken‐Nikitin , D. C. Corney , et al., “Synergy of p53 and Rb Deficiency in a Conditional Mouse Model for Metastatic Prostate Cancer,” Cancer Research 16 (2006): 7889–7898. 10.1158/0008-5472.CAN-06-0486 16912162

[125] J. Sivalingam , K. H. Cho , Y. Shi , et al., “A MYC Family Switch: L‐MYC Drives and Maintains Neuroendocrine Lineage Programs in Prostate Cancer,” bioRxiv: The Preprint Server for Biology 2025 (2025): 696507. 10.1016/j.neo.2026.101307 PMC13098336 42001796

[126] S. R. Williamson , S. Zhang , J. L. Yao , et al., “ERG‐TMPRSS2 Rearrangement Is Shared by Concurrent Prostatic Adenocarcinoma and Prostatic Small Cell Carcinoma and Absent in Small Cell Carcinoma of the Urinary Bladder: Evidence Supporting Monoclonal Origin,” Modern Pathology: An Official Journal of the United States and Canadian Academy of Pathology, Inc 8 (2011): 1120–1127. 10.1038/modpathol.2011.56 PMC3441178 21499238

[127] G. Mauri , G. Patelli , G. Crisafulli , et al., “Tumor “Age” in Early‐Onset Colorectal Cancer,” Cell 3 (2025): 589–593. 10.1016/j.cell.2024.12.003 39919707

[128] K. Bhat , F. F. Onol , M. C. Moschovas , et al., “Robotic‐Assisted Radical Prostatectomy in Young Adults: Age‐Stratified Oncological and Functional Outcomes,” Journal of Robotic Surgery 5 (2022): 1057–1066. 10.1007/s11701-021-01334-0 34813023

[129] C. G. Rogers , L. M. Su , R. E. Link , et al., “Age Stratified Functional Outcomes After Laparoscopic Radical Prostatectomy,” The Journal of Urology 1 (2006): 2448–2452. 10.1016/j.juro.2006.07.153 17085126

[130] D. B. Samadi , D. Sebrow , A. R. Hobbs , et al., “Clinicopathological, Functional, and Immediate Oncologic Outcome Assessment in Men Aged≤50 Years With Prostate Cancer After Robotic Prostatectomy,” Urologic Oncology 1 (2017): 30.e17–30.e24. 10.1016/j.urolonc.2016.07.016 27567690

[131] M. H. Soltani , R. Khabazian , M. Dadpour , et al., “Comparison of Histopathological Features, Survival, and Oncological Outcome Between Patients Under and Above 55‐Year‐Old With Prostate Cancer Who Underwent Radical Prostatectomy,” Urologia 1 (2023): 83–88. 10.1177/03915603221078266 35191332

[132] M. S. Chung , M. Shim , J. S. Cho , et al., “Pathological Characteristics of Prostate Cancer in Men Aged < 50 Years Treated With Radical Prostatectomy: A Multi‐Centre Study in Korea,” Journal of Korean Medical Science 34, no. 10 (2019): e78. 30886549 10.3346/jkms.2019.34.e78 PMC6417998

[133] B. Song , H. Lee , M. S. Lee , et al., “Outcomes of Men Aged ≤50 Years Treated With Radical Prostatectomy: A Retrospective Analysis,” Asian Journal of Andrology 2 (2019): 150–155. 10.4103/aja.aja_92_18 PMC6413547 30460935

[134] T. H. Huang , J. Y. Kuo , Y. H. Huang , et al., “Prostate Cancer in Young Adults‐Seventeen‐Year Clinical Experience of a Single Center,” Journal of the Chinese Medical Association: JCMA 1 (2017): 39–43. 10.1016/j.jcma.2016.10.004 27914715

[135] N. D. Dantanarayana , T. Hossack , P. Cozzi , et al., “Men Under the Age of 55 Years With Screen Detected Prostate Cancer Do Not Have Less Significant Disease Compared to Older Men in a Population of Patients in Australia,” BMC Urology 15 (2015): 124. 26715039 10.1186/s12894-015-0117-3 PMC4696233

[136] J. Macneil , F. Maclean , W. Delprado , et al., “Pathological Characteristics of Prostate Cancer Occurring in Younger Men: A Retrospective Study of Prostatectomy Patients,” Urology 134 (2019): 163–167. 31541648 10.1016/j.urology.2019.08.048

[137] Y. C. Ding , H. Wu , E. Davicioni , et al., “Prostate Cancer in Young Men Represents a Distinct Clinical Phenotype: Gene Expression Signature to Predict Early Metastases,” Journal of Translational Genetics and Genomics 5 (2021): 50–61. 33928239 10.20517/jtgg.2021.01 PMC8081383

[138] Y. Hu , Q. Qi , Y. Zheng , et al., “Nomogram for Predicting the Overall Survival of Patients With Early‐Onset Prostate Cancer: A Population‐Based Retrospective Study,” Cancer Medicine 17 (2022): 3260–3271. 10.1002/cam4.4694 PMC9468440 35322943

[139] C. V. Smith , J. J. Bauer , R. R. Connelly , et al., “Prostate Cancer in Men Age 50 Years or Younger: A Review of the Department of Defense Center for Prostate Disease Research Multicenter Prostate Cancer Database,” The Journal of Urology 6 (2000): 1964–1967. 10.1016/s0022-5347(05)66929-7 11061892

[140] M. A. Khan , M. Han , A. W. Partin , et al., “Long‐Term Cancer Control of Radical Prostatectomy in Men Younger Than 50 Years of Age: Update 2003,” Urology 62 (2003): 86–91. 12837428 10.1016/s0090-4295(03)00404-7

[141] S. K. Bechis , P. R. Carroll , and M. R. Cooperberg , “Impact of Age at Diagnosis on Prostate Cancer Treatment and Survival,” Journal of Clinical Oncology: Official Journal of the American Society of Clinical Oncology 2 (2011): 235–241. 10.1200/JCO.2010.30.2075 PMC3058279 21135285

[142] A. Becker , P. Tennstedt , J. Hansen , et al., “Functional and Oncological Outcomes of Patients Aged <50 Years Treated With Radical Prostatectomy for Localised Prostate Cancer in a European Population,” BJU International 1 (2014): 38–45. 10.1111/bju.12407 24053677

[143] M. Rouprêt , G. Fromont , M. O. Bitker , et al., “Outcome After Radical Prostatectomy in Young Men With or Without a Family History of Prostate Cancer,” Urology 5 (2006): 1028–1032. 10.1016/j.urology.2005.11.035 16698363

[144] A. Thorstenson , H. Garmo , J. Adolfsson , et al., “Cancer Specific Mortality in Men Diagnosed With Prostate Cancer Before Age 50 Years: A Nationwide Population Based Study,” The Journal of Urology 1 (2017): 61–66. 10.1016/j.juro.2016.06.080 27328367

[145] S. Zeighami , A. Ariafar , A. Makarem , et al., “Survival and Oncological Outcomes for Young Men (≤55 Years) Undergoing Radical Prostatectomy for Localized Prostate Cancer,” Archivio Italiano di Urologia, Andrologia: Organo Ufficiale [di] Societa Italiana di Ecografia Urologica e Nefrologica 1 (2025): 12658. 10.4081/aiua.2025.12658 39851058

[146] T. Kimura , M. Onozawa , J. Miyazaki , et al., “Prognostic Impact of Young Age on Stage IV Prostate Cancer Treated With Primary Androgen Deprivation Therapy,” International Journal of Urology: Official Journal of the Japanese Urological Association 6 (2014): 578–583. 10.1111/iju.12389 24405474

[147] C. Twiss , D. Slova , and H. Lepor , “Outcomes for Men Younger Than 50 Years Undergoing Radical Prostatectomy,” Urology 1 (2005): 141–146. 10.1016/j.urology.2005.01.049 15992906

[148] N. Baniak , L. M. Sholl , D. A. Mata , et al., “Clinicopathological and Molecular Characteristics of Prostate Cancer Diagnosed in Young Men Aged up to 45 Years,” Histopathology 6 (2021): 857–870. 10.1111/his.14315 33306242

[149] A. Magheli , S. Rais‐Bahrami , E. B. Humphreys , et al., “Impact of Patient Age on Biochemical Recurrence Rates Following Radical Prostatectomy,” The Journal of Urology 5 (2007): 1933–1937, discussion 1937–8. 10.1016/j.juro.2007.07.016 17868723

[150] S. J. Freedland , J. J. Presti , C. J. Kane , et al., “Do Younger Men Have Better Biochemical Outcomes After Radical Prostatectomy?,” Urology 3 (2004): 518–522. 10.1016/j.urology.2003.10.045 15028449

[151] G. Mehring , D. Tilki , H. Heinzer , et al., “Histopathological Results of Radical Prostatectomy Specimen of Men Younger Than 50 Years of Age at the Time of Surgery: Possible Implications for Prostate Cancer Screening Programs?,” World Journal of Urology 2 (2023): 421–425. 10.1007/s00345-023-04287-1 PMC9947052 36656332

[152] D. Tilki , V. Maurer , R. S. Pompe , et al., “Tumor Characteristics, Oncological and Functional Outcomes After Radical Prostatectomy in Very Young Men ≤ 45 Years of Age,” World Journal of Urology 1 (2020): 95–101. 10.1007/s00345-019-02740-8 30937571

[153] R. T. Coman , N. Crisan , I. Andras , et al., “Outcomes of Robotic‐Assisted Radical Prostatectomy for Patients in Two Extreme Age‐Groups (< 50 Years vs >65 Years),” Clujul Medical (1957) 91, no. 1 (2018): 92–97. 29440957 10.15386/cjmed-825 PMC5808275

[154] Y. Lu , H. H. Huang , W. Lau , et al., “Survival Outcomes of Asian Younger Men (< 55 Years) Undergoing Radical Prostatectomy: A Review of Prostate Cancer Database in a Tertiary Hospital in Singapore,” International Urology and Nephrology 10 (2020): 1885–1891. 10.1007/s11255-020-02518-7 32476081

[155] A. A. Antunes , A. Crippa , M. F. Dall'Oglio , et al., “Age Impact in Clinicopathologic Presentation and the Clinical Evolution of Prostate Cancer in Patients Submitted to Radical Prostatectomy,” International Braz J Urol: Official Journal of the Brazilian Society of Urology 1 (2006): 48–55. 10.1590/s1677-55382006000100008 16519828

[156] A. Billis , L. A. Magna , M. M. Lira , et al., “Relationship of Age to Outcome and Clinicopathologic Findings in Men Submitted to Radical Prostatectomy,” International Braz J Urol: Official Journal of the Brazilian Society of Urology 6 (2005): 534–539, discussion 539–40. 10.1590/s1677-55382005000600004 16386121

[157] S. X. Lin , Y. Zheng , S. Wu , et al., “Impact of Biopsy Perineural Invasion on Younger Prostate Cancer Patients After Radical Prostatectomy,” Scandinavian Journal of Urology 6 (2020): 475–480. 10.1080/21681805.2020.1817143 32930036

[158] S. A. Siddiqui , S. Sengupta , J. M. Slezak , et al., “Impact of Patient Age at Treatment on Outcome Following Radical Retropubic Prostatectomy for Prostate Cancer,” The Journal of Urology 1 (2006): 952–957. 10.1016/S0022-5347(05)00339-3 16469591

[159] S. Prendeville , M. E. Nesbitt , A. J. Evans , et al., “Variant Histology and Clinicopathological Features of Prostate Cancer in Men Younger Than 50 Years Treated With Radical Prostatectomy,” The Journal of Urology 1 (2017): 79–85. 10.1016/j.juro.2017.01.061 28130102

[160] K. A. Moses , L. Y. Chen , D. D. Sjoberg , et al., “Black and White Men Younger Than 50 Years of Age Demonstrate Similar Outcomes After Radical Prostatectomy,” BMC Urology 14 (2014): 98. 25495177 10.1186/1471-2490-14-98 PMC4269868

[161] R. S. Pompe , A. Smith , M. Bandini , et al., “Tumor Characteristics, Treatments, and Oncological Outcomes of Prostate Cancer in Men Aged ≤50 Years: A Population‐Based Study,” Prostate Cancer and Prostatic Diseases 1 (2018): 71–77. 10.1038/s41391-017-0006-9 29339806

[162] S. V. Kotsis , S. L. Spencer , P. A. Peyser , et al., “Early Onset Prostate Cancer: Predictors of Clinical Grade,” The Journal of Urology 4 (2002): 1659–1663. 11912383

[163] D. Milonas , Z. Venclovas , I. Gudinaviciene , et al., “Long‐Term Oncological Outcomes for Young Men Undergoing Radical Prostatectomy for Localized Prostate Cancer,” BioMed Research International 2017 (2017): 9858923. 28299340 10.1155/2017/9858923 PMC5337309

[164] A. Stabile , P. Dell'Oglio , M. Soligo , et al., “Assessing the Clinical Value of Positive Multiparametric Magnetic Resonance Imaging in Young Men With a Suspicion of Prostate Cancer,” European Urology Oncology 4 (2021): 594–600. 31204312 10.1016/j.euo.2019.05.006

[165] G. Ji , C. Huang , G. Song , et al., “Are the Pathological Characteristics of Prostate Cancer More Aggressive or More Indolent Depending Upon the Patient Age?,” BioMed Research International 2017 (2017): 1438027. 28265568 10.1155/2017/1438027 PMC5318620

[166] Y. Xu , X. Yang , T. Si , et al., “Clinicopathological and Prognostic Factors in 106 Prostate Cancer Patients Aged ≤55 Years: A Single‐Center Study in China,” Medical Science Monitor: International Medical Journal of Experimental and Clinical Research 22 (2016): 3935–3942. 27771734 10.12659/MSM.901040 PMC5081234

[167] L. Tan , L. L. Wang , W. Ranasinghe , et al., “Survival Outcomes of Younger Men (< 55 Years) Undergoing Radical Prostatectomy,” Prostate International 1 (2018): 31–35. 10.1016/j.prnil.2017.07.002 PMC5857187 29556487

[168] N. J. Kinnear , G. Kichenadasse , S. Plagakis , et al., “Prostate Cancer in Men Aged Less Than 50 Years at Diagnosis,” World Journal of Urology 11 (2016): 1533–1539. 10.1007/s00345-016-1824-4 27072535

[169] H. J. Shih , S. C. Fang , L. An , et al., “Early‐Onset Prostate Cancer Is Associated With Increased Risks of Disease Progression and Cancer‐Specific Mortality,” The Prostate 2 (2021): 118–126. 10.1002/pros.24087 33152137

[170] G. N. Collins , R. J. Lee , G. B. McKelvie , et al., “Relationship Between Prostate Specific Antigen, Prostate Volume and Age in the Benign Prostate,” British Journal of Urology 4 (1993): 445–450. 10.1111/j.1464-410x.1993.tb15990.x 7684650

[171] S. J. Berry , D. S. Coffey , P. C. Walsh , et al., “The Development of Human Benign Prostatic Hyperplasia With Age,” The Journal of Urology 3 (1984): 474–479. 10.1016/s0022-5347(17)49698-4 6206240

[172] J. E. Oesterling , S. J. Jacobsen , C. G. Chute , et al., “Serum Prostate‐Specific Antigen in a Community‐Based Population of Healthy Men. Establishment of Age‐Specific Reference Ranges,” Jama 7 (1993): 860–864. 7688054

[173] R. Raychaudhuri , D. W. Lin , and R. B. Montgomery , “Prostate Cancer: A Review,” Jama 16 (2025): 1433–1446. 10.1001/jama.2025.0228 40063046

[174] R. M. Merrill and J. S. Bird , “Effect of Young Age on Prostate Cancer Survival: A Population‐Based Assessment (United States),” Cancer Causes & Control: CCC 5 (2002): 435–443. 10.1023/a:1015764507609 12146848

[175] T. J. Daskivich , K. Fan , T. Koyama , et al., “Effect of Age, Tumor Risk, and Comorbidity on Competing Risks for Survival in a U.S. Population‐Based Cohort of Men With Prostate Cancer,” Annals of Internal Medicine 10 (2013): 709–717. 10.7326/0003-4819-158-10-201305210-00005 PMC3760479 23689764

[176] D. W. Lin , M. Porter , and B. Montgomery , “Treatment and Survival Outcomes in Young Men Diagnosed With Prostate Cancer: A Population‐Based Cohort Study,” Cancer 13 (2009): 2863–2871. 10.1002/cncr.24324 PMC2948666 19466697

[177] W. Sheng , H. Zhang , and Y. Lu , “Survival Outcomes of Locally Advanced Prostate Cancer in Patients Aged < 50 Years After Local Therapy in the Contemporary US Population,” International Urology and Nephrology 8 (2018): 1435–1444. 10.1007/s11255-018-1931-9 29982959

[178] W. Hsiao , K. A. Moses , M. Goodman , et al., “Stage IV Prostate Cancer: Survival Differences in Clinical T4, Nodal and Metastatic Disease,” The Journal of Urology 2 (2010): 512–518. 10.1016/j.juro.2010.04.010 20620410

[179] C. Riveros , M. Al‐Toubat , V. Chalfant , et al., “The Impact of Socioeconomic Status on the Survival of Men With Early‐Onset Prostate Cancer,” American Journal of Clinical and Experimental Urology 11, no. 2 (2023): 146–154. 37168939 PMC10165226

[180] J. Li , Z. Cai , W. Wei , et al., “Establishment of Prognostic Nomograms for Early‐Onset Prostate Cancer Patients: A SEER Database Analysis,” Journal of Investigative Surgery: The Official Journal of the Academy of Surgical Research 7 (2022): 1581–1590. 10.1080/08941939.2022.2062495 35414345

[181] P. Cornford , D. Tilki , and R. C. N. van den Bergh , EAU‐EANM‐ESTRO‐ESUR‐ISUP‐SIOG Guidelines on Prostate Cancer (European Association of Urology, 2025), https://uroweb.org/guidelines/prostate‐cancer.

[182] I. Gielchinsky , J. Chang , T. Cusick , et al., “Prostate Cancer in 432 Men Aged <50 Years in the Prostate‐Specific Antigen Era: A New Outlook,” BJU International Suppl 5 (2018): 35–41. 10.1111/bju.14586 30303599

[183] A. R. Mahal , S. Butler , I. Franco , et al., “Conservative Management of Low‐Risk Prostate Cancer Among Young Versus Older Men in the United States: Trends and Outcomes From a Novel National Database,” Cancer 19 (2019): 3338–3346. 10.1002/cncr.32332 31251398

[184] W. L. Ong , S. M. Evans , M. Evans , et al., “Trends in Conservative Management for Low‐Risk Prostate Cancer in a Population‐Based Cohort of Australian Men Diagnosed Between 2009 and 2016,” European Urology Oncology 2 (2021): 319–322. 10.1016/j.euo.2019.04.006 31411964

[185] B. Jain , K. Yamoah , C. S. Lathan , et al., “Racial Disparities in Treatment Delay Among Younger Men With Prostate Cancer,” Prostate Cancer and Prostatic Diseases 3 (2022): 590–592. 10.1038/s41391-021-00479-1 35190652

[186] M. A. Diven , L. Tshering , X. Ma , et al., “Trends in Active Surveillance for Men With Intermediate‐Risk Prostate Cancer,” JAMA Network Open 7, no. 8 (2024): e2429760. 39172448 10.1001/jamanetworkopen.2024.29760 PMC11342134

[187] S. P. Kim , N. D. Shah , N. J. Meropol , et al., “Recommendations of Active Surveillance for Intermediate‐Risk Prostate Cancer: Results From a National Survey of Radiation Oncologists and Urologists,” European Urology Oncology 2 (2019): 189–195. 31017095 10.1016/j.euo.2018.08.004

[188] M. S. Leapman , J. E. Cowan , H. G. Nguyen , et al., “Active Surveillance in Younger Men With Prostate Cancer,” Journal of Clinical Oncology: Official Journal of the American Society of Clinical Oncology 17 (2017): 1898–1904. 10.1200/JCO.2016.68.0058 PMC5466007 28346806

[189] R. K. Sayyid , W. C. Reed , J. Z. Benton , et al., “Pathologic Upgrading in Favorable Intermediate Risk Active Surveillance Patients: Clinical Heterogeneity and Implications for Active Surveillance Decision,” Urologic Oncology 11 (2021): 782.e7–782.e14. 10.1016/j.urolonc.2021.02.017 33766466

[190] E. Ventimiglia , A. Bill‐Axelson , O. Bratt , et al., “Long‐Term Outcomes Among Men Undergoing Active Surveillance for Prostate Cancer in Sweden,” JAMA Network Open 5, no. 9 (2022): e2231015. 36103180 10.1001/jamanetworkopen.2022.31015 PMC9475386

[191] P. M. Parker , K. R. Rice , J. R. Sterbis , et al., “Prostate Cancer in Men Less Than the Age of 50: A Comparison of Race and Outcomes,” Urology 1 (2011): 110–115. 10.1016/j.urology.2010.12.046 21397300

[192] K. C. Huang , M. Dolph , B. Donnelly , et al., “ERG Expression Is Associated With Increased Risk of Biochemical Relapse Following Radical Prostatectomy in Early Onset Prostate Cancer,” Clinical & Translational Oncology: Official Publication of the Federation of Spanish Oncology Societies and of the National Cancer Institute of Mexico 11 (2014): 973–979. 10.1007/s12094-014-1182-x 24796295

[193] J. L. Wright , D. W. Lin , J. E. Cowan , et al., “Quality of Life in Young Men After Radical Prostatectomy,” Prostate Cancer and Prostatic Diseases 1 (2008): 67–73. 10.1038/sj.pcan.4500980 17519925

[194] A. Dal Pra , E. Lalonde , J. Sykes , et al., “TMPRSS2‐ERG Status Is Not Prognostic Following Prostate Cancer Radiotherapy: Implications for Fusion Status and DSB Repair,” Clinical Cancer Research: An Official Journal of the American Association for Cancer Research 18 (2013): 5202–5209. 10.1158/1078-0432.CCR-13-1049 23918607

[195] J. Fontugne , D. Lee , C. Cantaloni , et al., “Recurrent Prostate Cancer Genomic Alterations Predict Response to Brachytherapy Treatment,” Cancer Epidemiology, Biomarkers & Prevention: A Publication of the American Association for Cancer Research, Cosponsored by the American Society of Preventive Oncology 4 (2014): 594–600. 10.1158/1055-9965.EPI-13-1180 PMC4083705 24515272

[196] R. E. Krasnow , D. Rodríguez , R. T. Nagle , et al., “The Impact of Age at the Time of Radiotherapy for Localized Prostate Cancer on the Development of Second Primary Malignancies,” Urologic Oncology 11 (2018): 500.e11–500.e19. 10.1016/j.urolonc.2018.06.007 30249519

[197] R. E. Graff , A. Pettersson , R. T. Lis , et al., “The TMPRSS2:ERG Fusion and Response to Androgen Deprivation Therapy for Prostate Cancer,” The Prostate 9 (2015): 897–906. 10.1002/pros.22973 PMC4424159 25728532

[198] L. Martínez‐Piñeiro , J. A. Schalken , P. Cabri , et al., “Evaluation of Urinary Prostate Cancer Antigen‐3 (PCA3) and TMPRSS2‐ERG Score Changes When Starting Androgen‐Deprivation Therapy With Triptorelin 6‐Month Formulation in Patients With Locally Advanced and Metastatic Prostate Cancer,” BJU International 4 (2014): 608–616. 10.1111/bju.12542 24806330

[199] M. P. Fernandez‐Perez , E. Perez‐Navarro , T. Alonso‐Gordoa , et al., “A Correlative Biomarker Study and Integrative Prognostic Model in Chemotherapy‐Naïve Metastatic Castration‐Resistant Prostate Cancer Treated With Enzalutamide,” The Prostate 4 (2023): 376–384. 10.1002/pros.24469 PMC10107622 36564933

[200] D. C. Danila , A. Anand , C. C. Sung , et al., “TMPRSS2‐ERG Status in Circulating Tumor Cells as a Predictive Biomarker of Sensitivity in Castration‐Resistant Prostate Cancer Patients Treated With Abiraterone Acetate,” European Urology 5 (2011): 897–904. 10.1016/j.eururo.2011.07.011 PMC3185163 21802835

[201] J. L. Boormans , K. G. Hermans , A. C. J. Z. Made , et al., “Expression of the Androgen‐Regulated Fusion Gene TMPRSS2‐ERG Does Not Predict Response to Endocrine Treatment in Hormone‐Naïve, Node‐Positive Prostate Cancer,” European Urology 5 (2010): 830–835. 10.1016/j.eururo.2009.08.013 19716227

[202] G. Attard , J. S. de Bono , C. J. Logothetis , et al., “Improvements in Radiographic Progression‐Free Survival Stratified by ERG Gene Status in Metastatic Castration‐Resistant Prostate Cancer Patients Treated With Abiraterone Acetate,” Clinical Cancer Research: An Official Journal of the American Association for Cancer Research 7 (2015): 1621–1627. 10.1158/1078-0432.CCR-14-1961 PMC4384987 25593303

[203] C. Williams , A. Inderjeeth , W. Hong , et al., “Treatment Patterns and Outcomes for Younger Patients With Metastatic Castration‐Resistant Prostate Cancer (mCRPC); an Australian Prospective Registry Study,” Clinical Genitourinary Cancer 3 (2025): 102345. 10.1016/j.clgc.2025.102345 40319642

[204] S. Rajpar , T. Ibrahim , A. Carmel , et al., “The Benefit of Combining Docetaxel With Androgen Deprivation Therapy in Localized and Metastatic Hormone‐Sensitive Prostate Cancer is Predicted by ERG Expression: An Analysis of Two GETUG Phase 3 Trials,” European Urology Oncology 2 (2025): 296–305. 10.1016/j.euo.2024.06.015 39034169

[205] Ò. Reig , M. Marín‐Aguilera , G. Carrera , et al., “TMPRSS2‐ERG in Blood and Docetaxel Resistance in Metastatic Castration‐Resistant Prostate Cancer,” European Urology 5 (2016): 709–713. 10.1016/j.eururo.2016.02.034 26948395

[206] M. Marín‐Aguilera , Ò. Reig , M. Milà‐Guasch , et al., “The Influence of Treatment Sequence in the Prognostic Value of TMPRSS2‐ERG as Biomarker of Taxane Resistance in Castration‐Resistant Prostate Cancer,” International Journal of Cancer 7 (2019): 1970–1981. 10.1002/ijc.32238 30807643

[207] T. S. Poulsen , A. N. Lørup , P. Kongsted , et al., “TMPRSS2:ERG Gene Fusion Might Predict Resistance to PARP Inhibitors in Metastatic Castration‐Resistant Prostate Cancer,” AntiCancer Research 10 (2024): 4203–4211. 10.21873/anticanres.17250 39348956

[208] S. Köcher , M. E. Elsesy , A. Moustafa , et al., “Overexpression of the ERG Oncogene in Prostate Cancer Identifies Candidates for PARP Inhibitor‐Based Radiosensitization,” The Journal of Clinical Investigation 136, no. 6 (2026): e194949. 41632544 10.1172/JCI194949 PMC12987654

[209] F. Ma , S. Chen , L. Cecchi , et al., “Androgen Deprivation‐Mediated Activation of AKT Is Enhanced in Prostate Cancer With TMPRSS2:ERG Fusion,” The Journal of Clinical Investigation 135, no. 23 (2025): e192368. 41321318 10.1172/JCI192368 PMC12646650

[210] N. Mao , D. Gao , W. Hu , et al., “Aberrant Expression of ERG Promotes Resistance to Combined PI3K and AR Pathway Inhibition Through Maintenance of AR Target Genes,” Molecular Cancer Therapeutics 9 (2019): 1577–1586. 10.1158/1535-7163.MCT-18-1386 PMC6726496 31296553

[211] P. Dave , S. V. Carlsson , and K. Watts , “Randomized Trials of PSA Screening,” Urologic Oncology 1 (2025): 23–28. 10.1016/j.urolonc.2024.05.014 38926075

[212] G. L. Andriole , E. D. Crawford , R. L. Grubb , et al., “Mortality Results From a Randomized Prostate‐Cancer Screening Trial,” The New England Journal of Medicine 13 (2009): 1310–1319. 10.1056/NEJMoa0810696 PMC2944770 19297565

[213] J. E. Shoag , S. Mittal , and J. C. Hu , “Reevaluating PSA Testing Rates in the PLCO Trial,” The New England Journal of Medicine 18 (2016): 1795–1796. 10.1056/NEJMc1515131 27144870

[214] A. Tsodikov , R. Gulati , E. A. M. Heijnsdijk , et al., “Reconciling the Effects of Screening on Prostate Cancer Mortality in the ERSPC and PLCO Trials,” Annals of Internal Medicine 7 (2017): 449–455. 10.7326/M16-2586 PMC5734053 28869989

[215] F. H. Schro¨der , J. Hugosson , M. J. Roobol , et al., “Screening and Prostate‐Cancer Mortality in a Randomized European Study,” The New England Journal of Medicine 13 (2009): 1320–1328. 10.1056/NEJMoa0810084 19297566

[216] F. H. Schro¨der , J. Hugosson , M. J. Roobol , et al., “Screening and Prostate Cancer Mortality: Results of the European Randomised Study of Screening for Prostate Cancer (ERSPC) at 13 Years of Follow‐Up,” Lancet (London, England) 9959 (2014): 2027–2035. 10.1016/S0140-6736(14)60525-0 PMC4427906 25108889

[217] J. Hugosson , S. Carlsson , G. Aus , et al., “Mortality Results From the Göteborg Randomised Population‐Based Prostate‐Cancer Screening Trial,” The Lancet Oncology 8 (2010): 725–732. 10.1016/S1470-2045(10)70146-7 PMC4089887 20598634

[218] M. Fra°nlund , M. Ma°nsson , R. A. Godtman , et al., “Results From 22 Years of Followup in the Göteborg Randomized Population‐Based Prostate Cancer Screening Trial,” The Journal of Urology 2 (2022): 292–300. 10.1097/JU.0000000000002696 PMC9275849 35422134

[219] R. M. Martin , J. L. Donovan , E. L. Turner , et al., “Effect of a Low‐Intensity PSA‐Based Screening Intervention on Prostate Cancer Mortality: The CAP Randomized Clinical Trial,” Jama 9 (2018): 883–895. 10.1001/jama.2018.0154 PMC5885905 29509864

[220] J. Hugosson , M. J. Roobol , M. Månsson , et al., “A 16‐Yr Follow‐Up of the European Randomized Study of Screening for Prostate Cancer,” European Urology 1 (2019): 43–51. 10.1016/j.eururo.2019.02.009 PMC7513694 30824296

[221] P. F. Pinsky , E. Miller , P. Prorok , et al., “Extended Follow‐Up for Prostate Cancer Incidence and Mortality Among Participants in the Prostate, Lung, Colorectal and Ovarian Randomized Cancer Screening Trial,” BJU International 5 (2019): 854–860. 10.1111/bju.14580 PMC6450783 30288918

[222] P. F. Pinsky , P. C. Prorok , K. Yu , et al., “Extended Mortality Results for Prostate Cancer Screening in the PLCO Trial With Median Follow‐Up of 15 Years,” Cancer 4 (2017): 592–599. 10.1002/cncr.30474 PMC5725951 27911486

[223] F. H. Schröder , J. Hugosson , S. Carlsson , et al., “Screening for Prostate Cancer Decreases the Risk of Developing Metastatic Disease: Findings From the European Randomized Study of Screening for Prostate Cancer (ERSPC),” European Urology 5 (2012): 745–752. 10.1016/j.eururo.2012.05.068 22704366

[224] S. P. Basourakos , R. Gulati , R. A. J. Vince , et al., “Harm‐To‐Benefit of Three Decades of Prostate Cancer Screening in Black Men,” NEJM Evidence 1, no. 6 (2022): 10.1056/evidoa2200031. 10.1056/evidoa2200031 PMC9202998 35721307

[225] H. Van Poppel , T. Albreht , P. Basu , et al., “Serum PSA‐Based Early Detection of Prostate Cancer in Europe and Globally: Past, Present and Future,” Nature Reviews Urology 9 (2022): 562–572. 10.1038/s41585-022-00638-6 35974245

[226] American Cancer Society (ACS) American Cancer Society Recommendations for Prostate Cancer Early Detection, Accessed December 4, 2024 (ACS, 2023), https://www.cancer.org/cancer/types/prostate‐cancer/detection‐diagnosis‐staging/acs‐recommendations.html.

[227] J. T. Wei , D. Barocas , S. Carlsson , et al., “Early Detection of Prostate Cancer: AUA/SUO Guideline Part I: Prostate Cancer Screening,” The Journal of Urology 1 (2023): 46–53. 10.1097/JU.0000000000003491 PMC11060750 37096582

[228] NCCN Clinical Practice Guidelines in Oncology‐Prostate Cancer Early Detection (Version 2.2025), https://www.nccn.org/guidelines/guidelines‐detail?category=2&id=1460. 10.6004/jnccn.2010.0016 20141680

[229] P. Cornford , D. Tilki , R. C. N. van den Bergh , et al. EAU‐EANM‐ESTRO‐ESUR‐ISUP‐SIOG Guidelines on Prostate Cancer. 2026, https://uroweb.org/guidelines/prostate‐cancer.

[230] X. Zhang , J. Wu , J. Huang , et al., “White Paper on Prostate Cancer Screening in Chinese,” Chinese Journal of Urology 46 (2025): E1–E23.

[231] European Association of Urology (EAU) PRAISE‐U. Smart Early Detection of Prostate Cancer, Accessed December 4,2024 (EAU, 2024), https://uroweb.org/praise‐u.

[232] D. C. Grossman , S. J. Curry , D. K. Owens , et al., “Screening for Prostate Cancer: US Preventive Services Task Force Recommendation Statement,” Jama 18 (2018): 1901–1913.

[233] P. Rajwa , F. Quhal , B. Pradere , et al., “Prostate Cancer Risk, Screening and Management in Patients With Germline BRCA1/2 Mutations,” Nature Reviews Urology 4 (2023): 205–216. 10.1038/s41585-022-00680-4 36600087

[234] S. Carlsson , M. Assel , D. Sjoberg , et al., “Influence of Blood Prostate Specific Antigen Levels at Age 60 on Benefits and Harms of Prostate Cancer Screening: Population Based Cohort Study,” Bmj (Clinical Research edition) 348 (2014): g2296. 10.1136/bmj.g2296 PMC3968958 24682399

[235] A. J. Vickers , D. Ulmert , D. D. Sjoberg , et al., “Strategy for Detection of Prostate Cancer Based on Relation Between Prostate Specific Antigen at Age 40–55 and Long Term Risk of Metastasis: Case‐Control Study,” Bmj (Clinical Research edition) 346 (2013): f2023. 10.1136/bmj.f2023 PMC3933251 23596126

[236] E. Kovac , S. V. Carlsson , H. Lilja , et al., “Association of Baseline Prostate‐Specific Antigen Level With Long‐Term Diagnosis of Clinically Significant Prostate Cancer Among Patients Aged 55 to 60 Years: A Secondary Analysis of a Cohort in the Prostate, Lung, Colorectal, and Ovarian (PLCO) Cancer Screening Trial,” JAMA Network Open 3, no. 1 (2020): e1919284. 31940039 10.1001/jamanetworkopen.2019.19284 PMC6991265

[237] R. Arnsrud Godtman , E. Holmberg , H. Lilja , et al., “Opportunistic Testing Versus Organized Prostate‐Specific Antigen Screening: Outcome After 18 Years in the Göteborg Randomized Population‐Based Prostate Cancer Screening Trial,” European Urology 3 (2015): 354–360. 10.1016/j.eururo.2014.12.006 25556937

[238] P. F. Pinsky , H. L. Parnes , and G. Andriole , “Mortality and Complications After Prostate Biopsy in the Prostate, Lung, Colorectal and Ovarian Cancer Screening (PLCO) Trial,” BJU International 2 (2014): 254–259. 10.1111/bju.12368 PMC3873374 24053621

[239] T. P. Kilpela¨inen , T. L. Tammela , M. Roobol , et al., “False‐Positive Screening Results in the European Randomized Study of Screening for Prostate Cancer,” European Journal of Cancer (Oxford, England: 1990) 18 (2011): 2698–2705. 10.1016/j.ejca.2011.06.055 21788129

[240] D. Obiora , O. Orikogbo , B. J. Davies , et al., “Controversies in Prostate Cancer Screening,” Urologic Oncology 1 (2025): 49–53. 10.1016/j.urolonc.2024.06.022 39127529

[241] A. W. Partin , M. K. Brawer , E. N. Subong , et al., “Prospective Evaluation of Percent Free‐PSA and Complexed‐PSA for Early Detection of Prostate Cancer,” Prostate Cancer and Prostatic Diseases 4 (1998): 197–203. 10.1038/sj.pcan.4500232 12496895

[242] M. Lein , F. Koenig , K. Jung , et al., “The Percentage of Free Prostate Specific Antigen Is an Age‐Independent Tumour Marker for Prostate Cancer: Establishment of Reference Ranges in a Large Population of Healthy Men,” British Journal of Urology 2 (1998): 231–236. 10.1046/j.1464-410x.1998.00723.x 9722758

[243] J. Yang , A. Tang , S. Zhang , et al., “The Age‐Specific Reference Intervals for tPSA, fPSA, and %fPSA in Healthy Han Ethnic Male,” Journal of Clinical Laboratory Analysis 4 (2017): e22062. 10.1002/jcla.22062 PMC6817287 27645821

[244] V. Kasivisvanathan , A. S. Rannikko , M. Borghi , et al., “MRI‐Targeted or Standard Biopsy for Prostate‐Cancer Diagnosis,” The New England Journal of Medicine 19 (2018): 1767–1777. 10.1056/NEJMoa1801993 PMC9084630 29552975

[245] O. Rouviere , P. Puech , R. Renard‐Penna , et al., “Use of Prostate Systematic and Targeted Biopsy on the Basis of Multiparametric MRI in Biopsy‐Naive Patients (MRI‐FIRST): A Prospective, Multicentre, Paired Diagnostic Study,” The Lancet Oncology 1 (2019): 100–109. 10.1016/S1470-2045(18)30569-2 30470502

[246] M. van der Leest , E. Cornel , B. Israel , et al., “Head‐To‐Head Comparison of Transrectal Ultrasound‐Guided Prostate Biopsy Versus Multiparametric Prostate Resonance Imaging With Subsequent Magnetic Resonance‐Guided Biopsy in Biopsy‐Naïve Men With Elevated Prostate‐Specific Antigen: A Large Prospective Multicenter Clinical Study,” European Urology 4 (2019): 570–578. 10.1016/j.eururo.2018.11.023 30477981

[247] L. Klotz , J. Chin , P. C. Black , et al., “Comparison of Multiparametric Magnetic Resonance Imaging‐Targeted Biopsy With Systematic Transrectal Ultrasonography Biopsy for Biopsy‐Naive Men at Risk for Prostate Cancer: A Phase 3 Randomized Clinical Trial,” JAMA Oncology 4 (2021): 534–542. 10.1001/jamaoncol.2020.7589 PMC7863017 33538782

[248] N. J. Sathianathen , A. Omer , E. Harriss , et al., “Negative Predictive Value of Multiparametric Magnetic Resonance Imaging in the Detection of Clinically Significant Prostate Cancer in the Prostate Imaging Reporting and Data System Era: A Systematic Review and Meta‐Analysis,” European Urology 3 (2020): 402–414. 10.1016/j.eururo.2020.03.048 32444265

[249] G. Lo , K. R. Burton , M. A. Haider , et al., “Negative Predictive Value of Prostate Multiparametric Magnetic Resonance Imaging Among Men With Negative Prostate Biopsy and Elevated Prostate Specific Antigen: A Clinical Outcome Retrospective Cohort Study,” The Journal of Urology 6 (2019): 1159–1165. 10.1097/JU.0000000000000388 31188731

[250] T. Fazekas , S. R. Shim , G. Basile , et al., “Magnetic Resonance Imaging in Prostate Cancer Screening: A Systematic Review and Meta‐Analysis,” JAMA Oncology 6 (2024): 745–754. 10.1001/jamaoncol.2024.0734 PMC10998247 38576242

[251] J. Richenberg , V. Logager , V. Panebianco , et al., “The Primacy of Multiparametric MRI in Men With Suspected Prostate Cancer,” European Radiology 12 (2019): 6940–6952. 10.1007/s00330-019-06166-z PMC6828624 31172275

[252] S. Veneziano , P. Pavlica , R. Querze , et al., “Correlation Between Prostate‐Specific Antigen and Prostate Volume, Evaluated by Transrectal Ultrasonography: Usefulness in Diagnosis of Prostate Cancer,” European Urology 2 (1990): 112–116. 10.1159/000463885 1699766

[253] M. C. Benson , I. S. Whang , A. Pantuck , et al., “Prostate Specific Antigen Density: A Means of Distinguishing Benign Prostatic Hypertrophy and Prostate Cancer,” The Journal of Urology 147, no. 3 Pt 2 (1992): 815–816. 1371554 10.1016/s0022-5347(17)37393-7

[254] M. Lujan , A. Paez , L. Llanes , et al., “Prostate Specific Antigen Density. Is There a Role for This Parameter When Screening for Prostate Cancer?,” Prostate Cancer and Prostatic Diseases 3 (2001): 146–149. 10.1038/sj.pcan.4500509 12497032

[255] R. W. Allan , H. Sanderson , and J. I. Epstein , “Correlation of Minute (0.5 MM or Less) Focus of Prostate Adenocarcinoma on Needle Biopsy With Radical Prostatectomy Specimen: Role of Prostate Specific Antigen Density,” The Journal of Urology 170, no. 2 Pt 1 (2003): 370–372. 12853777 10.1097/01.ju.0000074747.72993.cb

[256] M. H. Radwan , Y. Yan , J. R. Luly , et al., “Prostate‐Specific Antigen Density Predicts Adverse Pathology and Increased Risk of Biochemical Failure,” Urology 6 (2007): 1121–1127. 10.1016/j.urology.2007.01.087 17572199

[257] N. Omri , M. Kamil , K. Alexander , et al., “Association Between PSA Density and Pathologically Significant Prostate Cancer: The Impact of Prostate Volume,” The Prostate 16 (2020): 1444–1449. 10.1002/pros.24078 32970856

[258] X. Filella and N. Giménez , “Evaluation of [‐2] proPSA and Prostate Health Index (phi) for the Detection of Prostate Cancer: A Systematic Review and Meta‐Analysis,” Clinical Chemistry and Laboratory Medicine 4 (2013): 729–739. 10.1515/cclm-2012-0410 23154423

[259] S. Loeb , “Prostate Cancer: Prostate Health Index–Improving Screening in Men With Family History,” Nature Reviews Urology 9 (2013): 497–498. 10.1038/nrurol.2013.169 23938945

[260] W. J. Catalona , A. W. Partin , M. G. Sanda , et al., “A Multicenter Study of [‐2]Pro‐Prostate Specific Antigen Combined With Prostate Specific Antigen and Free Prostate Specific Antigen for Prostate Cancer Detection in the 2.0 to 10.0 Ng/Ml Prostate Specific Antigen Range,” The Journal of Urology 5 (2011): 1650–1655. 10.1016/j.juro.2010.12.032 PMC3140702 21419439

[261] C. de la Calle , D. Patil , J. T. Wei , et al., “Multicenter Evaluation of the Prostate Health Index to Detect Aggressive Prostate Cancer in Biopsy Naïve Men,” The Journal of Urology 1 (2015): 65–72. 10.1016/j.juro.2015.01.091 PMC4696043 25636659

[262] N. Fossati , M. Lazzeri , A. Haese , et al., “Clinical Performance of Serum Isoform [‐2]proPSA (p2PSA), and Its Derivatives %p2PSA and the Prostate Health Index, in Men Aged <60 Years: Results From a Multicentric European Study,” BJU International 6 (2015): 913–920. 10.1111/bju.12718 24589357

[263] B. Ehdaie and S. Carlsson , “Reply to 'Clinical Utility of the Prostate Health Index (phi) for Biopsy Decision Management in a Large Group Urology Practice Setting,” Prostate Cancer and Prostatic Diseases 3 (2018): 446–447. 10.1038/s41391-018-0052-y 29858593

[264] A. Vickers , A. Cronin , M. Roobol , et al., “Reducing Unnecessary Biopsy During Prostate Cancer Screening Using a Four‐Kallikrein Panel: An Independent Replication,” Journal of Clinical Oncology: Official Journal of the American Society of Clinical Oncology 15 (2010): 2493–2498. 10.1200/JCO.2009.24.1968 PMC2881727 20421547

[265] A. J. Vickers , A. Gupta , C. J. Savage , et al., “A Panel of Kallikrein Marker Predicts Prostate Cancer in a Large, Population‐Based Cohort Followed for 15 Years Without Screening,” Cancer Epidemiology, Biomarkers & Prevention: A Publication of the American Association for Cancer Research, Cosponsored by the American Society of Preventive Oncology 2 (2011): 255–261. 10.1158/1055-9965.EPI-10-1003 PMC3035761 21148123

[266] D. J. Parekh , S. Punnen , D. D. Sjoberg , et al., “A Multi‐Institutional Prospective Trial in the USA Confirms That the 4Kscore Accurately Identifies Men With High‐Grade Prostate Cancer,” European Urology 3 (2015): 464–470. 10.1016/j.eururo.2014.10.021 25454615

[267] R. J. Bryant , D. D. Sjoberg , A. J. Vickers , et al., “Predicting High‐Grade Cancer at Ten‐Core Prostate Biopsy Using Four Kallikrein Markers Measured in Blood in the ProtecT Study,” Journal of the National Cancer Institute 107, no. 7 (2015): djv095. 25863334 10.1093/jnci/djv095 PMC4554254

[268] A. Josefsson , M. Månsson , K. Kohestani , et al., “Performance of 4Kscore as a Reflex Test to Prostate‐Specific Antigen in the GÖTEBORG‐2 Prostate Cancer Screening Trial,” European Urology 3 (2024): 223–229. 10.1016/j.eururo.2024.04.037 PMC11747930 38772787

[269] S. M. Zappala , P. T. Scardino , D. Okrongly , et al., “Clinical Performance of the 4Kscore Test to Predict High‐Grade Prostate Cancer at Biopsy: A Meta‐Analysis of Us and European Clinical Validation Study Results,” Reviews in Urology 3 (2017): 149–155. 10.3909/riu0776 PMC5737341 29302237

[270] B. Konety , S. M. Zappala , D. J. Parekh , et al., “The 4Kscore® Test Reduces Prostate Biopsy Rates in Community and Academic Urology Practices,” Reviews in Urology 4 (2015): 231–240. PMC4735670 26839521

[271] E. A. Vertosick , C. Häggström , D. D. Sjoberg , et al., “Prespecified 4‐Kallikrein Marker Model at Age 50 or 60 for Early Detection of Lethal Prostate Cancer in a Large Population Based Cohort of Asymptomatic Men Followed for 20 Years,” The Journal of Urology 2 (2020): 281–288. 10.1097/JU.0000000000001007 PMC8423096 32125228

[272] K. Smith‐Byrne , G. K. Fensom , U. Noor , et al., “Evaluation of the 4Kscore Test in Relation to Subsequent Risk of Aggressive Prostate Cancer in the European Prospective Investigation Into Cancer and Nutrition,” Cancer Epidemiology, Biomarkers & Prevention: A Publication of the American Association for Cancer Research, Cosponsored by the American Society of Preventive Oncology 11 (2025): 2058–2067. 10.1158/1055-9965.EPI-24-1877 PMC12580810 40844611

[273] K. K. Chevli , M. Duff , P. Walter , et al., “Urinary PCA3 as a Predictor of Prostate Cancer in a Cohort of 3,073 Men Undergoing Initial Prostate Biopsy,” The Journal of Urology 6 (2014): 1743–1748. 10.1016/j.juro.2013.12.005 24333241

[274] J. McKiernan , M. J. Donovan , V. O'Neill , et al., “A Novel Urine Exosome Gene Expression Assay to Predict High‐Grade Prostate Cancer at Initial Biopsy,” JAMA Oncology 7 (2016): 882–889. 10.1001/jamaoncol.2016.0097 27032035

[275] J. McKiernan , M. J. Donovan , E. Margolis , et al., “A Prospective Adaptive Utility Trial to Validate Performance of a Novel Urine Exosome Gene Expression Assay to Predict High‐Grade Prostate Cancer in Patients With Prostate‐Specific Antigen 2–10 ng/Ml at Initial Biopsy,” European Urology 6 (2018): 731–738. 10.1016/j.eururo.2018.08.019 30237023

[276] R. Tutrone , M. J. Donovan , P. Torkler , et al., “Clinical Utility of the Exosome Based ExoDx Prostate(IntelliScore) EPI Test in Men Presenting for Initial Biopsy With a PSA 2–10 Ng/mL,” Prostate Cancer and Prostatic Diseases 4 (2020): 607–614. 10.1038/s41391-020-0237-z PMC7655505 32382078

[277] R. Tutrone , B. Lowentritt , B. Neuman , et al., “ExoDx Prostate Test as a Predictor of Outcomes of High‐Grade Prostate Cancer—An Interim Analysis,” Prostate Cancer and Prostatic Diseases 3 (2023): 596–601. 10.1038/s41391-023-00675-1 PMC10449627 37193776

[278] S. A. Tomlins , J. R. Day , R. J. Lonigro , et al., “Urine TMPRSS2:ERG Plus PCA3 for Individualized Prostate Cancer Risk Assessment,” European Urology 1 (2016): 45–53. 10.1016/j.eururo.2015.04.039 PMC4644724 25985884

[279] J. J. Tosoian , B. J. Trock , T. M. Morgan , et al., “Use of the MyProstateScore Test to Rule out Clinically Significant Cancer: Validation of a Straightforward Clinical Testing Approach,” The Journal of Urology 3 (2021): 732–739. 10.1097/JU.0000000000001430 PMC8189629 33080150

[280] J. J. Tosoian , Y. Zhang , L. Xiao , et al., “Development and Validation of an 18‐Gene Urine Test for High‐Grade Prostate Cancer,” JAMA Oncology 6 (2024): 726–736. 10.1001/jamaoncol.2024.0455 PMC11190811 38635241

